# Evidence for multiple prion conformers in natural scrapie isolates

**DOI:** 10.1371/journal.ppat.1014283

**Published:** 2026-09-17

**Authors:** Morikazu Imamura, Kohtaro Miyazawa, Hiroyuki Okada, Minako Ohno, Hiromi Iguchi, Yuichi Matsuura, Yoshifumi Iwamaru, Hanae Takatsuki, Tsuyoshi Mori, Jiyan Ma, Ryuichiro Atarashi

**Affiliations:** 1 Division of Microbiology, Department of Infectious Diseases, Faculty of Medicine, University of Miyazaki, Miyazaki, Japan; 2 Division of Zoonosis Research, National Institute of Animal Health (NIAH), National Agriculture and Food Research Organization (NARO), Tsukuba, Ibaraki, Japan; 3 Division of Infectious Animal Disease Research, National Institute of Animal Health (NIAH), National Agriculture and Food Research Organization (NARO), Tsukuba, Ibaraki, Japan; 4 Division of Biofunction Analysis, Department of Biotechnology, Frontier Science Research Center, University of Miyazaki, Miyazaki, Japan; 5 Comprehensive Technology Center, University of Miyazaki, Miyazaki, Japan; 6 Beijing Institute for Brain Research, Chinese Academy of Medical Sciences & Peking Union Medical College, Beijing, China; 7 Chinese Institute for Brain Research, Beijing (CIBR), Beijing, China; Creighton University, UNITED STATES OF AMERICA

## Abstract

Prion strains in infected animals may exist as heterogeneous populations composed of a major prion conformer responsible for the dominant disease phenotype and minor conformers that are phenotypically silent or difficult to detect. However, the presence and biological significance of such minor conformers in natural prion isolates remain poorly understood. Identifying and characterizing these hidden substrains is important for understanding prion strain diversity and for assessing potential transmission risks to humans and livestock. In this study, we utilized a modified protein misfolding cyclic amplification (PMCA) method to detect substrains within classical scrapie isolates. Our analysis provided evidence consistent with the coexistence of multiple prion conformers, including previously unrecognized conformers, within single prion isolates. Furthermore, we observed changes in the detectable substrain composition during passages in animal hosts. These findings support the idea that scrapie prion populations can contain diverse conformers and that changes in detectable conformer populations during passage may contribute to the overall diversity of scrapie prions.

## Author summary

Prions are infectious proteins that can cause fatal neurodegenerative diseases in humans and animals. Different prion strains can produce different disease patterns, even when the host prion protein has the same amino acid sequence. Natural prion infections may contain not only the major strain that determines the apparent disease type, but also minor hidden variants that are difficult to detect. These hidden variants may become important when prions pass into a new host or are transmitted repeatedly. In this study, we used a highly sensitive *in vitro* amplification method to examine scrapie prions from naturally and experimentally infected animals. This approach allowed us to detect prion variants that were not readily visible by conventional biochemical methods. Our results provide evidence that scrapie isolates can contain multiple prion conformers and that the detectable composition of these conformers can change during passage. These findings suggest that hidden prion diversity may contribute to changes in prion strain properties during transmission, although the results of *in vitro* amplification should be interpreted together with biological data from animal studies.

## Introduction

Transmissible spongiform encephalopathies (TSEs) are fatal neurodegenerative disorders affecting humans and a broad range of mammals. These diseases are caused by PrP^Sc^, a misfolded and protease-resistant isoform of the host-encoded cellular prion protein (PrP^C^). The pathological propagation and accumulation of PrP^Sc^ within the central nervous system trigger widespread neuronal degeneration and loss, ultimately leading to death.

TSEs occur in diverse animal species, and even within a single host species, multiple disease forms can be identified by their characteristic clinical features, neuropathology, and biochemical profiles. These differences arise from prion strains—conformational variants of PrP^Sc^ despite the conserved primary sequence of the prion protein [1–6]. Each prion strain induces a single and specific disease phenotype in infected hosts, that is stably maintained upon serial transmission.

Scrapie, the earliest TSE, serves as the prototype of animal TSEs. Strain variability within scrapie is suggested by the coexistence of a prion population with distinct properties in certain scrapie-affected animals [4]. For instance, some classical scrapie isolates may harbor CH1641-like scrapie prion strains in a latent form, whereas naturally occurring CH1641-like scrapie isolates can contain classical scrapie prion strains in a phenotypically silent state [7–9]. These minority populations, often referred to as substrains, are variant prion populations that coexist within a dominant strain. While scrapie-affected animals usually manifest the phenotype of the major strain, their own biochemical and biological properties remain cryptic or silent. Conventional methods such as western blotting or immunohistochemistry are insufficient to directly detect substrains; the presence of substrains is typically inferred only when novel disease phenotypes emerge following animal bioassays. Consequently, substrains that do not manifest distinct phenotypes in bioassays remain hidden. Importantly, substrains may exhibit higher transmissibility or cause more severe diseases in non-host animal species, indicating their potential impact on disease transmission and evolution. Currently, animal bioassays remain the only reliable method for confirming the presence of substrains. However, these assays are hindered by high costs, prolonged detection times, and limited sensitivity. To fully understand the diversity of prion strains and the mechanisms driving the emergence of novel strains, there is an urgent need for an *in vitro* technique capable of rapidly and efficiently detecting cryptic substrains that escape traditional detection methods.

Protein misfolding cyclic amplification (PMCA) is a powerful *in vitro* technique that amplifies minute quantities of prions using PrP^C^ as a substrate [10]. Under certain conditions, seeded PMCA products can preserve key strain-associated properties of the original seed, indicating that PMCA can recapitulate some aspects of prion propagation, although amplification outcomes may depend on the substrate and reaction conditions [11]. PMCA therefore represents a promising approach for selectively amplifying and identifying non-manifesting prion substrains that remain undetectable by conventional bioassays. For instance, PMCA using bovinized transgenic mouse brain homogenate (BH) as a substrate and atypical scrapie-affected BH as a seed successfully amplified a C-BSE–like prion. This finding is consistent with bioassay data showing that serial passages of certain atypical scrapie isolates in bovinized mice give rise to C-BSE–like prions [12]. This work illustrates that BSE-like prion components may be hidden within small-ruminant prion isolates and become detectable under specific amplification or transmission conditions. In addition, in sheep experimentally co-inoculated with scrapie and BSE, Western blotting detected only a dominant scrapie-like PrP^Sc^ phenotype in some animals, whereas sPMCA revealed BSE prions in brain and/or lymphoreticular tissues, indicating that BSE prions can coexist with, and be masked by, a dominant scrapie phenotype [13]. These findings indicate that PMCA can be used for substrain identification.

In this study, we aimed to experimentally investigate prion diversity and substrain dynamics in naturally occurring and experimentally transmitted scrapie isolates, focusing on whether individual isolates contain mixtures of phenotypically distinct prion conformers whose detectable composition changes during passage. For this purpose, we established an enhanced PMCA system capable of amplifying otherwise hidden substrains from natural animal prion isolates. Our optimized PMCA method amplified diverse prion strains from various animal sources and enabled strain typing of classical scrapie isolates, with a high degree of correlation to the bioassay results. Using this approach, we detected multiple prion conformers, including previously unrecognized conformers, in several classical scrapie and CH1641-like scrapie isolates, and observed changes in detectable conformer populations during repeated animal passages. These findings provide evidence consistent with the presence of multiple prion conformers within scrapie isolates and with dynamic changes in their detectable compositions during passage.

## Results

### Heparin and digitonin enhance amplification of proteinase K (PK)-resistant PrP from multiple prion strains in PMCA

**Fig 1:**
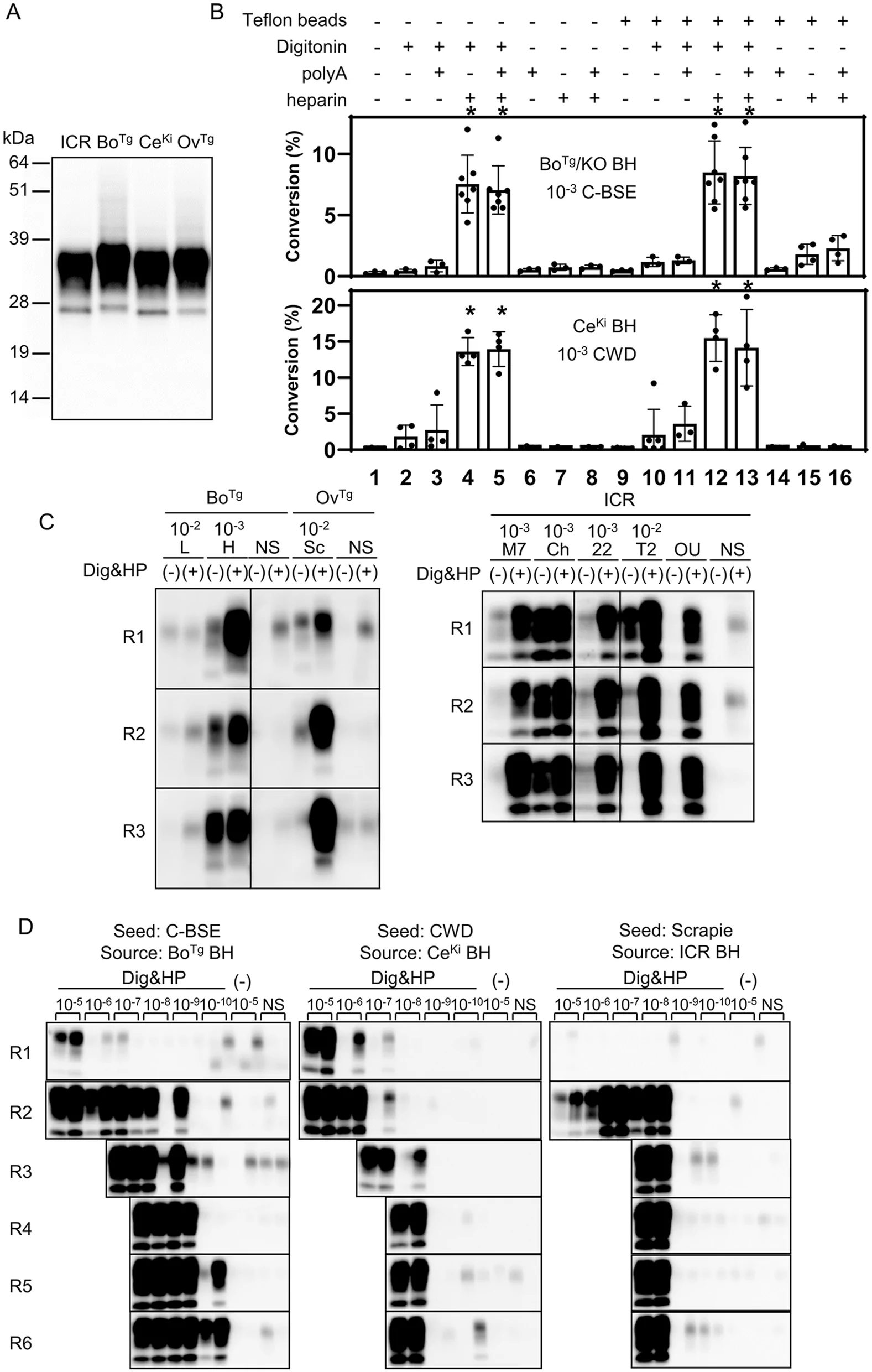
Effects of digitonin and heparin on PMCA amplification of PrP^Sc^-like proteinase K (PK)-resistant PrP (PrPres). **(A)** One microliter of 10% brain homogenates (BHs) from Institute for Cancer Research (ICR) mice, cervid PrP knock-in mice (Ce^Ki^), ovine PrP transgenic mice (Ov^Tg^), and bovine PrP transgenic mice (Bo^Tg^; diluted with 10% *Prnp*-knockout mouse BH) was analyzed by SDS-PAGE and western blotting (WB) using the SAF32 antibody (Cayman Chemical, Ann Arbor, MI, USA). **(B)** PMCA was performed in the presence of indicated additives (Teflon beads, digitonin, synthetic polyA, and heparin). C-BSE– or CWD–infected BHs were diluted 1:1000 with Bo^Tg^/KO or Ce^Ki^ BHs, respectively. After PK digestion and WB, PrPres signals were quantified by densitometry. Data from 3–7 independent experiments were normalized to 100% based on the total PrP signal in the BH; each closed circle represents one independent experiment. Samples #4, 5, 12, and 13 did not differ significantly from one another but differed from all other conditions (p < 0.01). **(C)** PMCA was performed for three rounds in the presence of digitonin and heparin (without Teflon beads) using Bo^Tg^/KO, Ov^Tg^, and ICR BHs as PrP^C^ substrates. Seed labels indicate L-BSE (L), H-BSE (H), classical scrapie (Sc), ME7 (M7), Chandler (Ch), 22L (22), Tsukuba-2 (T2), and OSU (OU). NS, non-seeded controls. 10^-2^ and 10^-3^ indicate 1:100 and 1:1000 dilutions of infected BHs used as PrP^Sc^ seeds, respectively. For OSU, 1 µL of undiluted PMCA product was used as PrP^Sc^ seed. “−“ and “+” indicate the absence and presence of digitonin and heparin, respectively. **(D)** C-BSE–, CWD–, and classical scrapie–infected BHs were serially diluted (10^5^ to 10^10^-fold) into Bo^Tg^, Ce^Ki^, and ICR BHs, respectively. PMCA was performed for six rounds in the presence of digitonin and heparin (Dig&HP). Additional reactions using 10^5^-fold diluted BHs were performed without digitonin and heparin (−). NS, non-seeded controls.

To optimize PMCA for efficient *in vitro* propagation of PrP^Sc^-like conformers across prion strains from cattle, sheep, cervids, and rodents, we evaluated the effects of Teflon beads [14], digitonin [15], synthetic polyA [16–18], and heparin [19,20], both individually and in combination. These factors were tested for their ability to enhance PrP^Sc^-like PK-resistant PrP conformers (hereafter referred to as PrPres) amplification in PMCA. Brain homogenates (BHs) from wild-type (Institute of Cancer Research [ICR]) mice, transgenic mice overexpressing bovine *PrP* (Bo^Tg^), knock-in mice expressing cervid *PrP* (Ce^Ki^), and transgenic mice overexpressing ovine *PrP* (Ov^Tg^) were used as PrP^C^ substrates, with PrP^C^ expression levels adjusted for consistency (Fig 1A).

Initial PMCA experiments using homologous PrP^C^-PrP^Sc^ combinations identified digitonin and heparin as the most effective amplification enhancers. In reactions seeded with C-BSE or chronic wasting disease (CWD) PrP^Sc^, these additives significantly improved PrPres amplification, whereas Teflon beads and polyA had no notable effect (Fig 1B). Extended evaluation across eight prion strains confirmed the broad applicability of digitonin and heparin, particularly for H-BSE, classical scrapie, ME7, 22L, Tsukuba-2 (Tsu-2) [21], and Ohio State University (OSU) [17] prions (Fig 1C). In contrast, Chandler PrPres exhibited minimal further enhancement, indicating that efficient amplification proceeds even in the absence of these additives. Notably, PrPres amplification seeded with L-BSE PrP^Sc^ remained unsuccessful under these conditions.

Sensitivity analysis using serial dilutions of infected BHs demonstrated PrPres amplification from C-BSE, CWD, and classical scrapie seeds diluted up to 10^10^-, 10^8^-, and 10^8^-fold, respectively (Fig 1D). The maximum detectable dilution was reached after five PMCA rounds for C-BSE, after three rounds for CWD, and after two rounds for classical scrapie, with no further increase in detection limits in subsequent rounds. These results confirm that digitonin and heparin significantly enhance PMCA sensitivity across multiple prion strains from different animal species, except for L-BSE prions. Furthermore, no spontaneous PrPres formation was observed in non-seeded PMCA reactions using Bo^Tg^/KO, Ov^Tg^, Ce^Ki^, or ICR BHs (S1 Fig).

### Modified PMCA method preserves strain properties of the initial seed

**Fig 2:**
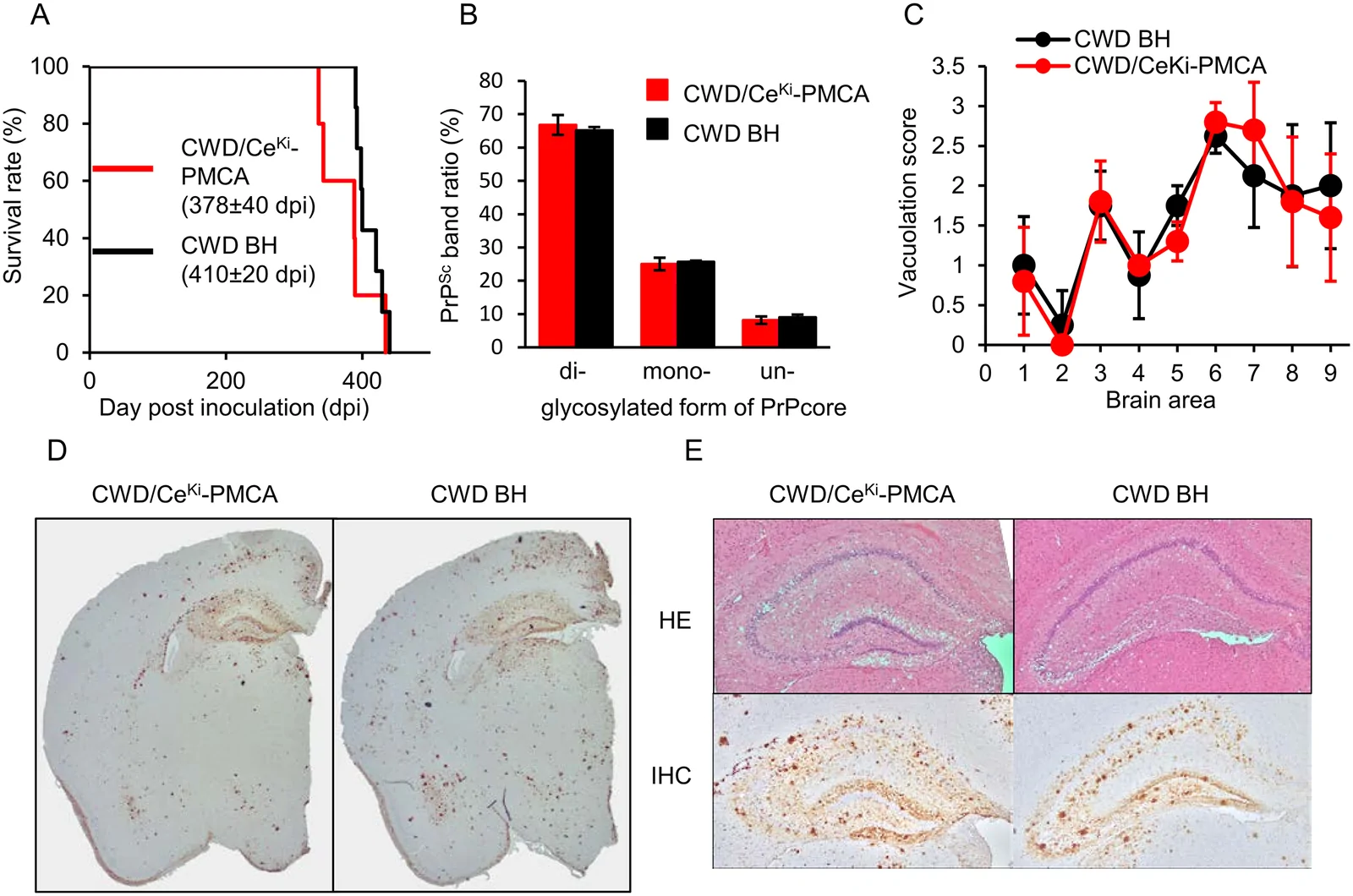
Biochemical and histopathological properties of Ce^Ki^ mice inoculated with PMCA products containing digitonin and heparin. **(A)** Survival curves of Ce^Ki^ mice intracerebrally inoculated with the 12^th^-round CWD-seeded PMCA product (n = 5) or 1% CWD-infected elk BHs (n = 7). Mean survival ± standard deviation (SD) is shown. No significant differences were observed (p = 0.1665). **(B)** Glycoform profiles of PrP^Sc^ (diglycosylated (di-), monoglycosylated (mono-), and unglycosylated (un-) forms) in Ce^Ki^ mice inoculated with PMCA products or CWD-infected BHs. Signals were detected using mAb T2-HRP and quantified densitometrically. Values represent mean ± SD from four replicates. No significant differences were detected (p > 0.05). **(C)** Vacuolation profiles in nine brain regions of Ce^Ki^ mice inoculated with PMCA product or CWD-infected BHs. Brain regions were labeled as follows: 1, dorsal medulla; 2, cerebellar cortex; 3, superior colliculus; 4, hypothalamus; 5, thalamus; 6, hippocampus; 7, septal nuclei of the paraterminal body; 8, cerebral cortex at the levels of 4 and 5; 9, the cerebral cortex at the level of 7. Lesion scores are mean ± SD (n = 5 and 7, respectively). No significant differences were observed in any regions (p-values:1, 0.999; 2, 0.993; 3, 4, 6, 8, > 0.9999; 5, 0.9519; 7, 0.8213; 9, 0.9771). **(D)** Immunohistochemical detection of PrP^Sc^ in brain hemispheres from Ce^Ki^ mice inoculated with PMCA products and infected BHs. **(E)** Representative hippocampal pathology showing vacuolation (HE staining) and PrP^Sc^ deposition (IHC) in mice inoculated with PMCA products or BH.

To assess the infectivity and strain fidelity of PMCA products generated with digitonin and heparin, we performed a mouse bioassay. One-tenth diluted products from the 12th PMCA round, amplified using CWD-infected BH as the seed and Ce^Ki^ BH as the substrate, were intracerebrally inoculated into Ce^Ki^ mice. All inoculated mice developed clinical symptoms and succumbed to disease at an average of 378 ± 40 days post-inoculation, with survival times comparable to those inoculated with 1% CWD-infected BH (Fig 2A).

PrP^Sc^ banding patterns (Fig 2B and S2 Fig), vacuolation distribution (Fig 2C), and PrP^Sc^ deposition in brains (Fig 2D) were similar in both groups. Notably, severe vacuolation and PrP^Sc^ accumulation were prominent in the hippocampus (Fig 2E). These findings confirm that PMCA products generated with digitonin and heparin retain the strain properties of the original seed.

### Arginine Ethyl Ester (AE) improves amplification efficiency of L-BSE prion in PMCA

**Fig 3:**
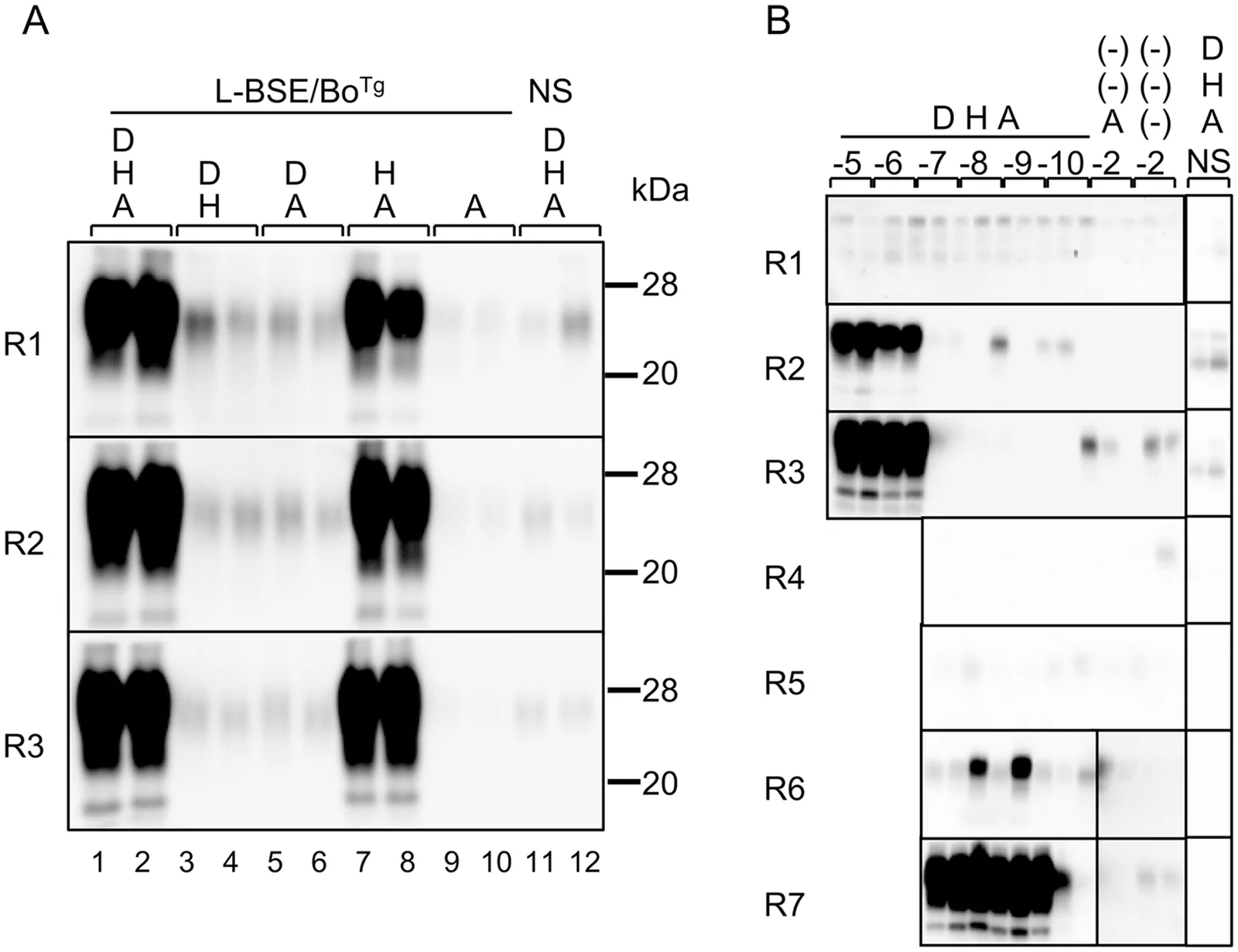
Effect of arginine ethyl ester (AE) on PrPres amplification in L-BSE–seeded PMCA. **(A)** PMCA was performed for three rounds (R1–R3) using Bo^Tg^/KO BH seeded with a 10^3^-fold dilution of L-BSE-infected BH in the presence of 10 mM AE **(A)**, 0.05% digitonin **(D)**, and 300 μg/mL heparin **(H)**. NS, non-seeded controls. Reactions were performed in duplicate. **(B)** L-BSE BH was serially diluted (10^5^-fold to 10^10^-fold) into Bo^Tg^/KO BH, and subjected to seven rounds of PMCA (R1–R7) with AE, digitonin and heparin. As a negative control, PMCA was performed using 10^2^-fold diluted L-BSE-infected BH in the presence or absence of AE without digitonin and heparin. NS, non-seeded control.

PMCA with digitonin and heparin failed to amplify L-BSE PrPres (Fig 1C). Since AE has been shown to facilitate L-BSE PrPres amplification [22], we evaluated its effect in our optimized system. AE enabled L-BSE PrPres amplification in Bo^Tg^/KO BH seeded with a 10^3^-fold dilution of L-BSE-infected BH (Fig 3A). Notably, digitonin was dispensable, as L-BSE PrPres amplified efficiently with heparin and AE alone.

To evaluate detection sensitivity, serial dilutions of L-BSE-infected BH were subjected to PMCA with AE, digitonin, and heparin (Fig 3B). After two PMCA rounds, PrPres was amplified in 10^6^-fold dilutions, and after 7 rounds, in 10^9^-fold dilutions. No *de novo* PrPres formation was detected in the unseeded control reactions performed with AE, digitonin, and heparin.

### Composition of hidden prion conformers in classical scrapie isolates

**Fig 4:**
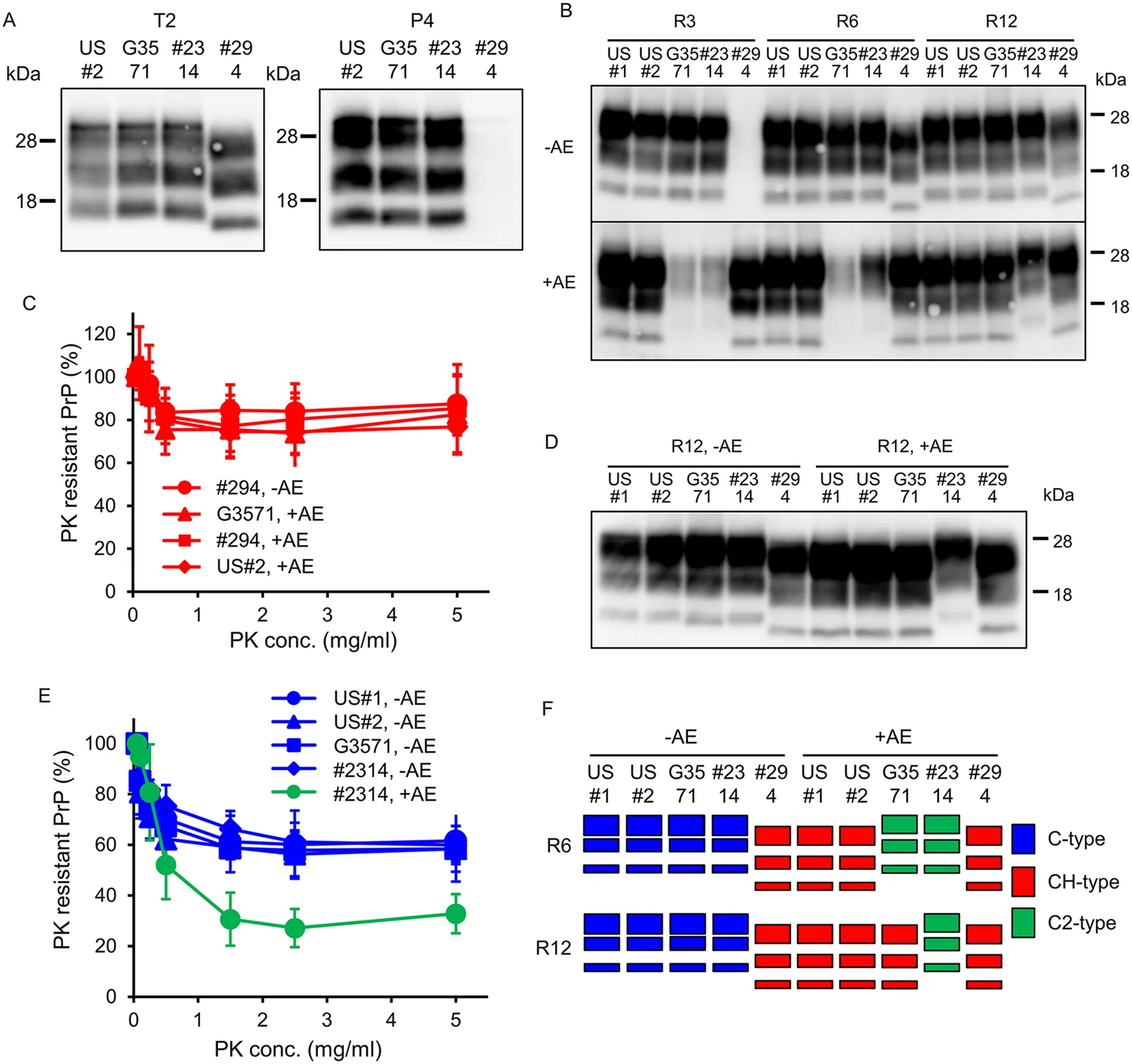
AE alters PMCA amplification products seeded with scrapie isolates. **(A)** WB analysis of PrP^Sc^ from three classical scrapie isolates (US#2, G3571, and #2314) and one CH1641-like scrapie isolate (#294). US#1 isolate was excluded due to insufficient PrP^Sc^ for WB. PrP^Sc^ was detected using the T2 and P4 antibodies. **(B)** Serial PMCA was performed for up to 12 rounds using 100-fold diluted BHs as seed in the presence (DHA) or absence (DH(-A)) of AE; digitonin and heparin were included in all reactions. WB *results* from *rounds* 3, 6, and 12 *are shown.* **(C, E)** PK resistance profiles are shown as mean ±  SE from four independent experiments. CH-type (red), C-type (blue), and C2-type (green) PrPres profiles were analyzed by two-way ANOVA followed by Sidak’s multiple-comparison test, including comparisons within each PrPres type and between C-type and C2-type PrPres. No significant differences were observed within either CH-type or within C-type DH(-A) PMCA products across all PK concentrations. Significant differences were detected between C-type DH(-A) and C2-type DHA products at PK concentrations of 1.5, 2.5, and 5 mg/mL **(E)**. **(D)** Round 12 DH(-A) and DHA PMCA products from (B) were analyzed on a single gel by WB using the T2 antibody. **(F)** Schematic summary of banding patterns observed in rounds 6 and 12 PMCA products from (B) and **(D)**. Blue, red, and green indicate C-type, CH-type, and C2-type PrPres, respectively.

The observation that AE enhanced L-BSE PrPres amplification in PMCA suggests that AE may influence the selective amplification of specific prions during PMCA. It is also well known that multiple scrapie prion conformers coexist in scrapie isolates [7–9]. To investigate whether AE influences the amplification of PrPres seeded with specific scrapie prions of natural or experimental sheep scrapie isolates, we analyzed four classical scrapie isolates: three natural scrapie cases (US#1, US#2, G3571) and one experimental case (#2314). We also analyzed one experimental CH1641-like isolate (#294). Isolates #2314 and #294 were derived from sheep intravenously inoculated with G3571 brain homogenates [7]. Isolates US#2, G3571, and #2314 exhibited similar PrP^Sc^ banding patterns. In contrast, isolate #294 showed a lower molecular mass and lacked P4 antibody reactivity (Fig 4A). These are typical biochemical characteristics of PrP^Sc^ detected in CH1641-like scrapie cases. Due to low PrP^Sc^ levels, isolate US#1 was excluded from WB analysis.

All isolates underwent 12 rounds of serial PMCA in the presence of digitonin and heparin, with or without AE. The results from rounds 3, 6, and 12 are shown in Fig 4B. In DH(-A) PMCA (without AE, -AE), classical scrapie isolates consistently generated PrPres with uniform banding patterns, which were considered to originate from classical scrapie PrP^Sc^. We refer to these products as C-type PrPres. In contrast, CH1641-like isolate #294 failed to amplify PrPres during the first four rounds. However, beginning in the fifth round, it started to produce a lower-molecular-weight PrPres compared to C-type PrPres and this banding pattern persisted in subsequent rounds. We refer to this product as CH-type PrPres. In DHA PMCA (with AE, + AE), PrPres was amplified from the first round, indicating that AE markedly accelerates CH-type PrPres amplification (Fig 4B). This interpretation is further supported by the observation, for isolate #294, PrPres amplified in DHA PMCA exhibited the same PK susceptibility and western blot banding patterns as PrPres amplified in DH(-A) PMCA (Fig 4C and D).

Two distinct PrPres types emerged in DHA PMCA. One was the low-molecular-mass PrPres species, which was predominantly amplified in the early rounds of isolates US#1, US#2 and #294. The other was a high-molecular-mass PrPres species, which was initially amplified in isolates G3571 and #2314. After the seventh round, the PrPres banding pattern of G3571 shifted to the low-molecular-mass type. Notably, the PrPres banding pattern of isolates US#1, US#2, and G3571 in DHA PMCA closely resembled those of isolate #294 (Fig 4D). Together with the PK susceptibility profiles (Fig 4C), these results suggest the amplification of the CH1641-like prion conformer that is present at low levels in the classical isolates US#1, US#2 and G3571.

**Fig 5:**
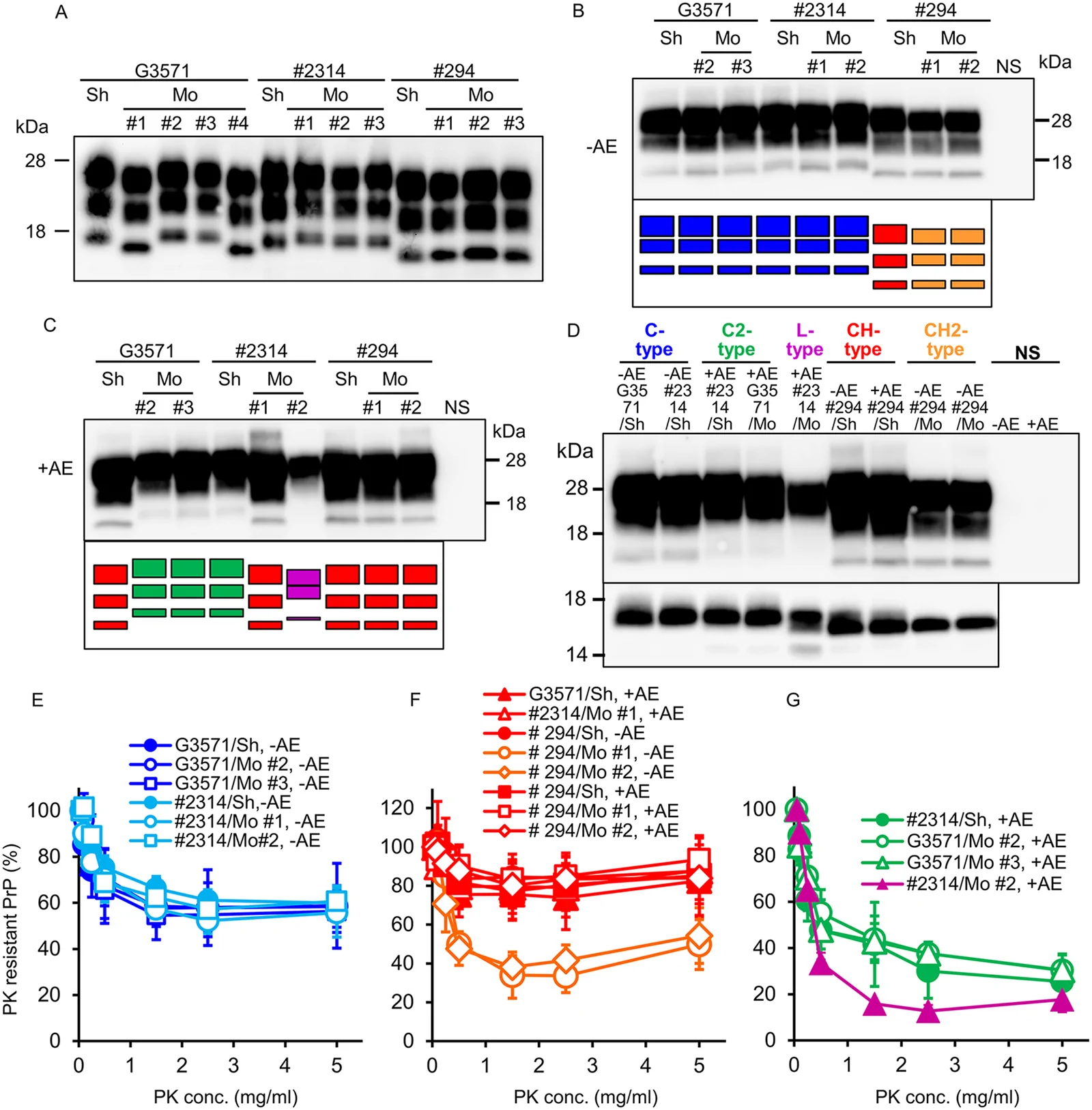
AE-dependent shifts in PMCA amplification using BHs from Ov^Tg^ mice infected with classical or CH1641-like scrapie isolates. **(A)** WB analysis of PrP^Sc^ in brains of Ov^Tg^ mice infected with classical (G3571 and #2314) or CH1641-like (#294) isolates, together with the corresponding sheep inocula. **(B, C)** Twelve rounds of PMCA were performed under DH(-A) (B) or DHA (C) conditions using sheep (Sh) or Ov^Tg^ mouse (Mo) BHs as PrP^Sc^ seeds. WB results from round 12 (R12) are shown (upper panels) along with schematic band representations (lower panels). Numbers correspond to the individual mouse identification numbers shown in **(A)**. Color code: C-type (blue), CH-type (red), CH2-type (orange), C2-type (green), L-type (purple). NS, non-seeded controls. **(D)** Round 12 PMCA products from (B) and (C) were analyzed on the same gel by WB. **(E-G)** PK resistance profiles of round 12 PMCA products are shown as mean ±  SD (3–5 replicates) and analyzed by two-way ANOVA with Sidak’s multiple-comparison test. **(E)** No significant differences were observed among DH(-A) products from the G3571 (blue) and #2314 (sky blue) sheep isolates and their corresponding first-passage mice across all PK concentrations. **(F)** Significant differences were detected between CH-type and CH2-type PMCA products at PK concentrations of 0.5, 1.5, 2.5, and 5 mg/mL, whereas no significant differences were detected within CH-type or CH2-type groups. **(G)** Significant differences were detected between L-type #2314/Mo#2 product and C2-type PMCA products at PK concentrations of 1.5 and 2.5 mg/mL, whereas no significant differences were detected within C2-type groups.

Additional conformers producing a higher-molecular-weight PrPres banding pattern, distinct from CH-type PrPres were preferentially amplified in the early rounds of DHA PMCA for isolates G3571 and #2314. The unglycosylated PrPres from isolate #2314 (+AE #2314/Sh) exhibited an apparent molecular weight comparable to that of C-type PrPres (Fig 5D, lower panel), but showed distinct glycosylation profiles (Fig 5D, upper panel; S3 Fig A) and altered PK susceptibility (Fig 4E). We therefore designated these PrPres species as C2-type PrPres.

To determine whether prolonged serial PMCA using Ov^Tg^ BH could itself result in spontaneous PrPres formation under the digitonin/heparin conditions used above, either in the absence or presence of AE, we performed expanded non-seeded control reactions. Twelve independent non-seeded reactions were serially propagated for 12 rounds under each condition. No spontaneous PrPres formation was detected in any reaction under either condition (0/12 for DH(-A) and 0/12 for DHA; S4 Fig).

A schematic summary of these findings is shown in Fig 4F. Classical scrapie isolates exclusively amplified C-type PrPres in DH(-A) PMCA, whereas DHA PMCA preferentially amplified CH- or C2-type PrPres, with no detectable C-type amplification. Isolate #294 yielded only CH-type PrPres under both conditions, indicating the absence of C- and C2-type prion conformers. Isolates US#1 and US#2 produced C-type PrPres in DH(-A) PMCA and CH-type PrPres in DHA PMCA, suggesting the coexistence of classical scrapie and CH1641-like conformers. Serial PMCA of isolate G3571 resulted in the amplification of all three types, indicating the presence of classical scrapie, CH1641-like, and C2-type conformers. Conversely, isolate #2314 lacked CH-type prions and contained both classical scrapie and C2-type conformers. Collectively, these results demonstrate that AE modulates prion strain preference in PMCA and highlight the diversity of latent prion conformers within scrapie isolates.

### Strain typing using bioassays in Ov^Tg^ and wild-type mice

PMCA analysis predicted that each sheep classical scrapie isolate harbored either CH-type conformers, C2-type conformers, or both as substrains, with isolate #294 predicted to contain only CH-type conformers. To validate these predictions, we conducted *in vivo* bioassays using Ov^Tg^ mice and wild-type mice. BHs from sheep G3571, #2314, and #294 were intracerebrally inoculated into 8, 10, and 10 Ov^Tg^ mice, respectively. All inoculated mice developed terminal disease, confirming the infectivity of the isolates. Western blot analysis of BHs from the inocula and representative Ov^Tg^ mice is shown in Fig 5A. To distinguish sheep- and mouse-passaged isolates, the suffixes “/Sh” and “/Mo” were appended to isolate designations. Strain classification results in Ov^Tg^ mice and their transmissibility to wild-type mice are summarized in Table 1. In the brains of Ov^Tg^ mice inoculated with G3571/Sh, 4 of 8 mice accumulated C-type PrP^Sc^, while the remaining 4 accumulated CH-type PrP^Sc^, indicating the coexistence of both strains, consistent with the PMCA prediction. By contrast, mice inoculated with #2314/Sh accumulated only C-type PrP^Sc^, suggesting the absence of CH-type conformers. On the other hand, neither G3571/Sh nor #2314/Sh induced C2-type PrP^Sc^ accumulation in any inoculated mice. Previous reports have shown that classical scrapie is transmissible to wild-type mice whereas CH1641 is not [7,23,24]. Wild-type mice inoculated with G3571/Sh and #2314/Sh developed terminal disease. In contrast, Ov^Tg^ mice inoculated with #294/Sh accumulated only CH-type PrP^Sc^, and wild-type mice inoculated with #294/Sh neither developed clinical disease nor showed PrP^Sc^ accumulation. These findings demonstrate that #294/Sh harbors only CH-type conformers, in agreement with the PMCA results.

**Table 1:** Prion strain typing of G3571/Sh, #2314/Sh and #294/Sh in brain using bioassay with Ov^Tg^ and ICR mice.

Animal ID	Inoculum (PrPres type)	Ov^Tg^						ICR
		1^st^ passage			2^nd^ passage			1^st^ passage
		Transmission	PrPres type by WB		Transmission	PrPres type by WB		Transmission
			C-type	CH-type		C-type	CH-type	
**G3571**	Brain (C-type)	8/8 ^a^	4 (352 ± 50.4) ^b^	4 (447 ± 86.2)	NA	NA	NA	6/6 (412 ± 24.9)
**#294**	Brain (CH-type)	10/10	0	10 (259 ± 23.3)	5/5	0	5 (233 ± 13.4) [23]	No transmission [7]
**#2314**	Brain (C-type)	10/10	10 (509 ± 38.0)	0	9/9	4 (332 ± 6.3)	5 (316 ± 35.0)	19/19 (398 ± 20.3)

^a^Number of diseased/ inoculated mice.

^b^Survival days ± SD ^c^ NA means “not attempted”.

### Altered substrain composition in scrapie isolates during transmission to Ov^Tg^ mice

To assess the stability of strain characteristics following transmission to Ov^Tg^ mice, we examined the strain composition in two Ov^Tg^ mice inoculated with each of three scrapie isolates using PMCA. The results of PMCA for #2314/Mo and #294/Mo were validated by intracerebrally inoculating Ov^Tg^ mice with BHs derived from each single mouse.

BHs from two Ov^Tg^ mice per group inoculated with scrapie-affected sheep BHs underwent 12 PMCA rounds. PrPres was amplified in all samples, with results for rounds 3, 6, and 12 presented in S5 Fig. DH(-A) and DHA PMCA products from round 12 were analyzed via WB (Fig 5B, C). The DH(-A) PMCA products derived from G3571/Mo and #2314/Mo retained banding patterns and PK sensitivity consistent with their respective inocula (Fig 5B, E). In contrast, #294/Mo-derived DH(-A) PMCA products showed a reduced di-glycosylated PrP ratio compared to #294/Sh (S3 Fig B, Fig 5B, D), despite similar molecular weights of non-glycosylated PrPres (Fig 5D). Additionally, #294/Mo-derived PrPres exhibited reduced PK sensitivity compared to CH-type PrPres from #294/Sh (Fig 5F). These findings led to the designation of #294/Mo-derived PrPres as CH2-type, a conformer distinct from CH-type PrPres.

G3571/Sh contained C2- and CH-type conformers as substrains (Fig 4B, F). Following 12 PMCA rounds, amplification products from two G3571/Mo isolates produced only C2-type PrPres with no detectable CH-type PrPres (Fig 5C and S5 Fig). This suggests that CH1641-like conformers within G3571/Sh were not transmitted to Ov^Tg^ mice with the classical scrapie phenotype.

For #2314/Sh, only C2-type PrPres was amplified in 12 rounds of DHA PMCA (Fig 4B). In #2314/Mo isolates, C2-type PrPres was detected up to round 6, after which CH-type PrPres emerged in #2314/Mo #1 (S5 Fig). In #2314/Mo #2, another PrPres type emerged, characterized by two bands: one of intermediate molecular mass between the C- and CH-types and an additional 15-kDa band (Fig 5D, lower panel). This PrPres showed reduced PK sensitivity compared to C2-type, leading to its designation as L-type (Fig 5G). These findings suggest that intracerebral inoculation of #2314/Sh into Ov^Tg^ mice induced *de novo* generation of CH- and L-type prion conformers. When brain homogenate #2314/Mo#1 was inoculated into nine Ov^Tg^ mice, C-type PrP^Sc^ accumulation was observed in the brains of five mice, while CH-type PrP^Sc^ accumulated in the remaining four (Table 1). These findings corroborate the PMCA results, which suggest the generation of CH-type conformers in #2314/Mo#1.

Unlike the DH(-A) PMCA product, the DHA PMCA products of #294/Mo exhibited a banding pattern and PK sensitivity similar to that of the DHA PMCA product of #294/Sh (Fig 5C, F). While natural CH1641-like isolates have been reported to produce both CH1641-like and classical scrapie-type PrP^Sc^ in Ov^Tg^ mice [24], our experiment showed that when #294/Mo#1 was inoculated into Ov^Tg^ mice, all mice exclusively exhibited CH-type PrP^Sc^. These findings suggest that the inoculation of #294/Sh into Ov^Tg^ mice does not result in C-type PrP^Sc^.

## Discussion

Although prion strains typically exhibit well-defined and stable phenotypes, these phenotypes can change during serial passage. Such changes may reflect conformational diversification or adaptation within a dominant strain population, the emergence of newly detectable conformers, or the selection and expansion of previously minor, phenotypically silent substrains. Several experimentally generated prion strains have been proposed to behave as quasispecies-like populations, consisting of related PrP^Sc^ conformational variants that can be generated and selected under defined replication environments [25–29]. This quasispecies-like population structure should be distinguished from the coexistence of discrete strains or substrains within a natural isolate. In natural scrapie, for example, phenotypically distinct prion components may be present as minor or silent substrains that are not readily apparent from the dominant disease phenotype or conventional biochemical analyses. The direct detection and characterization of such hidden conformers in naturally occurring isolates has remained challenging. Here we used PMCA to detect substrains within naturally occurring and experimentally transmitted scrapie isolates that were not readily detectable using conventional analytical approaches. Our findings provide evidence consistent with strain heterogeneity and dynamic changes in natural and experimentally transmitted scrapie isolates, showing that these isolates can contain multiple prion conformers whose detectability and/or relative representation can change during passage. Because these profiles were obtained using PMCA under defined substrate and cofactor conditions, they should be interpreted as PMCA-detectable conformer profiles rather than as a complete or fully quantitative representation of all conformers present *in vivo*.

Optimization of the PMCA reaction system is essential for highly sensitive detection of prion substrains. We found that digitonin and heparin enhanced the amplification of PrPres from various prion strains, while arginine ethyl ester (AE) not only facilitated amplification of L-BSE PrPres but also selectively enhanced the amplification of PrPres associated with CH1641-like conformers within classical scrapie isolates. Digitonin, a non-ionic detergent, can solubilize cholesterol-rich membranes, thereby releasing membrane-bound PrP^C^, suppressing PrP^C^ aggregation, and increasing the pool of soluble, monomeric PrP^C^ available for conversion [15]. Heparin, a highly sulfated glycosaminoglycan that binds both PrP^C^ and PrP^Sc^ [20], may promote productive interactions between substrate and seed [30], facilitate aggregation, and fibril formation as a molecular scaffold [19,20,31]. Together, these effects provide a plausible explanation for the broad enhancement of PrPres amplification by digitonin and heparin. AE, a more hydrophobic arginine derivative, may modulate prion amplification in a conformer-dependent manner. Because PrP^Sc^ exposes hydrophobic surfaces, AE could reduce aggregation of L-BSE– and CH1641-like–derived conformers, thereby stabilizing conversion-competent intermediates and enhancing PrPres amplification. By contrast, classical scrapie conformers may be less compatible with AE, resulting in neutral or inhibitory effects. Thus, AE appears to shift amplification efficiency and preference in a strain-dependent manner, potentially through effects on PrP^Sc^ and/or the conversion environment (including PrP^C^ and cofactors).

The high detection limits observed here are broadly consistent with previous PMCA studies reporting sensitive detection of C-BSE at 10^−8^ to 10^−9^ dilutions, with occasional detection at 10^−10^, CWD at a 6.7 × 10^−13^ dilution of 10% brain homogenate, and sheep scrapie at approximately 10^−6^ to 10^−7^ dilutions under different optimized conditions [32–34]. Because these assays differed in substrate, additives, sonication conditions, and number of amplification rounds, direct numerical comparison should be made cautiously. Nevertheless, these comparisons support the view that the digitonin/heparin conditions used here provide a broadly effective amplification environment for several animal prion strains.

Using this optimized system, we detected not only CH1641-like prion conformers but also previously unrecognized prion conformers in classical scrapie isolates. Notably, substrain composition varied among isolates and changed dynamically during passage in animal hosts. To our knowledge, this is the first study to provide experimental evidence consistent with dynamic change in the composition of phenotypically silent substrains during passage, with specific conformers emerging, diminishing, or becoming undetectable. Such dynamics are likely a key driver of strain diversity and variability in naturally occurring prion diseases, particularly in the case of natural sheep scrapie.

**Fig 6:**
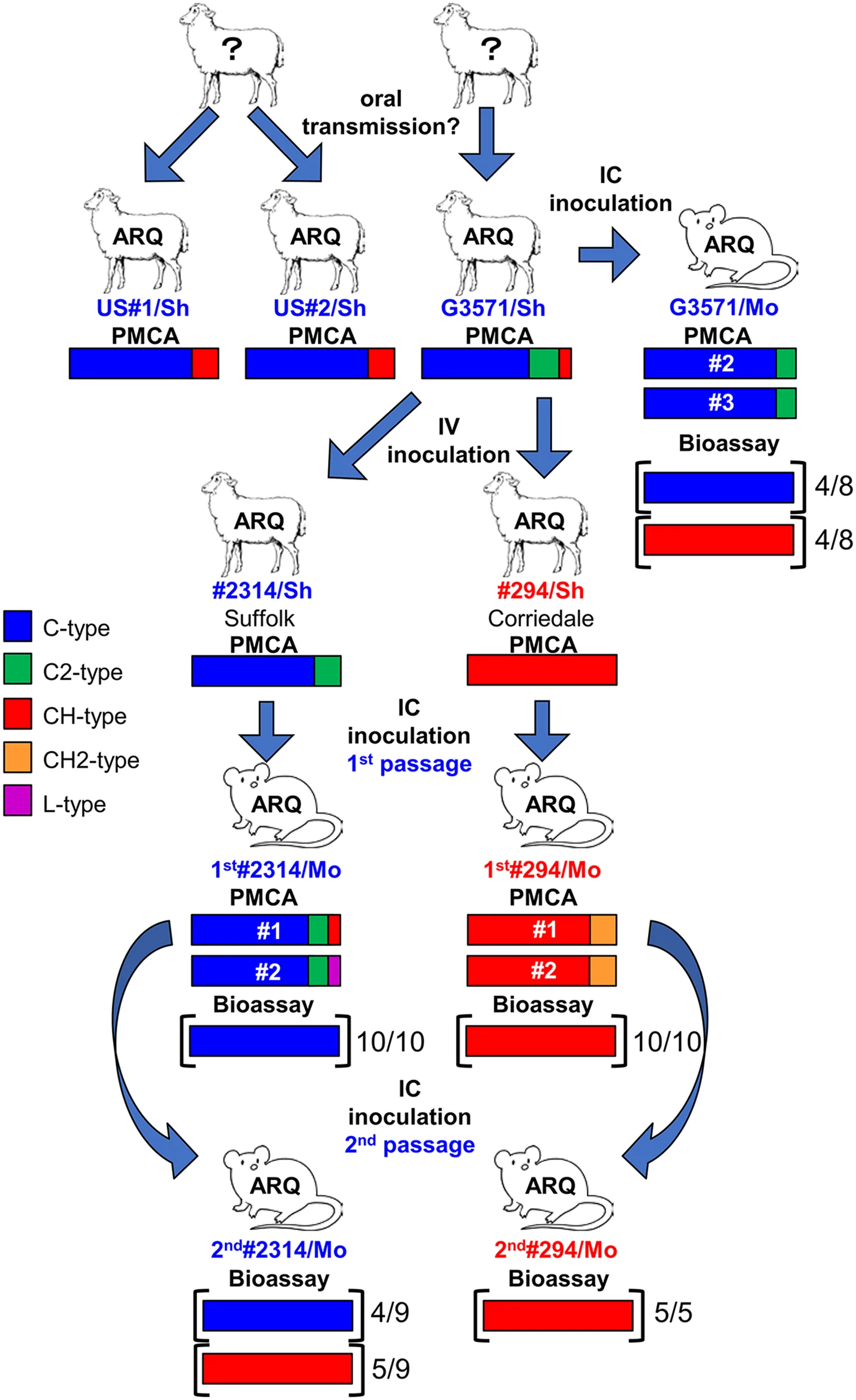
Overview of scrapie strain typing by PMCA and bioassay. Arrows indicate inoculation routes; sheep and mouse illustrations denote infected hosts. Colored bars beneath each illustration indicate inferred prion strain components (C-type: blue, C2-type: green, CH-type: red, CH2-type: orange, L-type: purple) and their relative proportions in isolates. Proportions shown in the bars represent qualitative estimates inferred from PMCA profiles, WB patterns, and bioassay outcomes, not quantitatively measured values. Mouse IDs (#1–3) correspond to those in Fig 5A. Bracketed bars and adjacent numbers indicate bioassay results (Table 1). Blue and red bars indicate strains observed in transgenic mice; numbers denote total inoculated and affected mice.

PMCA typing predicted the presence of the CH-type prion conformer in the brains of a sheep G3571 (G3571/Sh) and an Ov^Tg^ mouse inoculated with sheep #2314 (#2314/Mo#1). In fact, half of the Ov^Tg^ mice inoculated with either G3571/Sh or #2314/Mo#1 developed the CH-type prion disease phenotype (Table 1, Fig 6). In contrast, no CH-type disease developed in Ov^Tg^ mice inoculated with #2314/Sh, which was predicted to lack CH-type conformers by PMCA typing. These results demonstrate a strong correlation between PMCA-based strain discrimination and *in vivo* bioassays, supporting the biological relevance of at least some substrains detected by PMCA, particularly the C- and CH-type conformers.

A limitation of the present study is that PMCA may not fully reproduce the selective pressures that operate during *in vivo* prion propagation. Although seeded PMCA using brain homogenate as the substrate can preserve key strain properties of the original seed, as supported by our CWD bioassay experiment, the outcome of PMCA may be influenced by the substrate and cofactor conditions used for amplification. Consistent with this caveat, recent studies using recombinant prion amplification systems have shown that sulfated glycan cofactors, including heparin, can facilitate the spontaneous emergence of diverse prion conformers *in vitro*, although these cofactors do not necessarily determine specific strain properties [35]. Indeed, in the present study, AE-containing PMCA enabled the preferential detection of CH-type conformers from several classical scrapie isolates, and the biological relevance of these CH-type conformers was supported by the corresponding bioassay results. This finding indicates that defined PMCA conditions can be useful for revealing otherwise cryptic conformers. Conversely, however, conformers that are not efficiently propagated under the same conditions may remain undetected. This point is particularly important when the seed contains a mixture of prion conformers. Under such conditions, PMCA may preferentially amplify conformers that are efficiently propagated in vitro, or conformers that are relatively abundant in the original seed, whereas conformers selected during in vivo transmission may not necessarily be amplified with the same efficiency. Therefore, the PMCA-based conformer profiles described here should not be interpreted as a complete representation of all conformers that may be selected *in vivo*. Rather, they should be viewed as a readout of conformers preferentially amplified under the PMCA conditions used in this study. For this reason, we interpreted the PMCA results together with the bioassay data.

Another caveat is that prolonged serial PMCA may, in principle, allow stochastic *de novo* PrPres formation under specific amplification conditions. To address this possibility, we performed expanded non-seeded controls using Ov^Tg^ BH under the DH and DHA conditions used in this study. No spontaneous PrPres formation was detected in 12 independent reactions under either condition. Although these results argue against frequent spontaneous PrPres generation, they do not completely exclude rare stochastic *de novo* events during prolonged PMCA.

We observed a phenotypic shift from classical scrapie to CH1641-like scrapie during passage. Such changes, often termed “prion strain evolution”, are generally thought to result from adaptation to new transmission environments, such as changes in host species or infection routes (e.g., intracerebral and intravenous inoculation). These environmental factors likely influence the selection of prion conformers capable of expressing distinct phenotypes and may involve cofactors that affect prion structure [36]. Similarly, the emergence and disappearance of substrains may be driven by changes in the amplification environment. However, it is also possible that these apparent changes are due to fluctuations in the relative abundance of substrains, influenced by the detection limits of PMCA. Regardless, our findings support the view that the detectable substrain composition within prion isolates undergoes dynamic and significant changes during serial passage.

The differences in substrain composition observed between first-passage Ov^Tg^ mice (#1 and #2) inoculated with #2314/Sh suggest that stochastic effects may contribute, in addition to environmental variables such as host context and infection route. Such variability could arise from stochastic sampling during transmission—i.e., a founder effect–like process in which only a limited subset of conformers present in the inoculum successfully initiates propagation in the recipient. Consistent with this view, recipients inoculated with mixtures containing both C- and CH-type conformers can differ in which conformer becomes dominant, resulting in distinct phenotypes across individuals. Because both sheep and Ov^Tg^ mice used here were homozygous for the ARQ PrP genotype, PrP sequence polymorphism is unlikely to account for the observed differences.

C2-type PrPres was detectable not only in Ov^Tg^ mice but also in sheep, although it was not consistently amplified from all C-type scrapie isolates. This distribution is consistent with the possibility that C2-type PrP^Sc^ represents a natural minor substrain present in some scrapie isolates, rather than solely an *in vitro*-adapted product of C-type conformers during PMCA. However, because the biological properties of the C2-type conformer have not yet been confirmed by bioassay, its origin should be interpreted cautiously. In contrast, CH2-type PrPres was reproducibly observed only in Ov^Tg^ mice inoculated with CH-type isolates, suggesting that it may have emerged during intracerebral passage or specifically in Ov^Tg^ host environment. Serial passage of #294/Sh in Ov^Tg^ mice resulted in minimal changes in neuropathological features, whereas incubation periods progressively decreased across passages [23]. These findings are compatible with an adaptive process of the CH1641-like prion to the Ov^Tg^ host environment, and the CH2 conformer may be associated with this adaptive process. Nevertheless, further investigation is needed to clarify whether CH2-type PrPres represents a *de novo* conformer, an adaptation of the CH-type conformer, or a conformer preferentially selected under the PMCA conditions used in this study. Similarly, the L-type PrPres conformer was reproducibly detected under defined PMCA conditions, but its biological properties and origin remain to be determined. Thus, unlike the C- and CH-type conformers, whose relevance was supported by the correlation between PMCA typing and bioassay results, the C2-, CH2-, and L-type conformers should be regarded as reproducible PMCA-detectable conformers whose biological significance requires further validation.

The propagation of classical scrapie in wild-type mice is known to generate diverse mouse-adapted scrapie strains [37–39]. Here, we provide evidence for diversity in the composition of latent conformers within classical scrapie and CH1641-like isolates. These findings suggest that latent conformers may potentially replace the dominant phenotypic conformer when propagated in new environments (e.g., a new host species), leading to the emergence of distinct prion diseases in the new host. Collectively, this raises concerns that newly identified conformers, including C2, CH2, and L types, may warrant consideration with respect to potential interspecies transmission risks to other species, such as cattle and humans. To elucidate the risk of interspecies transmission of these hidden prion conformers, bioassays combining PMCA products and transgenic mice expressing the PrP^C^ derived from various animal species will be required.

Our study provides evidence consistent with the presence of diverse prion conformers in natural and experimentally transmitted scrapie isolates, and with changes in the relative representation of detectable conformers during passage. The addition of AE alters the amplification preference of prion conformers in PMCA, allowing for the detection of hidden prion substrains within scrapie isolates. The improved PMCA technique developed in this study provides a cost-effective and time-efficient tool for monitoring the emergence of a new dominant strain in scrapie isolates. Further refinement of this approach may allow us to analyze prions from other species, such as CWD in cervids, which will provide critical insights into interspecies transmission risk.

## Materials and methods

### Ethics statement

All animal experiments were approved by the Committee of Animal Experiments of the National Institute of Animal Health (NIAH), the National Agriculture and Bio-oriented Research Organization (Tsukuba, Ibaraki, Japan) (approval ID:11–008, 13–005). All procedures were conducted in accordance with the guidelines for Animal Experiments by the Ministry of Agriculture, Forestry, and Fisheries of Japan.

### Preparation of PrP^C^ substrates

PrP^C^ substrates were prepared from the brains of 11–13 week-old wild-type ICR mice, transgenic mice lacking the endogenous *Prnp* gene but overexpressing bovine PrP (Bo^Tg^) [40] or ovine ARQ PrP (Ov^Tg^: TgOvPrP59) [41], and knock-in mice expressing cervid PrP (Ce^Ki^). The generation of Ce^Ki^ mice has been previously described [42]. Cervid PrP in Ce^Ki^ mice is expressed as a fusion protein comprising amino acids 1–28 of murine PrP and amino acids 32–256 of cervid PrP. Brain tissue from each individual mouse was homogenized to a 20% (w/v) concentration in sterile 1 × phosphate buffered saline (PBS; Nacalai, Kyoto, Japan). Each brain homogenate (BH) was then mixed with an equal volume of 2 × PMCA buffer (1 × PBS containing 2% Triton X-100 and 8 mM EDTA). For bovine PrP^C^ substrate, 10% BH from Bo^Tg^ was mixed with 10% BH from *Prnp*-knockout mice at a ratio of 2:3. All BHs were stored at –80 °C until use.

### PrP^Sc^ seeds

PrP^Sc^ seeds used in this study included prion-infected BHs and bacterial recombinant PrP^Sc^ generated via PMCA [17] (Ohio State University strain, OSU). Prion-infected brains were homogenized to a 10% (w/v) concentration in 1 × PBS and diluted as necessary. Mouse-adapted prion strains, Chandler, ME7, 22 L, and Tsukuba-2 (Tsu-2, derived from classical scrapie in Japan), were routinely propagated in ICR mice in NIAH. For OSU, recombinant PrP^Sc^ generated by PMCA was utilized. L-type, H-type, and C-type BSEs originated from cattle experimentally infected with Japanese L-type [43], Canadian H-type [44], and British C-type BSEs (provided by VLA, Weybridge, UK), respectively. Classical scrapie and chronic wasting disease (CWD) were provided by Dr. Mary Jo Schmerr (United States Department of Agriculture).

### Protein Misfolding Cyclic Amplification (PMCA)

PMCA was performed using an automatic cross-ultrasonic protein-activating apparatus (ELESTEIN 070-GOT; Elekon Science Corp., Chiba, Japan). Amplification involved 32 cycles of sonication (pulse oscillation for 3 seconds, repeated five times at intervals of 0.1 seconds), followed by incubation at 37 °C for 30 minutes with gentle agitation. The following reagents were added to each BH prior to amplification: digitonin (12333–51; Nacalai, Kyoto, Japan) at 0.05%, synthetic polyA (P9403; Sigma-Aldrich, St. Louis, MO, USA) at 40 μg/mL, heparin (H3393; Sigma-Aldrich) at 300 μg/mL, and AE (A2883; Sigma-Aldrich) at 10 mM. Teflon beads (3 mm) were purchased from Technochemicals (Tokyo, Japan). PMCA products of the first round of amplification were diluted 1:10 with fresh PrP^C^ substrate, and a second round of amplification was performed. This process was repeated whenever necessary.

### Western blotting analysis of PMCA products

PMCA products (2.5 µL) were digested with 40 µg/mL proteinase K at 37 °C for 1 h. Samples were then boiled in sodium dodecyl sulphate (SDS) sample buffer for 5 minutes, separated by SDS-polyacrylamide gel electrophoresis using NuPAGE 12% Bis-Tris gels (Invitrogen, Carlsbad, CA, USA) or 15% Tris-glycine gels, and transferred onto polyvinylidene fluoride (PVDF) membranes. The membranes were probed with anti-PrP horseradish peroxidase-conjugated monoclonal antibody T2 [45] or the P4 monoclonal antibody (R-Biopharm, Almere, Netherlands). Unless otherwise specified, the T2HRP conjugate was used for western blotting. Immunoreactive signals were developed using SuperSignal West Dura Extended Duration Substrate (Pierce, Rockford, IL, USA), and the chemiluminescence signals of PrPres were visualized with a Chemi-Imager (Alpha InnoTec, San Leandro, CA). Signal intensities of the PMCA products were quantified using software provided by Alpha InnoTec.

### Bioassay

Briefly, 10% BH from CWD-infected elk was diluted 1:1,000 with Ce^Ki^ BH as a PrP^C^ substrate and subjected to one round of PMCA. The PMCA product was further amplified through 11 additional rounds of 10-fold serial dilution and amplification. PMCA products were diluted 1:10 with 1 × PBS, and 20 µL of diluted sample was intracerebrally injected into 3-week-old Ce^Ki^ mice under sevoflurane anaesthesia. As a positive control, 1% BH from naturally CWD-infected elks (used as PMCA seed) was similarly inoculated. Additionally, 10% BH from G3571/Sh, #2314/Sh, and #294/Sh, as well as 10% BH from #2314/Sh- and #294/Sh-inoculated Ov^Tg^ mice, were intracerebrally inoculated into 3–5-week-old Ov^Tg^ or ICR mice under sevoflurane anaesthesia. The mice were housed in a biosafety level 3 facility, and their clinical status was monitored at least three times per week. Animals were euthanised with sevoflurane overdose upon showing signs of progressive neurological dysfunction, and their brains were collected. The right hemisphere was fixed in 10% neutral buffered formalin solution (FUJIFILM Wako, Osaka, Japan) for histopathological analysis, while the left hemisphere was stored at –80 °C for biochemical studies. Average survival times for experimental groups were analyzed using one-way analysis of variance (ANOVA) followed by Tukey–Kramer multiple comparison tests.

### Histopathological studies

The right hemisphere of the brain was fixed with 10% neutral buffered formalin solution. Coronal sections of the brain were treated with 98% formic acid to reduce infectivity and embedded in paraffin wax. Sections of 4-µm thickness were prepared and stained with haematoxylin and eosin (HE; *Sakura* Finetek, Tokyo, *Japan*) or subjected to immunohistochemistry. Neuropathological analysis was performed on HE-stained sections, and vacuolar changes were scored in nine standard gray matter regions to generate lesion profiles, as previously described [39]. For immunohistochemistry, PrP^Sc^ deposits were detected using the hydrated autoclaving method with the anti-PrP monoclonal antibody 31C6 against the epitope corresponding to amino acids 143–149 of murine PrP [46]. Immunoreactions were visualized with the anti-mouse universal immunoperoxidase polymer (Nichirei Histofine Simple Stain MAX-PO (M); Nichirei, Tokyo, Japan) as the secondary antibody and 3,3′-diaminobenzidine tetrahydrochloride as the chromogen.

### Statistical analysis

Statistical analyses were performed to evaluate differences across various experimental parameters. One-way ANOVA followed by Tukey’s test was used to assess statistically significant differences in conversion efficiencies among treatments. Survival curve differences were analyzed using the log-rank test. The glycoform ratios of PrP^Sc^ between groups were compared using the Student’s *t*-test. Vacuolation profiles in brain regions were evaluated using two-way ANOVA with Sidak’s multiple comparison test, considering prion strains and brain regions as variables. For the PK sensitivity assay, a two-way ANOVA with Sidak’s multiple-comparison test was conducted, with PMCA products and PK concentration as variables. All statistical analyses were performed using GraphPad Prism 8.4.3 software (San Diego, CA, USA). Data are presented as means ± standard deviations, and statistical significance was defined as p < 0.05.

## Supporting information

S1 Data Underlying numerical data for all graphs. This Excel workbook contains the underlying numerical data for all graphs presented in the manuscript. Each worksheet corresponds to one figure panel or one experiment. The README worksheet describes the contents of each worksheet, abbreviations, normalization methods, scoring definitions, and other relevant notes.
(XLSX)

S1 Fig Serial non-seeded PMCA using Bo^Tg^/KO, Ov^Tg^, and Ce^Ki^ BH in the presence of digitonin and heparin. Twelve rounds of non-seeded PMCA were performed with Bo^Tg^/KO BH, Ov^Tg^ BH and Ce^Ki^ BH in the presence of digitonin and heparin. No spontaneous PrPres generation was observed. CB, Sc, and CW denote reactions seeded with 0.01% C-BSE, classical scrapie and CWD BH, respectively.
(PDF)

S2 Fig WB banding patterns of CWD PrP^Sc^ from Ce^Ki^ mice inoculated with PMCA products or infected BH. Ten percent medulla oblongata homogenates from mice inoculated with CWD/Ce^Ki^-PMCA products or CWD-infected BH were digested with PK (40 μg/mL) and analyzed by WB. Banding patterns were highly similar between groups.
(PDF)

S3 Fig Glycoform ratios of C-type and C2-type PrPres (A), and CH-type and CH2-type PrPres (B). Relative proportions of diglycosylated (white), monoglycosylated (gray), and unglycosylated (black) PrPres were quantified using the T2 antibody. Data are mean ± standard deviation from three independent experiments. Glycoform ratios were compared among sample sets (A: C-type #2314/Sh, C2-type #2314/Sh, and C2-type G3571/Mo; B: CH- and CH2-type samples from #294/Sh and #294/Mo#1 and #2) using two-way ANOVA with Sidak’s multiple-comparison test. Although trends were observed, no statistically significant differences were detected (all adjusted p > 0.9999).
(PDF)

S4 Fig Expanded non-seeded PMCA controls using Ov^Tg^ BH. (A) Twelve independent non-seeded PMCA reactions were performed using Ov^Tg^ BH as the substrate under digitonin/heparin-supplemented conditions in the absence [DH(-A)] or presence of AE (DHA). Reactions were serially propagated for 12 PMCA rounds, and round-12 (R12) products were analyzed by Western blotting after PK digestion. Numbers above the lanes indicate independent reaction numbers. (B) To assess whether the faint band-like signals observed in panel A represented propagating PrPres, R12 products from DH(-A) lanes 4, 5, and 7 and DHA lane 9 were subjected to one additional PMCA round (R13). The signals detected in panel A were not further amplified after R13, indicating that they represented nonspecific band-like signals rather than spontaneous PrPres formation. No spontaneous PrPres formation was detected under either condition. Molecular weight markers were Bio-Rad Precision Plus Protein Dual Color Standards (#1610374), and molecular weights are shown in kDa.
(PDF)

S5 Fig Comparison of DH(-A) and DHA PMCA products seeded with BHs from Ov^Tg^ mice inoculated with scrapie isolates. Twelve rounds of DH(-A) and DHA PMCA were performed using BHs from Ov^Tg^ mice inoculated with G3571, #2314 or #294 isolates. Two mice per isolate were used as seeds. WB analysis of products from rounds 3, 6 and 12 is shown. Round 12 products were also used in Fig 5B–D.
(PDF)

S1 Raw images Original uncropped blot and gel images supporting the main and supporting figures. The file includes original chemiluminescence images and contrast-enhanced images used to visualize molecular weight markers, where available. Lane labels, molecular weight markers, and lanes not used in the final figures are indicated.
(PDF)
