# Supplementary material for: Evidence for multiple prion conformers in natural scrapie isolates
Source: PLoS Pathog. 2026 Sep 17;22(9):e1014283. doi: 10.1371/journal.ppat.1014283 (PMC13596833; doi:10.1371/journal.ppat.1014283)
Supplement: Supplementary file 1 [file ppat.1014283.s002.pdf]

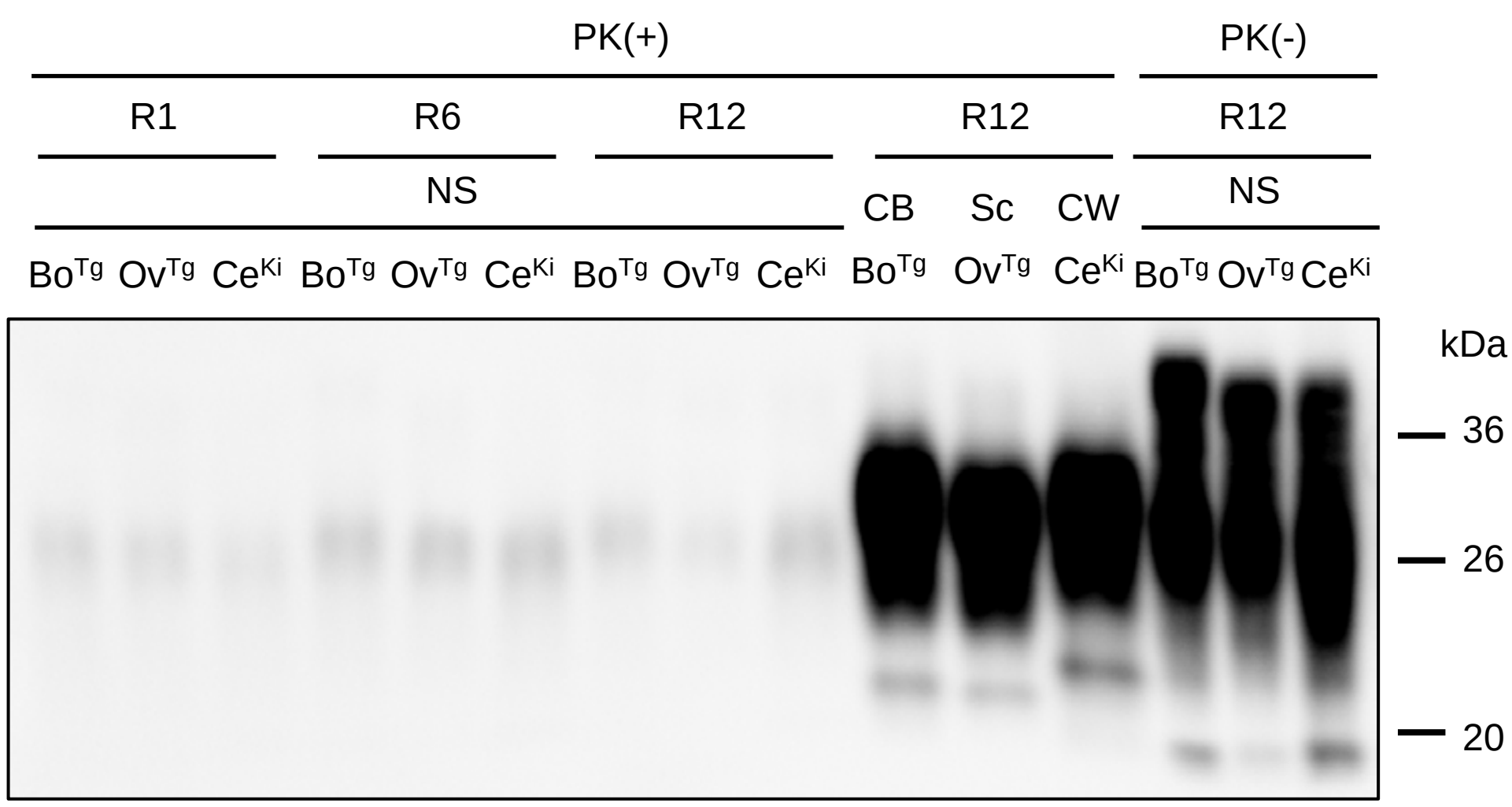
PK(+)
PK(-)
R1
R6
R12
R12
R12
NS
CB
Sc
CW
NS
Bo^{Tg} Ov^{Tg} Ce^{Ki} Bo^{Tg} Ov^{Tg} Ce^{Ki} Bo^{Tg} Ov^{Tg} Ce^{Ki} Bo^{Tg} Ov^{Tg} Ce^{Ki} Bo^{Tg} Ov^{Tg} Ce^{Ki}
kDa
36
26
20
