# Supplementary material for: Evidence for multiple prion conformers in natural scrapie isolates
Source: PLoS Pathog. 2026 Sep 17;22(9):e1014283. doi: 10.1371/journal.ppat.1014283 (PMC13596833; doi:10.1371/journal.ppat.1014283)

CWD/Ce^{Ki}-
PMCA

CWD
BH

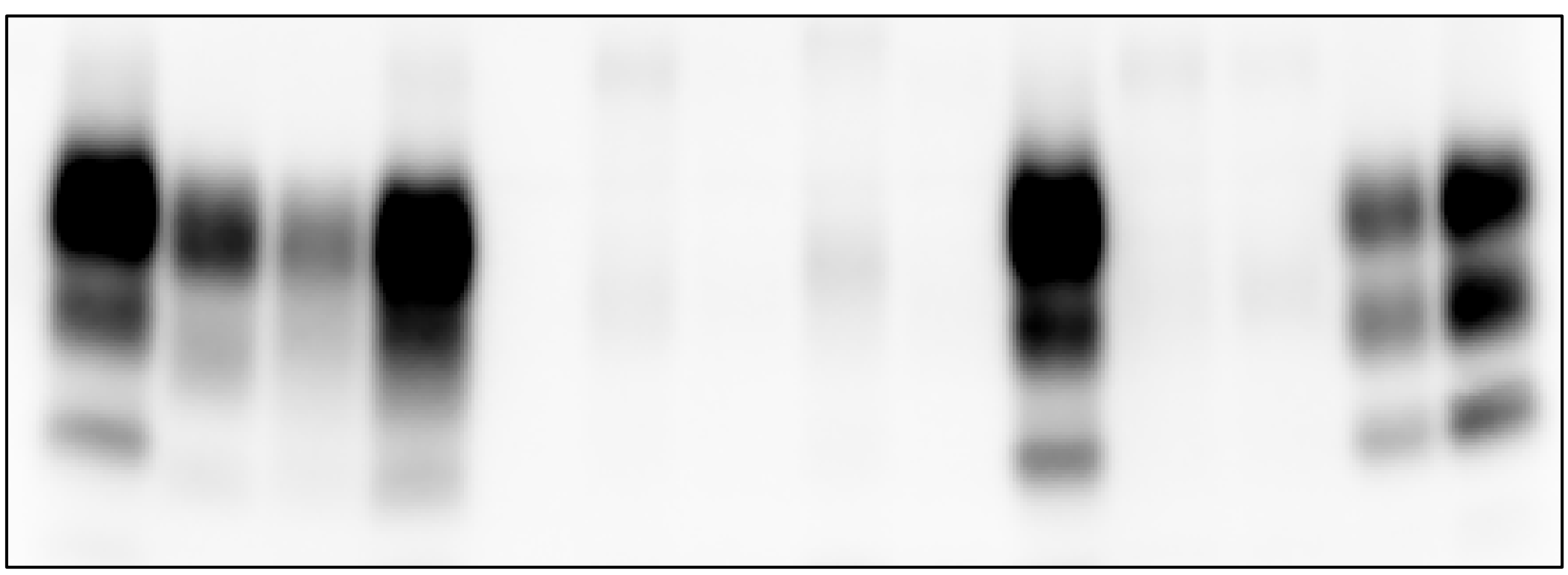

Supplement: Supplementary file 2 [file ppat.1014283.s003.pdf]
