# Supplementary material for: Evidence for multiple prion conformers in natural scrapie isolates
Source: PLoS Pathog. 2026 Sep 17;22(9):e1014283. doi: 10.1371/journal.ppat.1014283 (PMC13596833; doi:10.1371/journal.ppat.1014283)
Supplement: Supplementary file 3 [file ppat.1014283.s004.pdf]

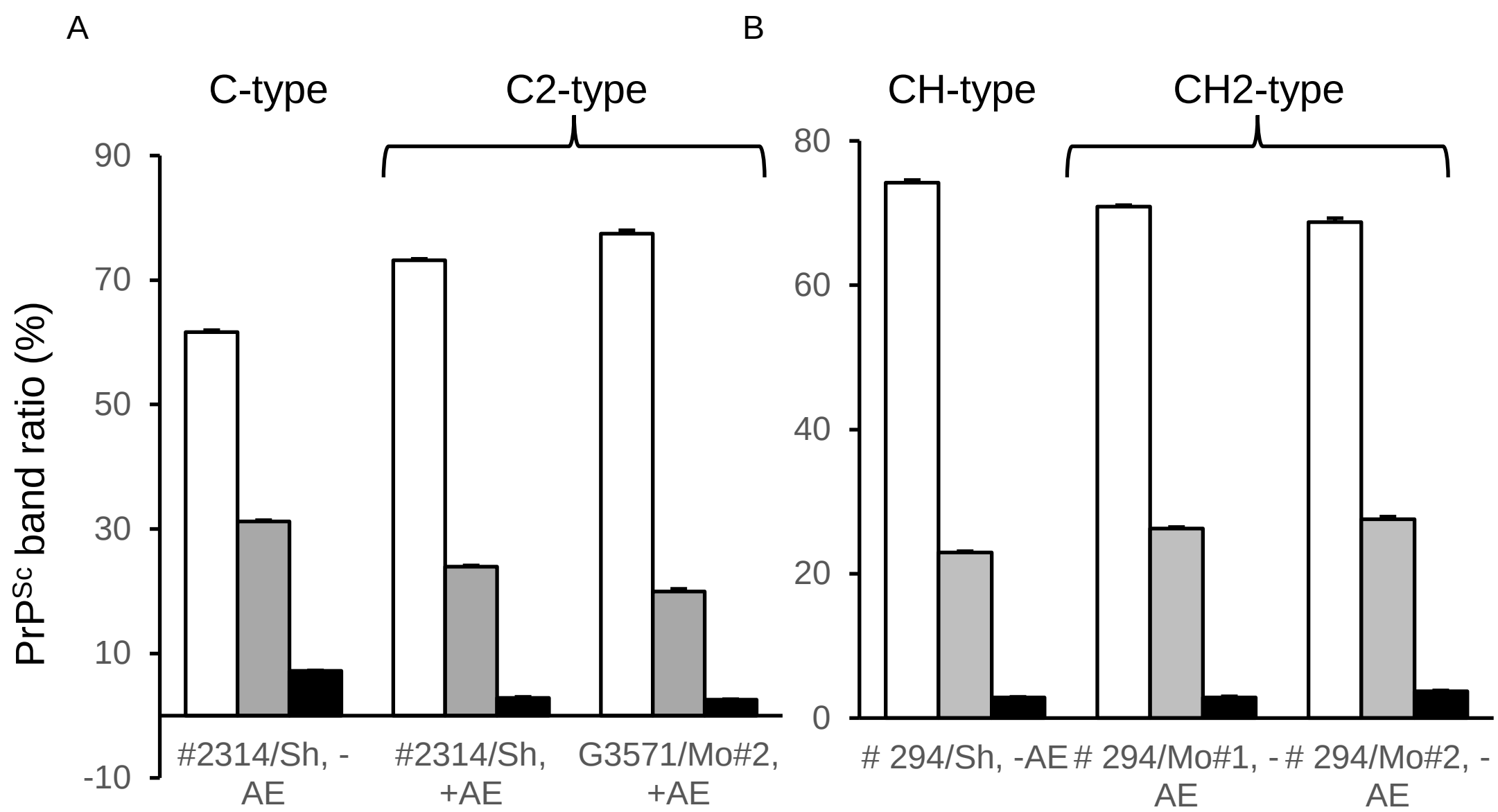
A
B
C-type
C2-type
CH-type
CH2-type
PrPSc band ratio (%)
90
70
50
30
10
-10
80
60
40
20
0
#2314/Sh, -AE
#2314/Sh, +AE
G3571/Mo#2, +AE
294/Sh, -AE
294/Mo#1, -AE
294/Mo#2, -AE
