# Supplementary material for: Evidence for multiple prion conformers in natural scrapie isolates
Source: PLoS Pathog. 2026 Sep 17;22(9):e1014283. doi: 10.1371/journal.ppat.1014283 (PMC13596833; doi:10.1371/journal.ppat.1014283)
Supplement: Supplementary file 4 [file ppat.1014283.s005.pdf]

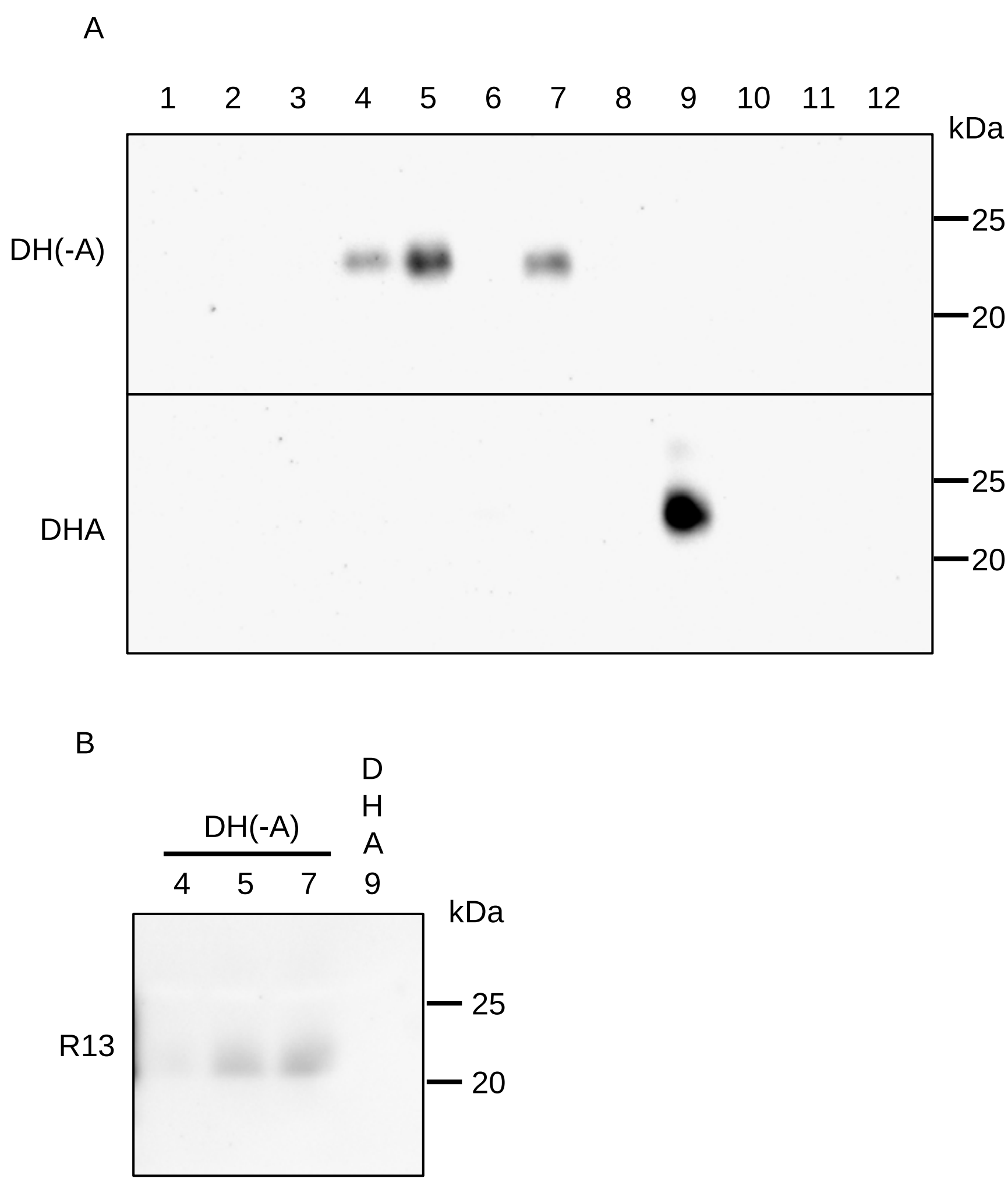
A
1 2 3 4 5 6 7 8 9 10 11 12
kDa
DH(-A)
DHA
25
20
25
20
B
DH(-A)
DHA
4 5 7 9
kDa
R13
25
20
