# Supplementary material for: Evidence for multiple prion conformers in natural scrapie isolates
Source: PLoS Pathog. 2026 Sep 17;22(9):e1014283. doi: 10.1371/journal.ppat.1014283 (PMC13596833; doi:10.1371/journal.ppat.1014283)
Supplement: Supplementary file 5 [file ppat.1014283.s006.pdf]

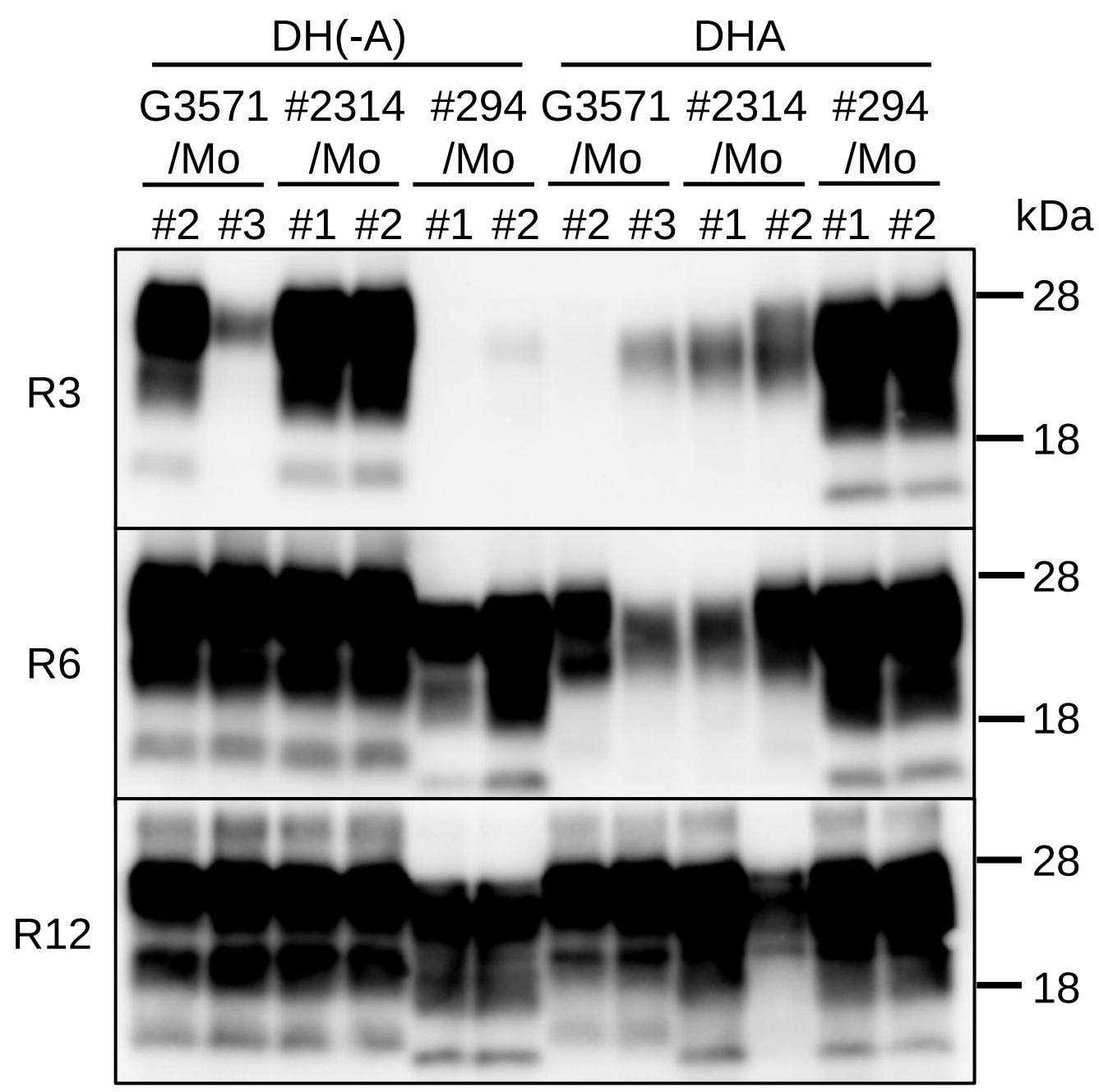
DH(-A)
DHA
G3571 /Mo
#2314 /Mo
#294 /Mo
G3571 /Mo
#2314 /Mo
#294 /Mo
#2 #3 #1 #2 #1 #2 #2 #3 #1 #2 #1 #2
kDa
R3
R6
R12
28
18
28
18
28
18
