# Supplementary material for: Evidence for multiple prion conformers in natural scrapie isolates
Source: PLoS Pathog. 2026 Sep 17;22(9):e1014283. doi: 10.1371/journal.ppat.1014283 (PMC13596833; doi:10.1371/journal.ppat.1014283)

Fig. 1A. Original uncropped chemiluminescence image.
The molecular weight marker lanes (M) are indicated, although the marker bands are not visible in this chemiluminescence exposure.
The lane marked with X was not used in the final figure.

M ICR BoTg CeKi OvTg X M

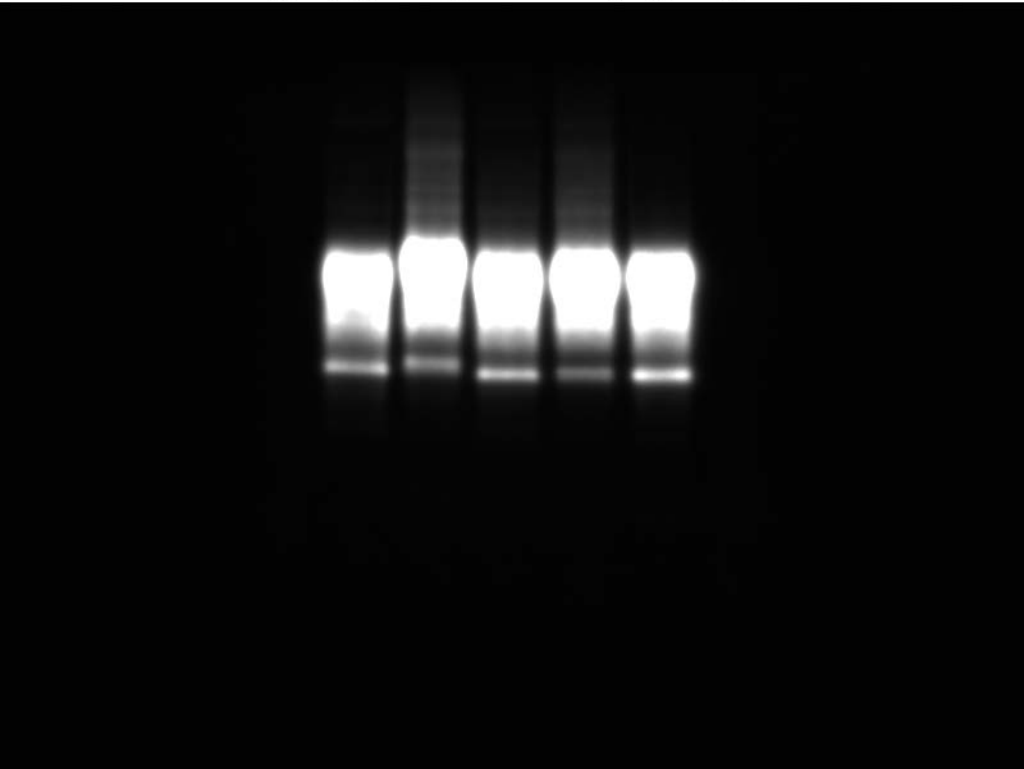

Fig. 1A. Contrast-enhanced image of the same membrane.
This image is shown only to visualize the molecular weight marker lanes (M) and was not used for band interpretation.

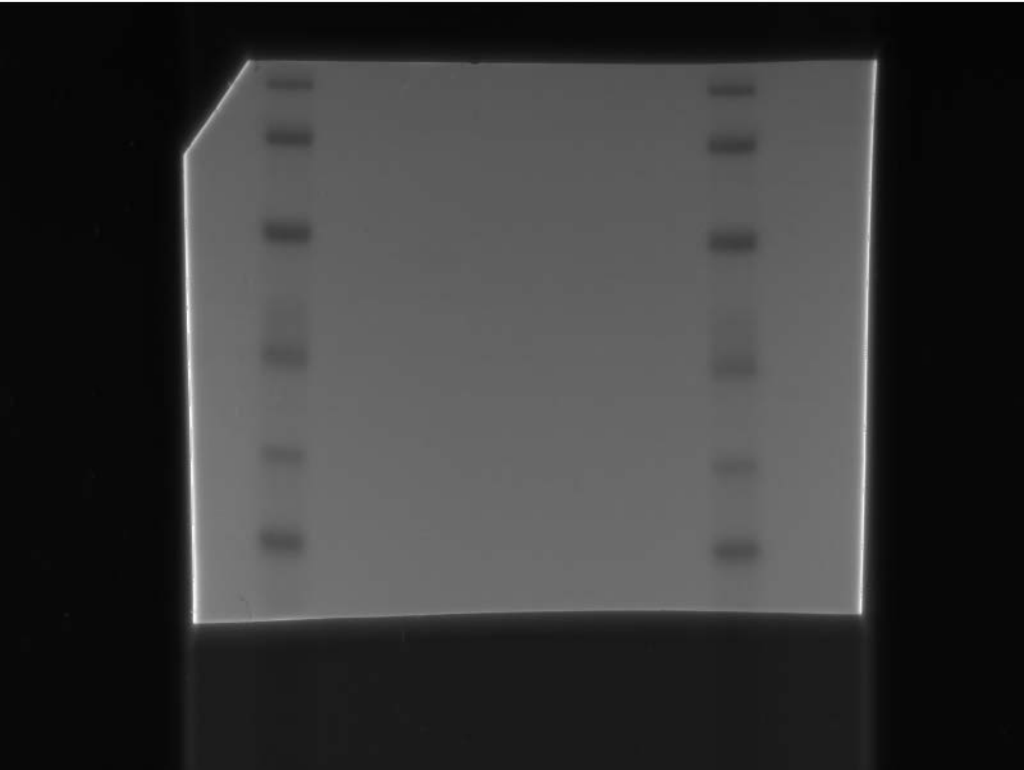

Fig. 1C, left panel. Original uncropped chemiluminescence image.
Three gels were transferred onto a single PVDF membrane and detected simultaneously.
No molecular weight marker image was captured for this blot.
Lanes marked with X were not used in the final figure.

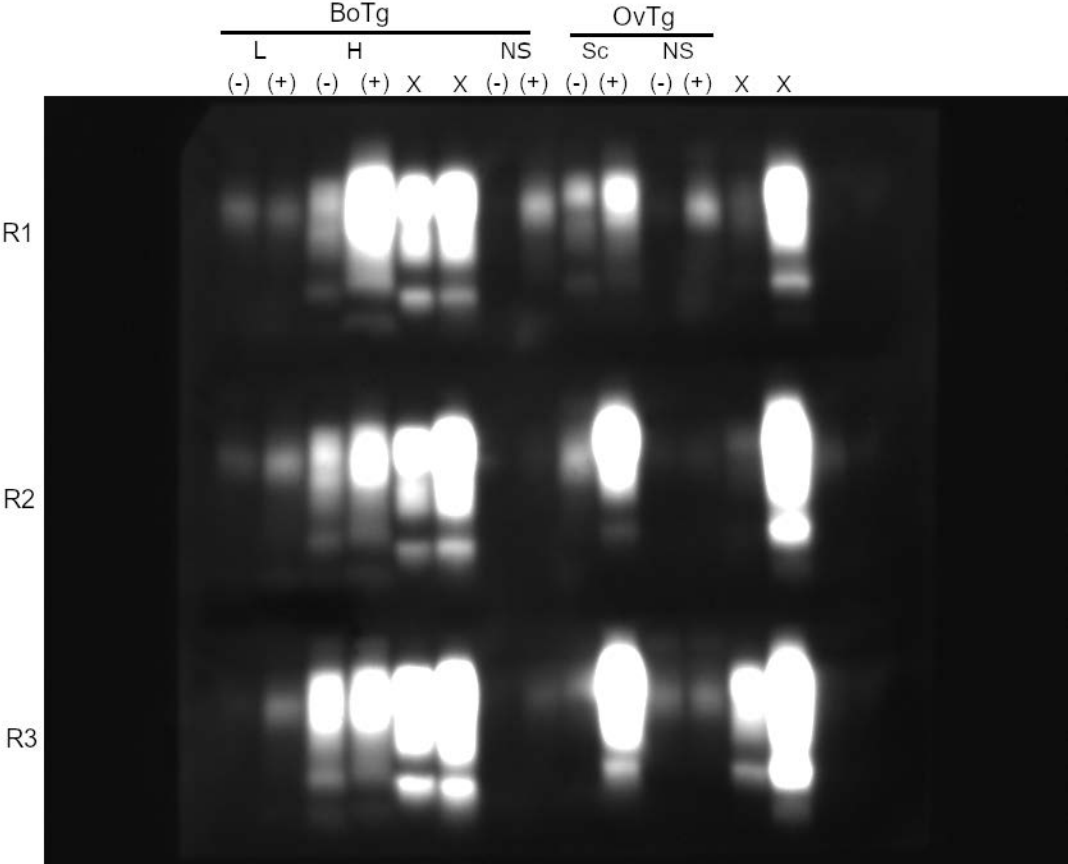

Fig. 1C, right panel. Original uncropped chemiluminescence image.
Three gels were transferred onto a single PVDF membrane and detected simultaneously.
No molecular weight marker image was captured for this blot.
Only the bottom gel image was used for the 22L result in Fig. 1C, right panel; the upper two gel images were not used in the final figure.
Lanes marked with X were not used in the final figure.

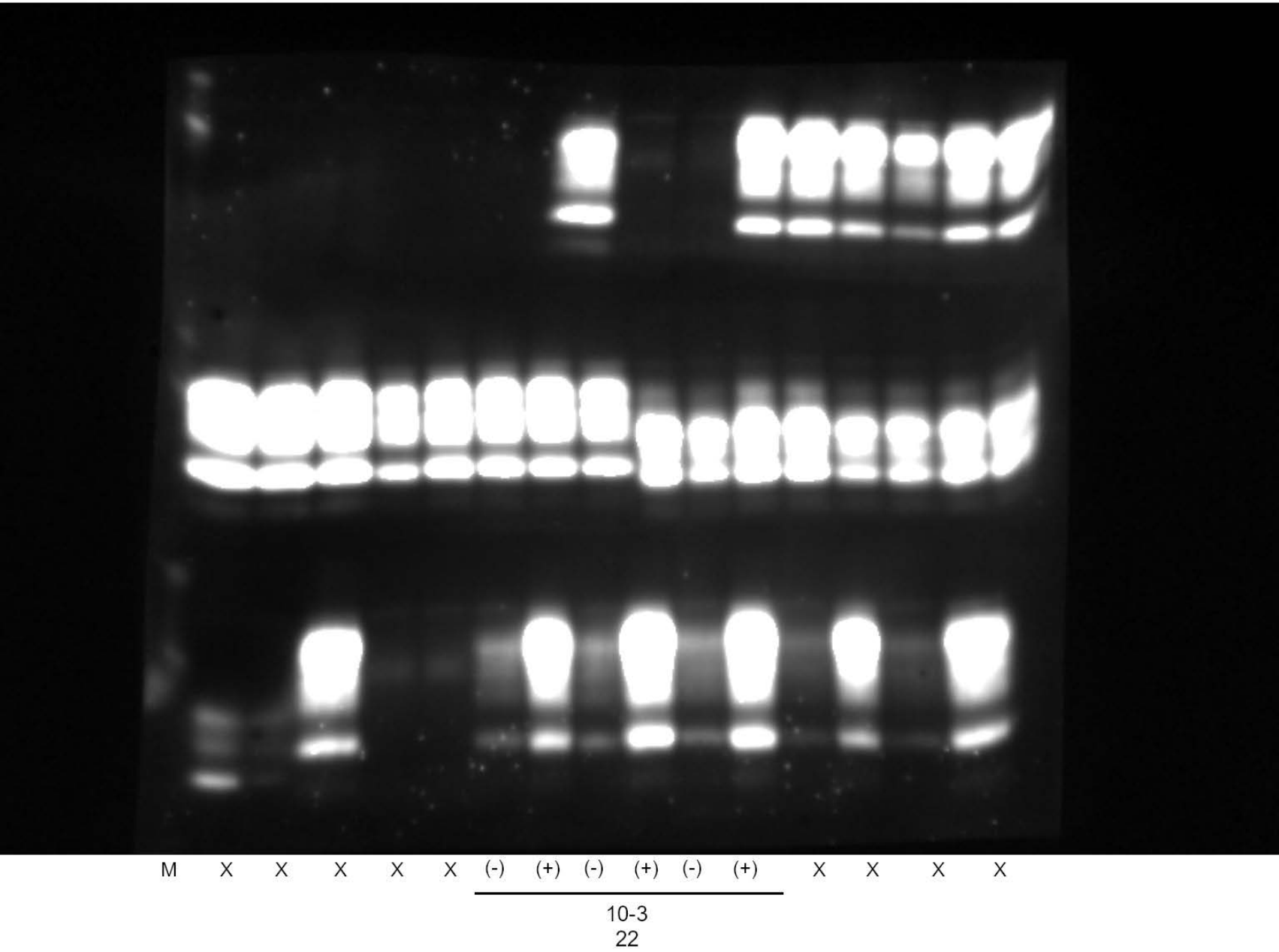

Fig. 1C, right panel. Original uncropped chemiluminescence image.
Three gels were transferred onto a single PVDF membrane and detected simultaneously.
The molecular weight marker lane (M) is indicated; however, a separate molecular weight marker image was not captured for this blot.
Lanes marked with X were not used in the final figure.

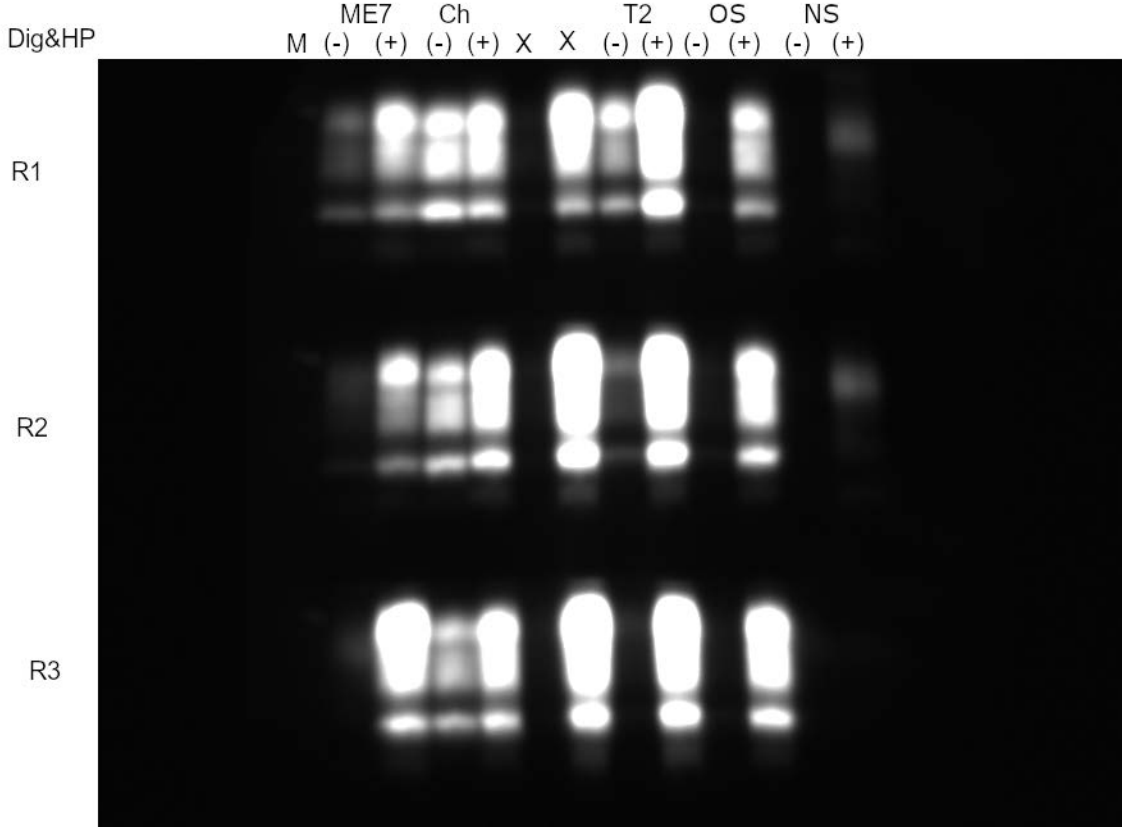

Fig. 1D, C-BSE panel. Original uncropped chemiluminescence image.
Three gels were transferred onto a single PVDF membrane and detected simultaneously.
Because the R1/R2 lanes start with the 10-5 dilution and the R3 lanes start with the 10-7 dilution, lane labels are shown separately for R1/R2 and R3.
The molecular weight marker lane (M) is indicated; however, a separate molecular weight marker image was not captured for this blot.

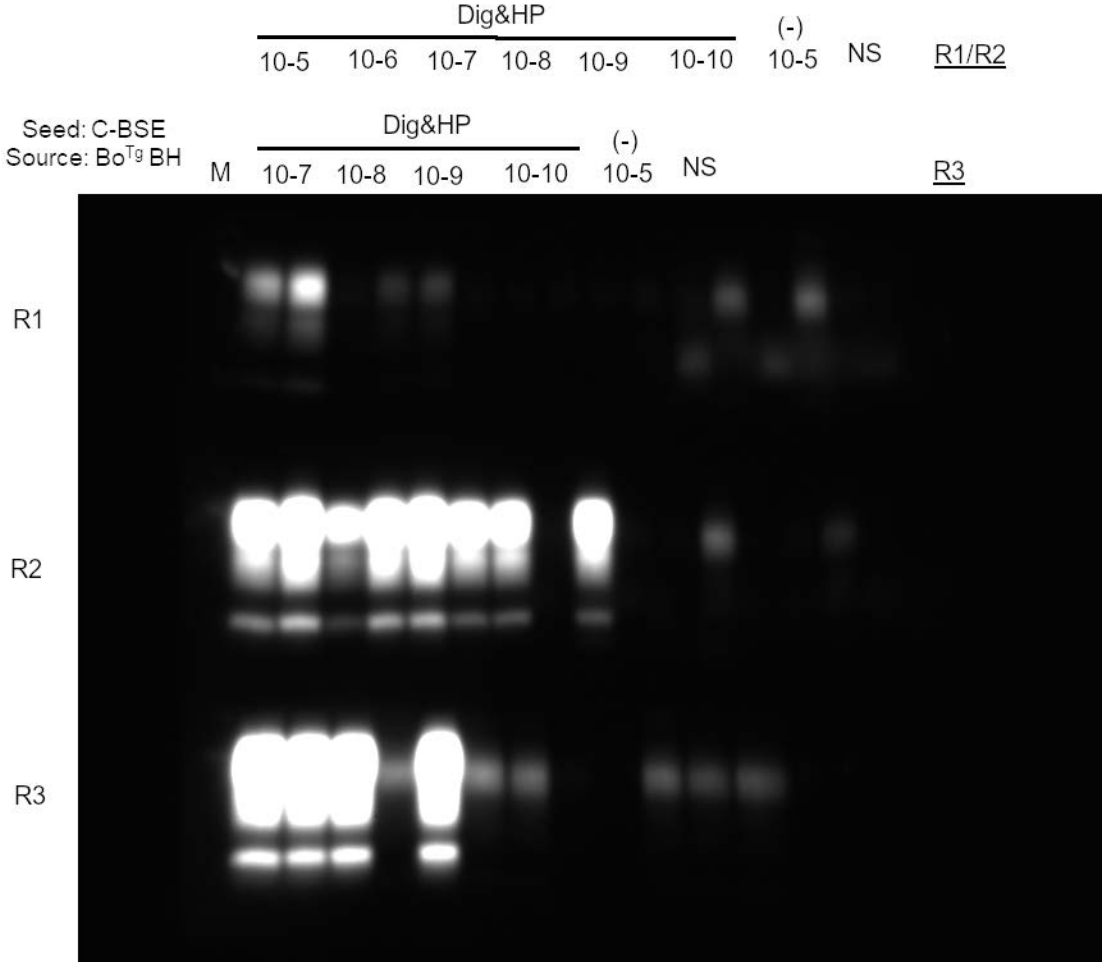

Fig. 1D, C-BSE panel. Original uncropped chemiluminescence image.
Three gels were transferred onto a single PVDF membrane and detected simultaneously.
This image shows the R4–R6 results.
The molecular weight marker lane (M) is indicated; however, a separate molecular weight marker image was not captured for this blot.

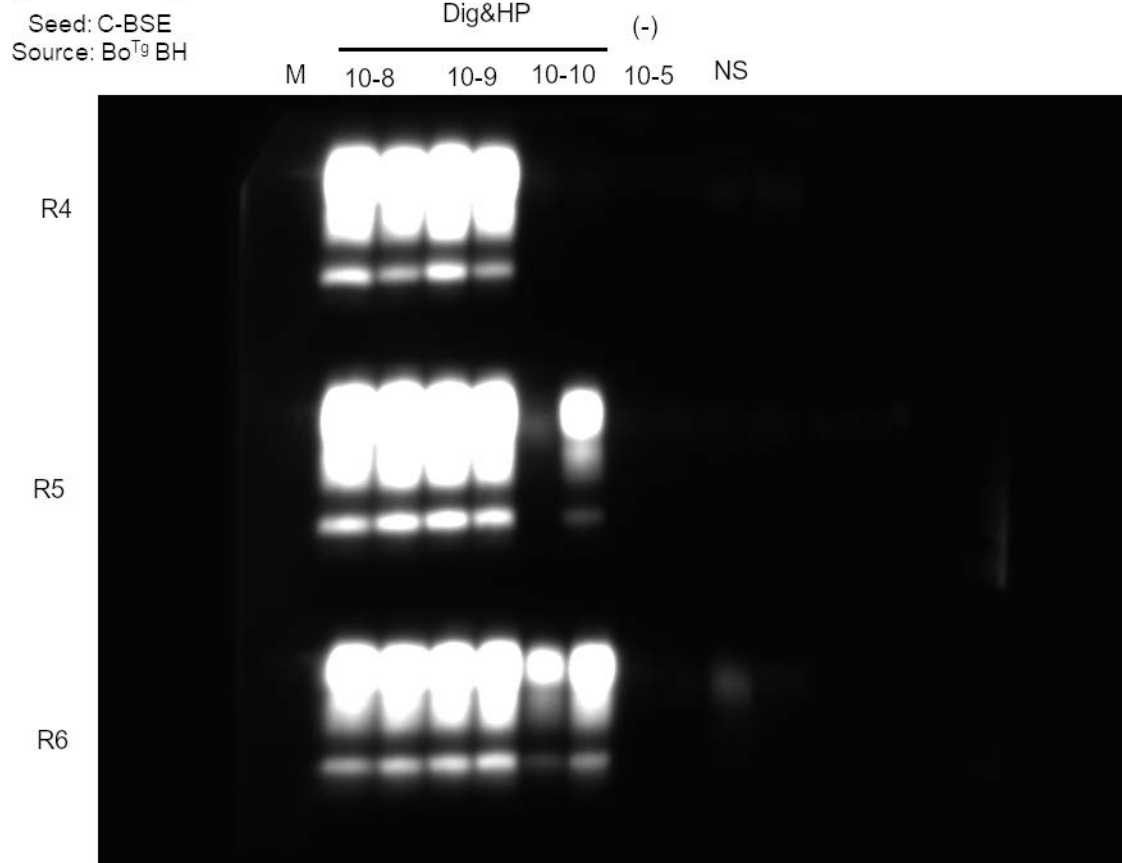

Fig. 1D, CWD panel. Original uncropped chemiluminescence image.
Three gels were transferred onto a single PVDF membrane and detected simultaneously.
This image shows the R1–R3 results.
Because the R1/R2 lanes start with the 10^-5 dilution and the R3 lanes start with the 10^-7 dilultion, lane labels are shown separately for R1/R2 and R3.
The molecular weight marker lane (M) is indicated; however, a separate molecular weight marker image was not captured for this blot.

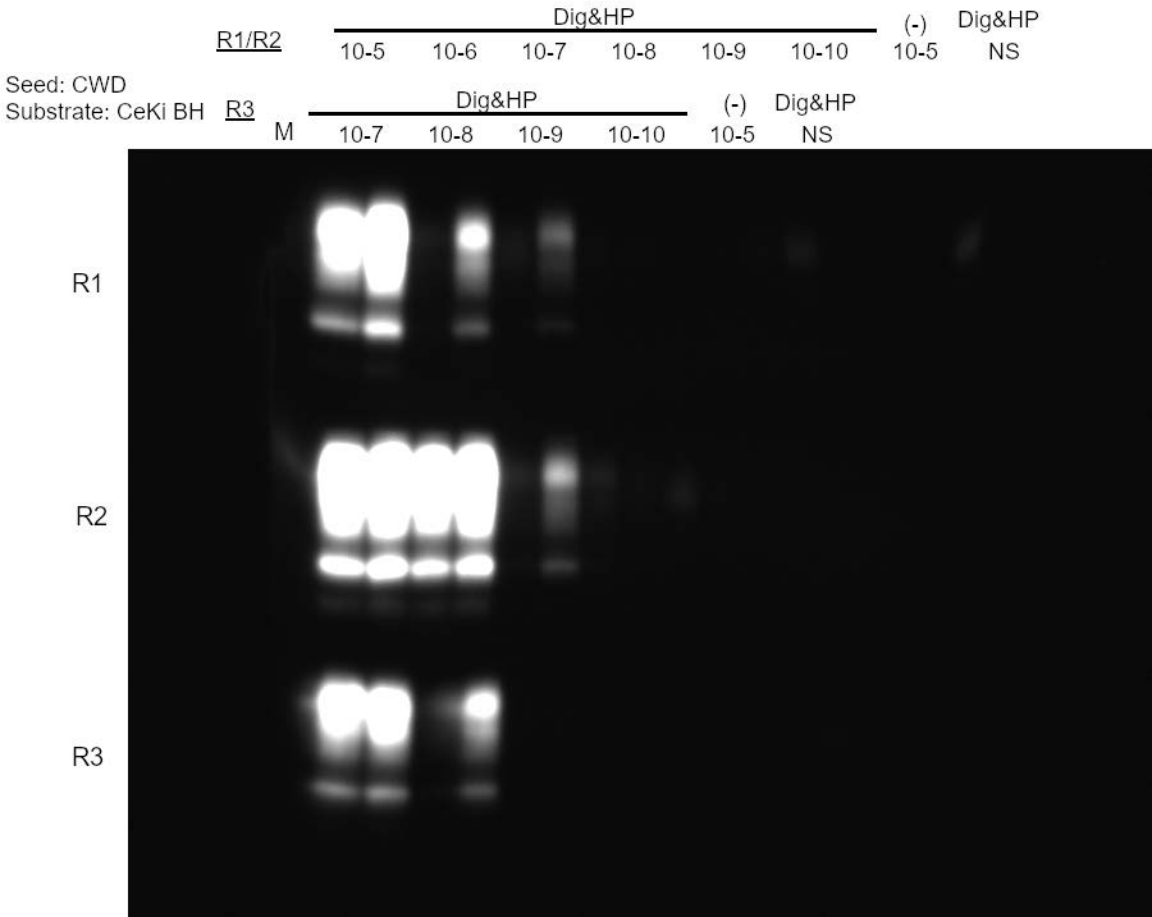

Fig. 1D, CWD panel. Original uncropped chemiluminescence image.
Three gels were transferred onto a single PVDF membrane and detected simultaneously.
This image shows the R4–R6 results.
The molecular weight marker lane (M) is indicated; however, a separate molecular weight marker image was not captured for this blot.

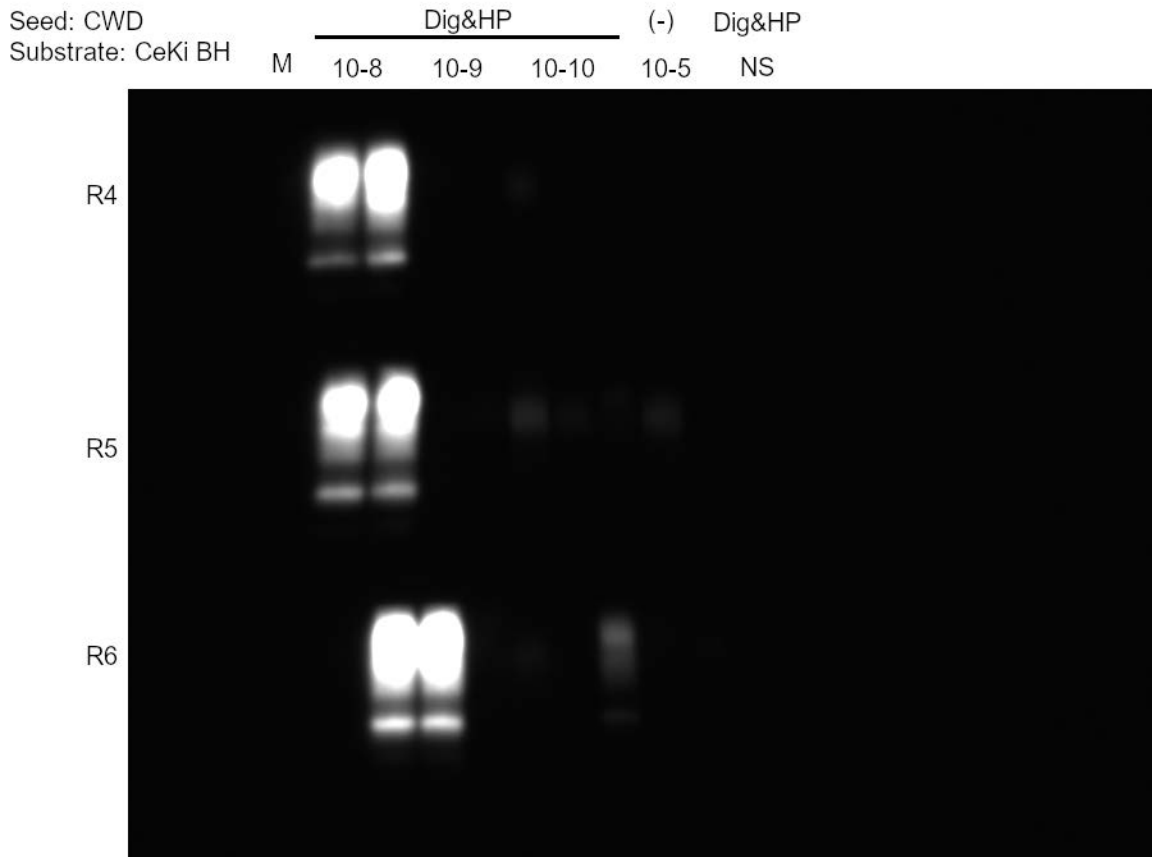

Fig. 1D, scrapie panel. Original uncropped chemiluminescence image.
Three gels were transferred onto a single PVDF membrane and detected simultaneously.
This image shows the R1–R3 results.
Because the R1/R2 lanes start with the 10^-5 dilution and the R3 lanes start with the 10^-8 dilultion, lane labels are shown separately for R1/R2 and R3.
The molecular weight marker lane (M) is indicated; however, a separate molecular weight marker image was not captured for this blot.

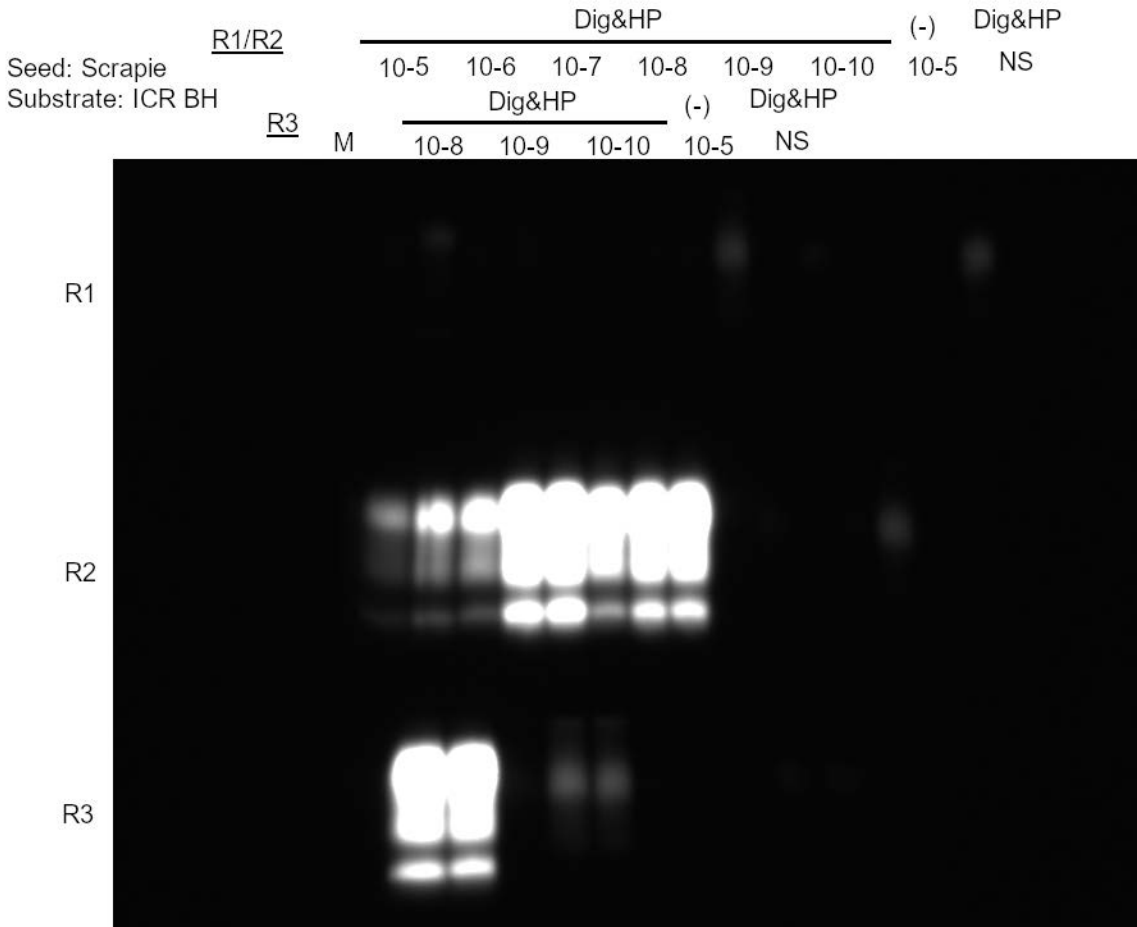

Fig. 1D, scrapie panel. Original uncropped chemiluminescence image.
Three gels were transferred onto a single PVDF membrane and detected simultaneously.
This image shows the R4–R6 results.
The molecular weight marker lane (M) is indicated; however, a separate molecular weight marker image was not captured for this blot.

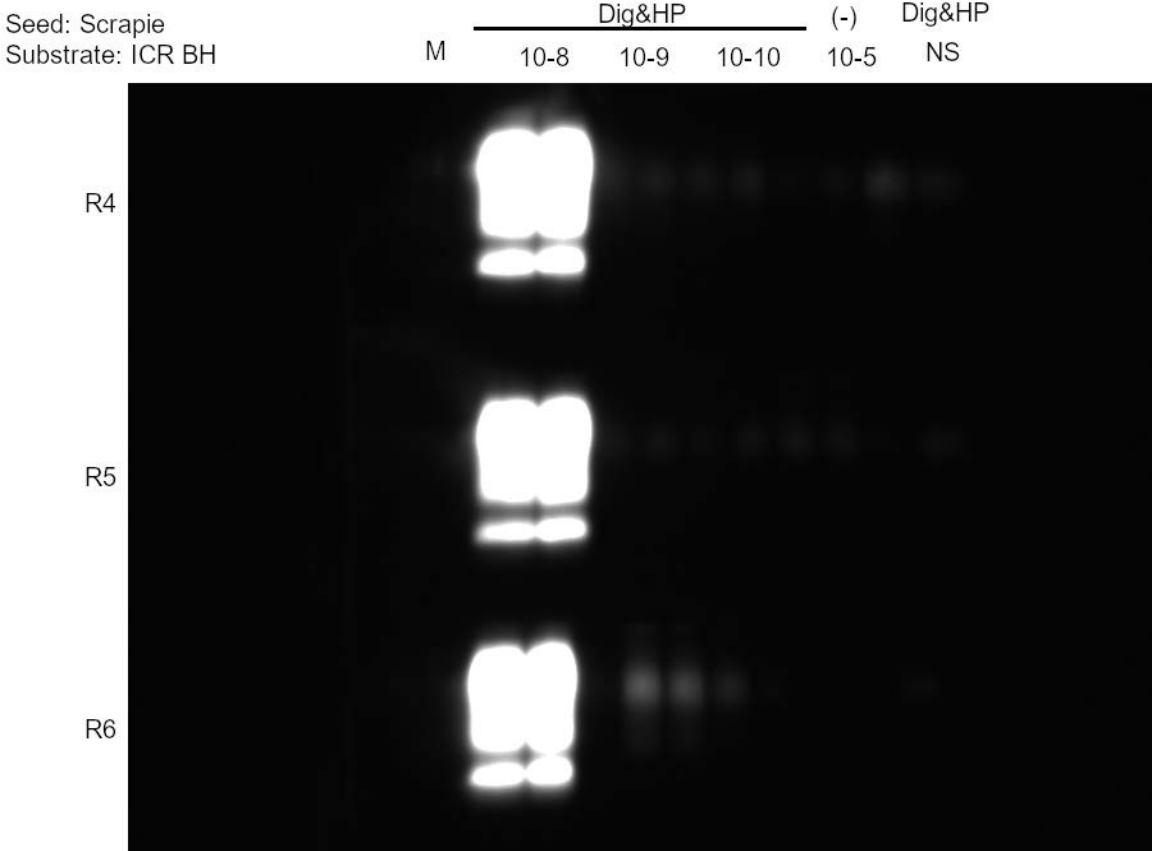

Fig. 2D, left panel. Available uncropped shading-corrected source image.
This image was uniformly shading-corrected to compensate for uneven illumination. The uncorrected original image is no longer available.
The corresponding region was used for Fig. 2D, left panel.

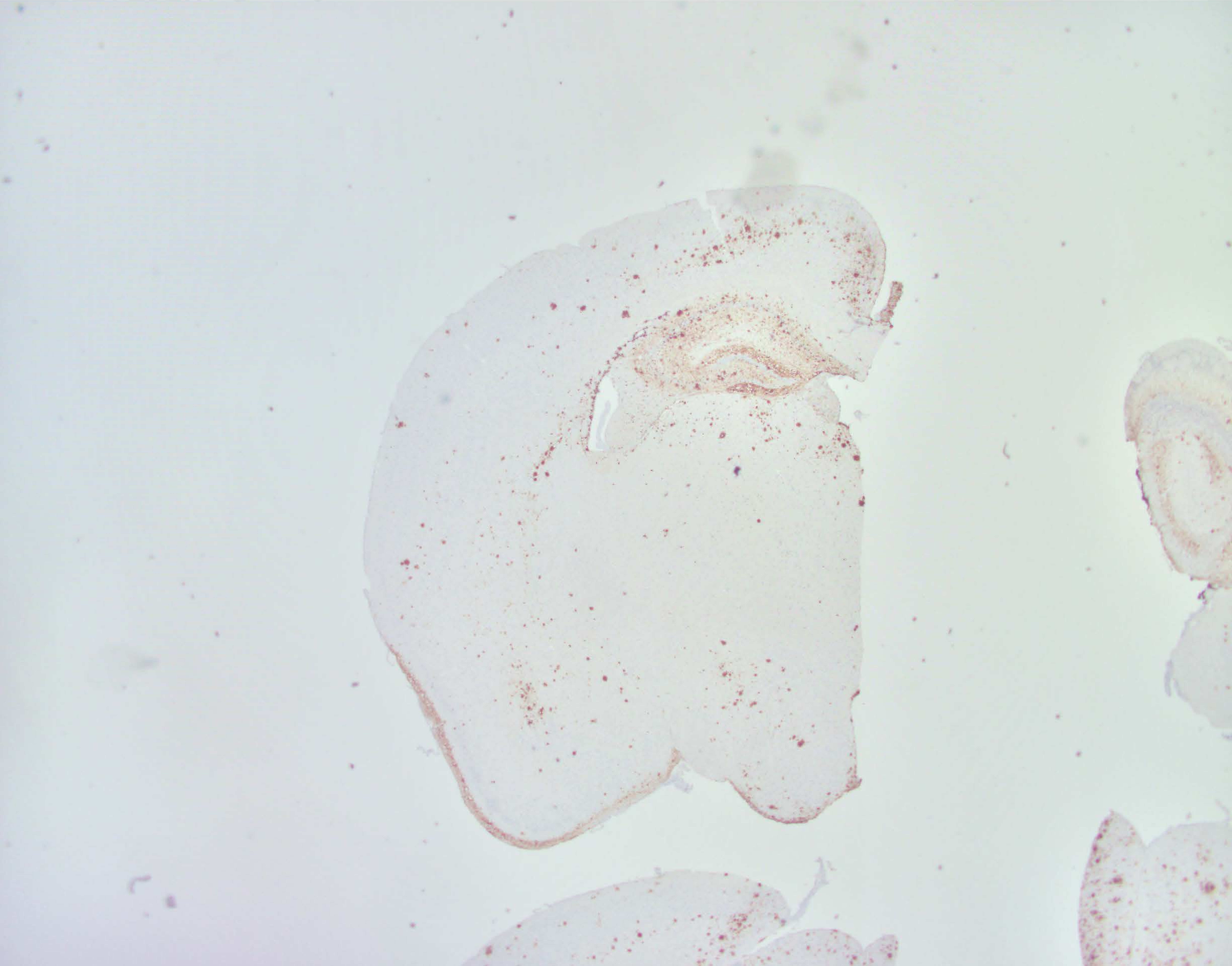

Fig. 2D, right panel. Available uncropped shading-corrected source image.
This image was uniformly shading-corrected to compensate for uneven illumination. The uncorrected original image is no longer available.
The corresponding region was used for Fig. 2D, right panel.

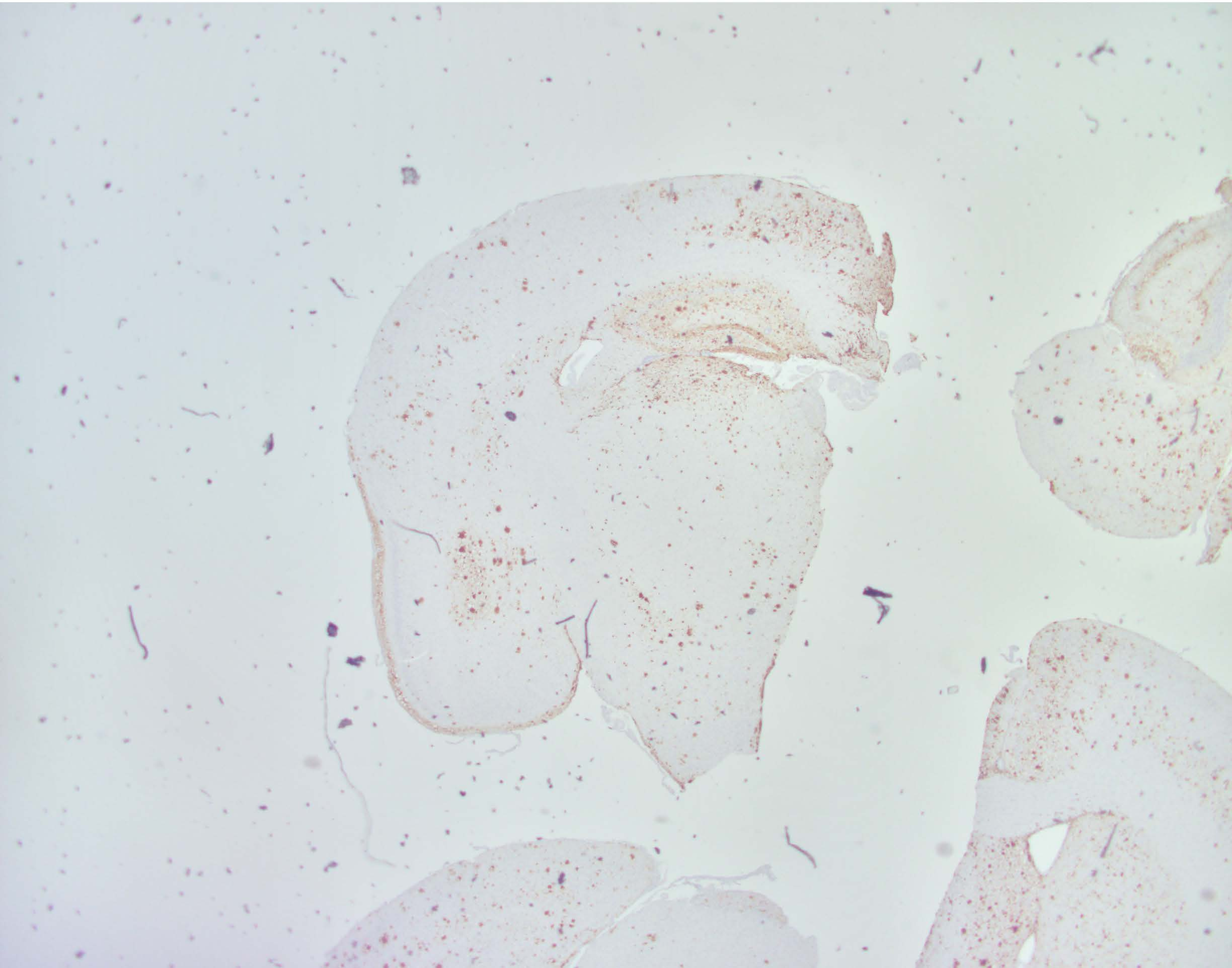

Fig. 2E, lower-left panel. Available uncropped, shading-corrected source image.
This image was uniformly shading-corrected to compensate for uneven illumination before figure preparation. The uncorrected original image was not retained.
The corresponding region was used for Fig. 2E, lower-left panel.

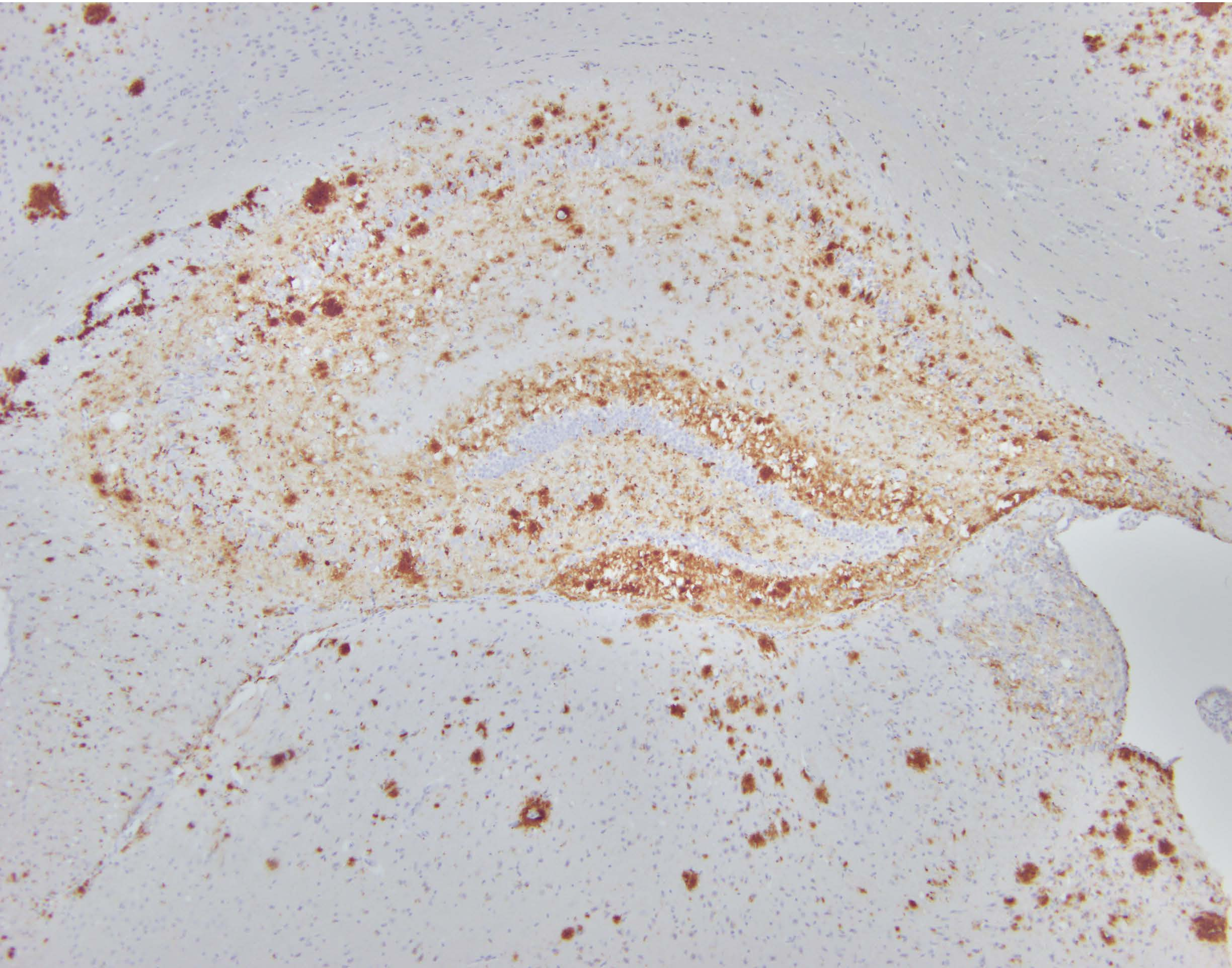

Fig. 2E, lower-right panel. Available uncropped, shading-corrected source image.
This image was uniformly shading-corrected to compensate for uneven illumination before figure preparation. The uncorrected original image was not retained.
The corresponding region was used for Fig. 2E, lower-right panel.

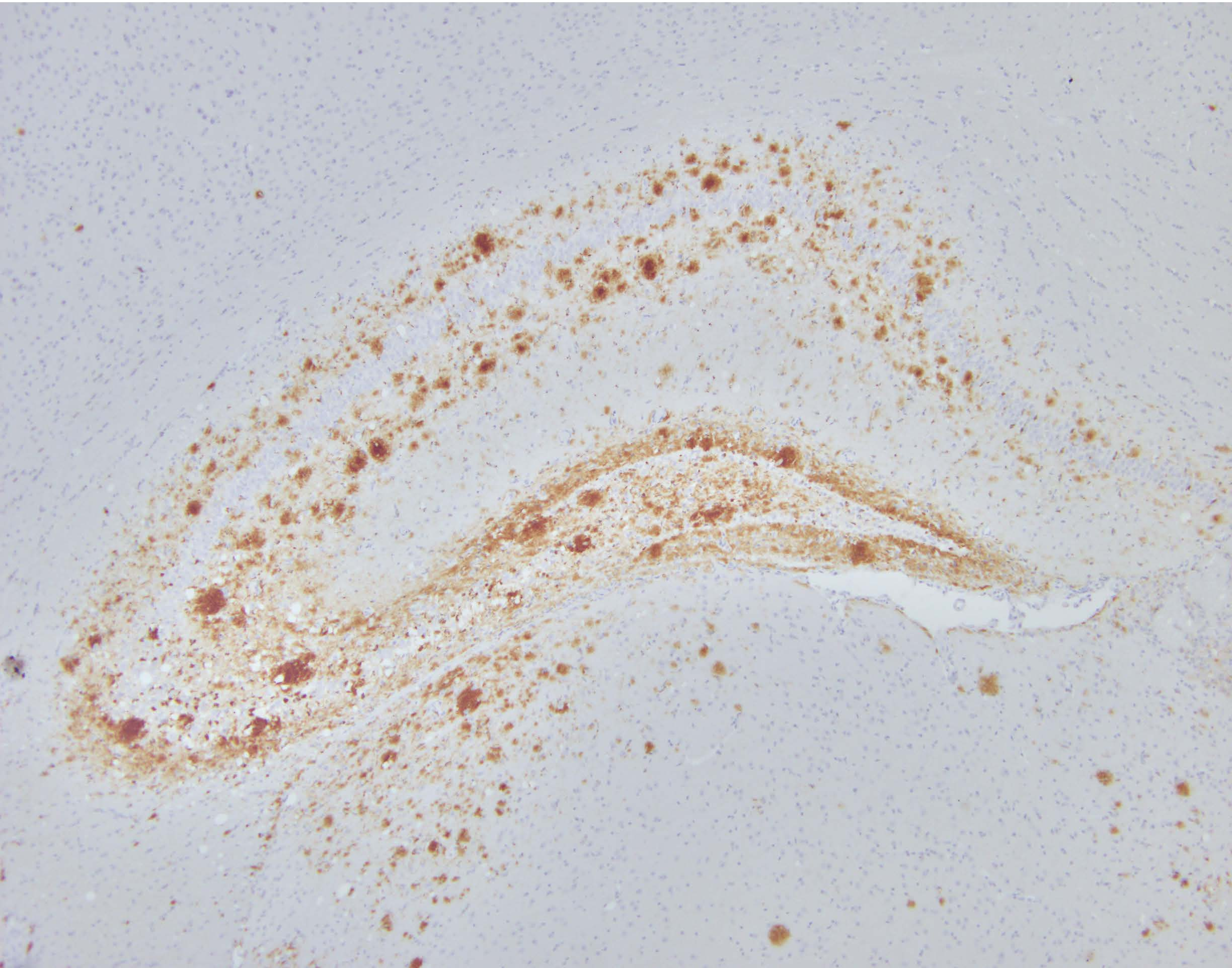

Fig. 2E, upper-left panel. Available uncropped, shading-corrected source image.
This image was uniformly shading-corrected to compensate for uneven illumination before figure preparation. The uncorrected original image was not retained.
The corresponding region was used for Fig. 2E, upper-left panel.

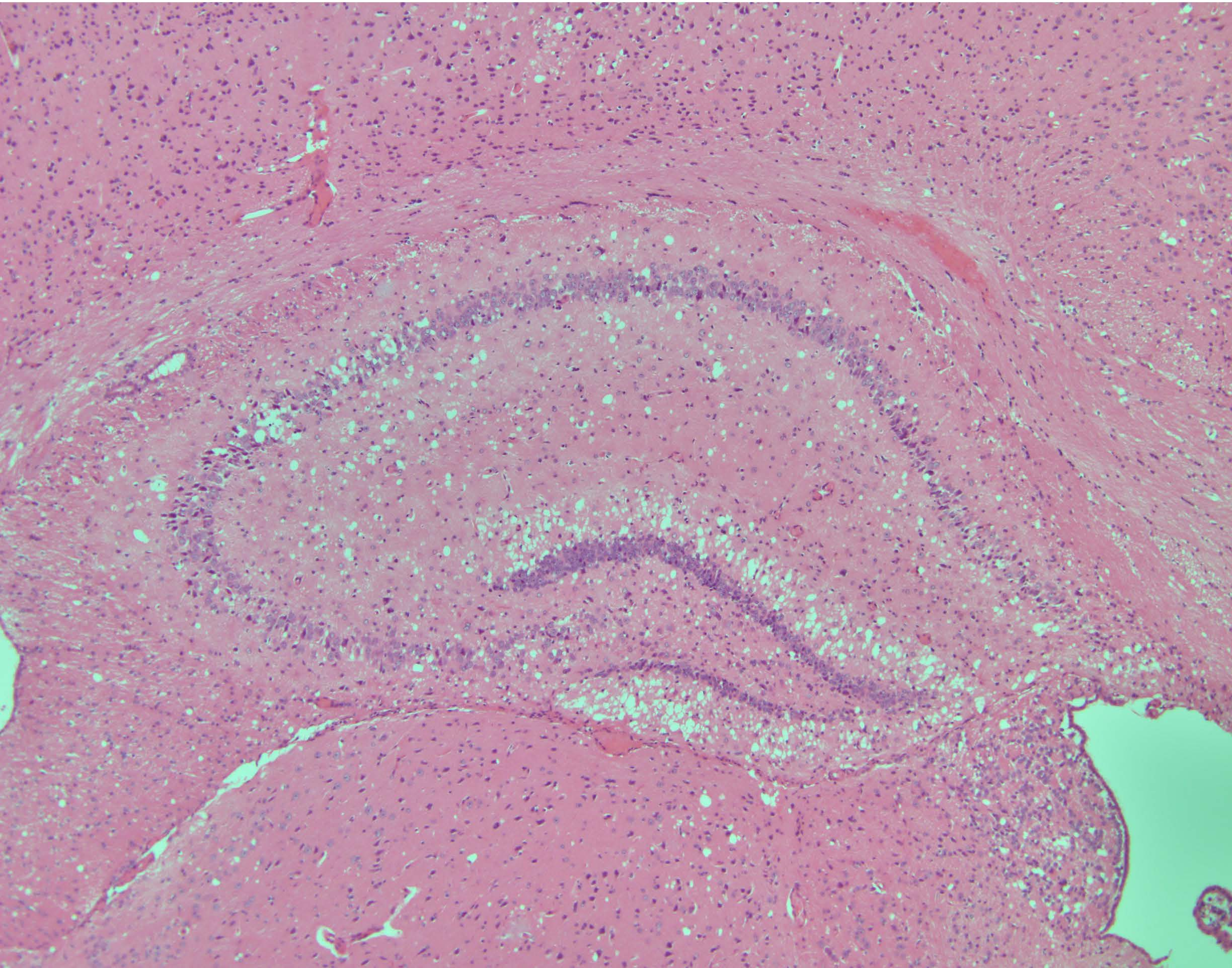

Fig. 2E, upper-right panel. Available uncropped, shading-corrected source image.
This image was uniformly shading-corrected to compensate for uneven illumination before figure preparation. The uncorrected original image was not retained.
The corresponding region was used for Fig. 2E, upper-right panel.

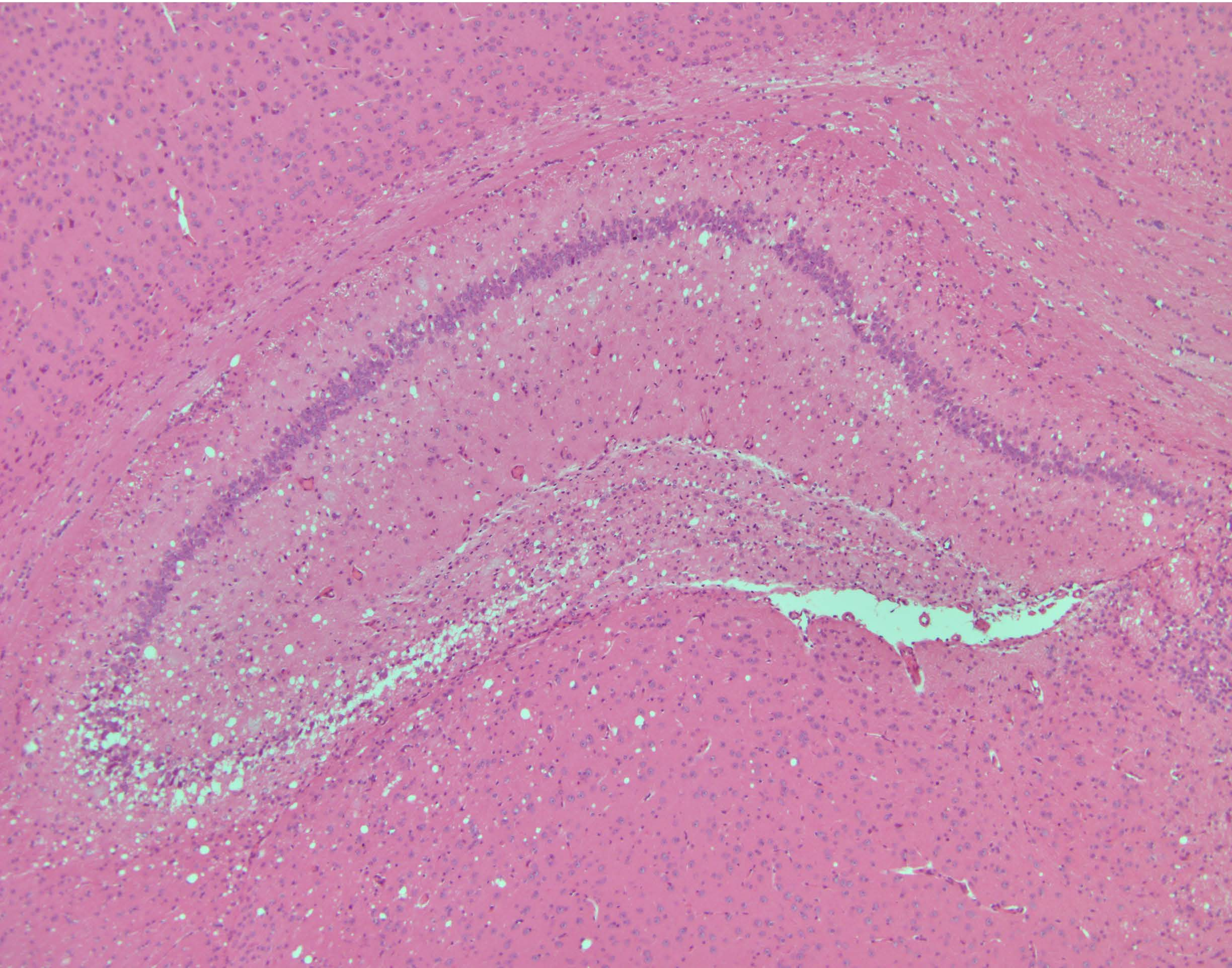

Fig. 3A. Original uncropped chemiluminescence image.
Three gels were transferred onto a single PVDF membrane and detected simultaneously.
This image shows the R1–R3 results.
The molecular weight marker lane is indicated by M on the left.

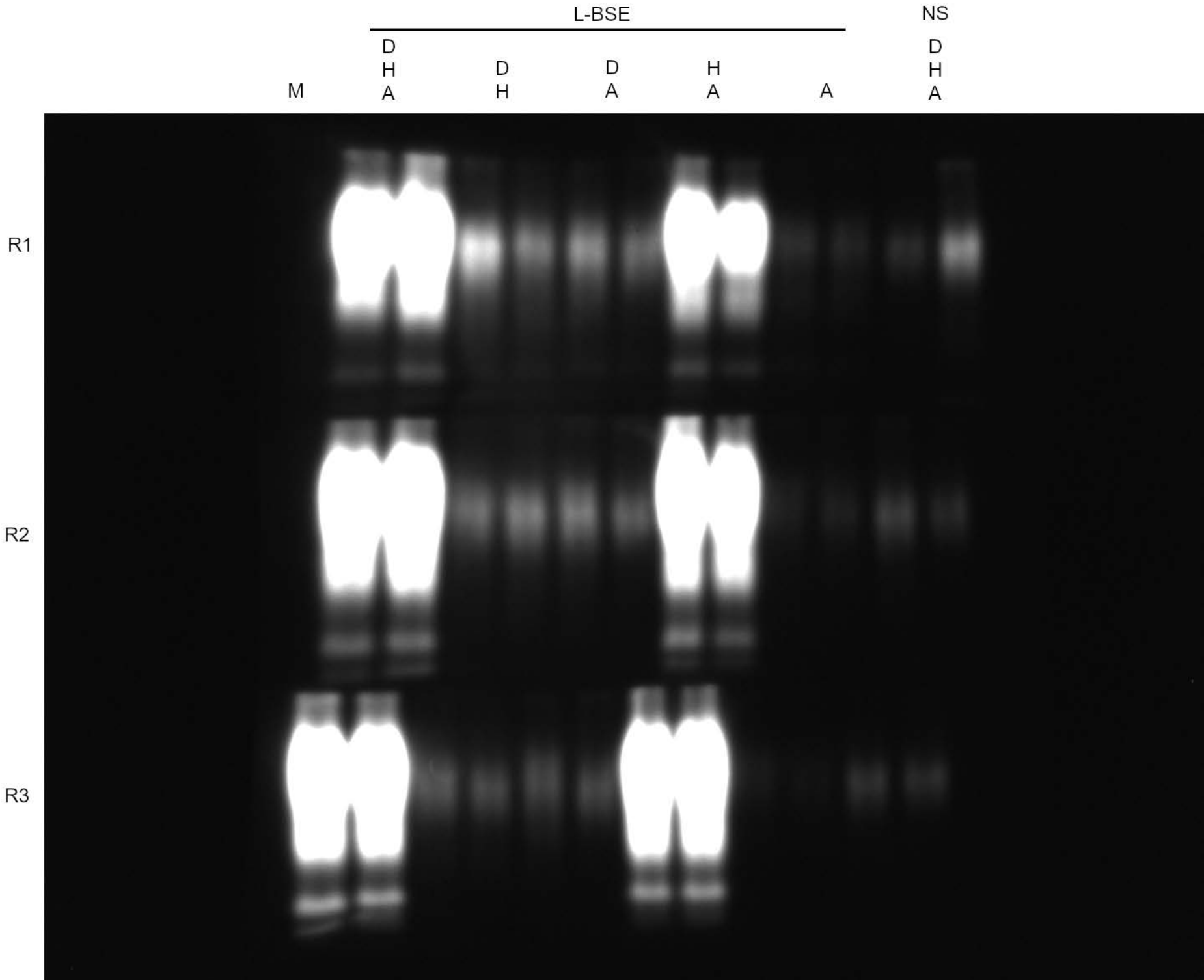

Fig. 3A. Contrast-enhanced image of the same membrane.
This image is shown only to visualize the molecular weight marker lane (M) and was not used for band interpretation.

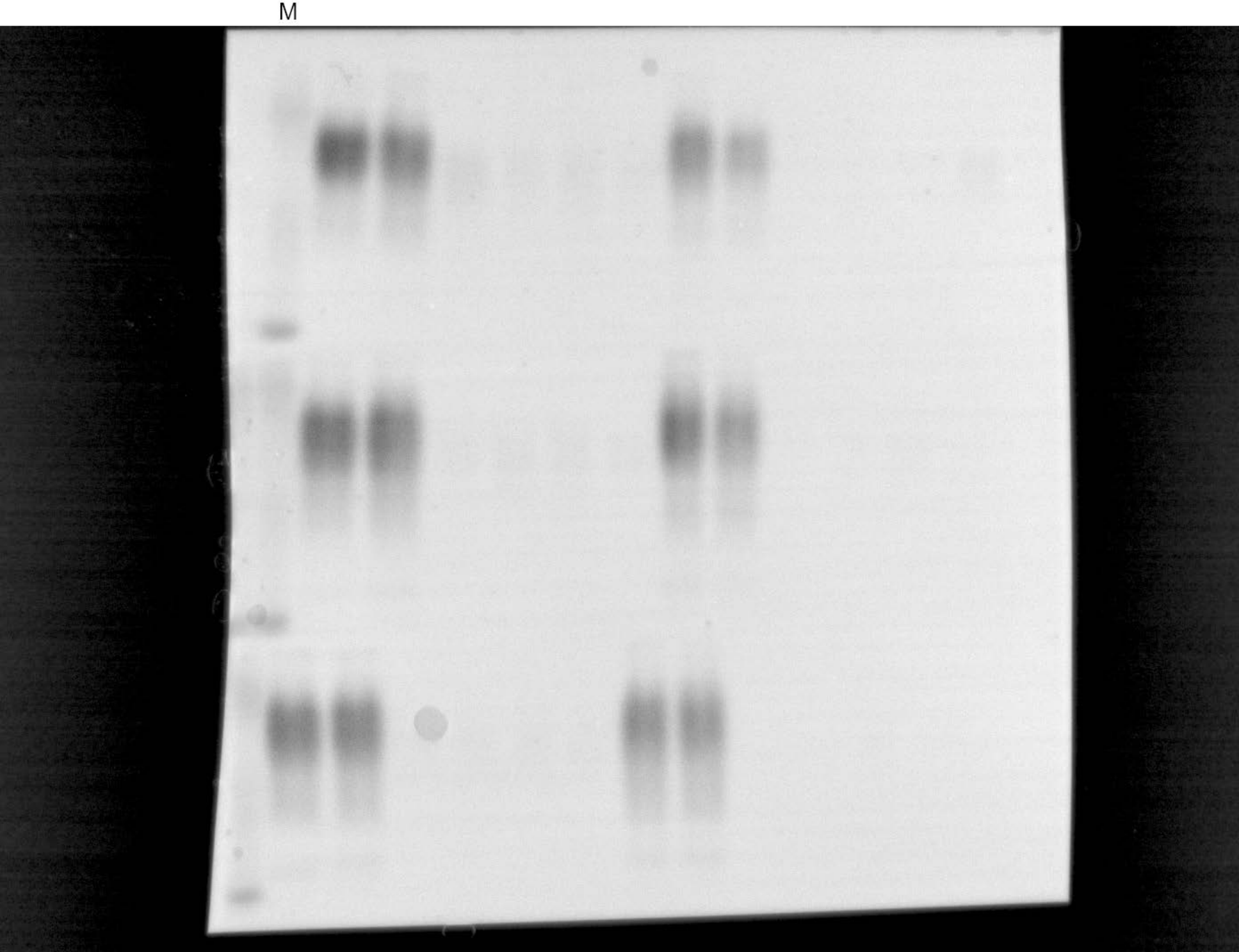

Fig. 3B, non-seeded DHA control. Original uncropped chemiluminescence image from an independent repeat experiment.
This image shows the non-seeded DHA PMCA control corresponding to Fig. 3B. The non-seeded control was repeated under the same substrate and cofactor conditions because the original uncropped source image for the initially assembled panel was not retained. The corresponding panel in Fig. 3B was replaced with this independent repeat experiment. The molecular weight marker lane (M) is indicated on the left.

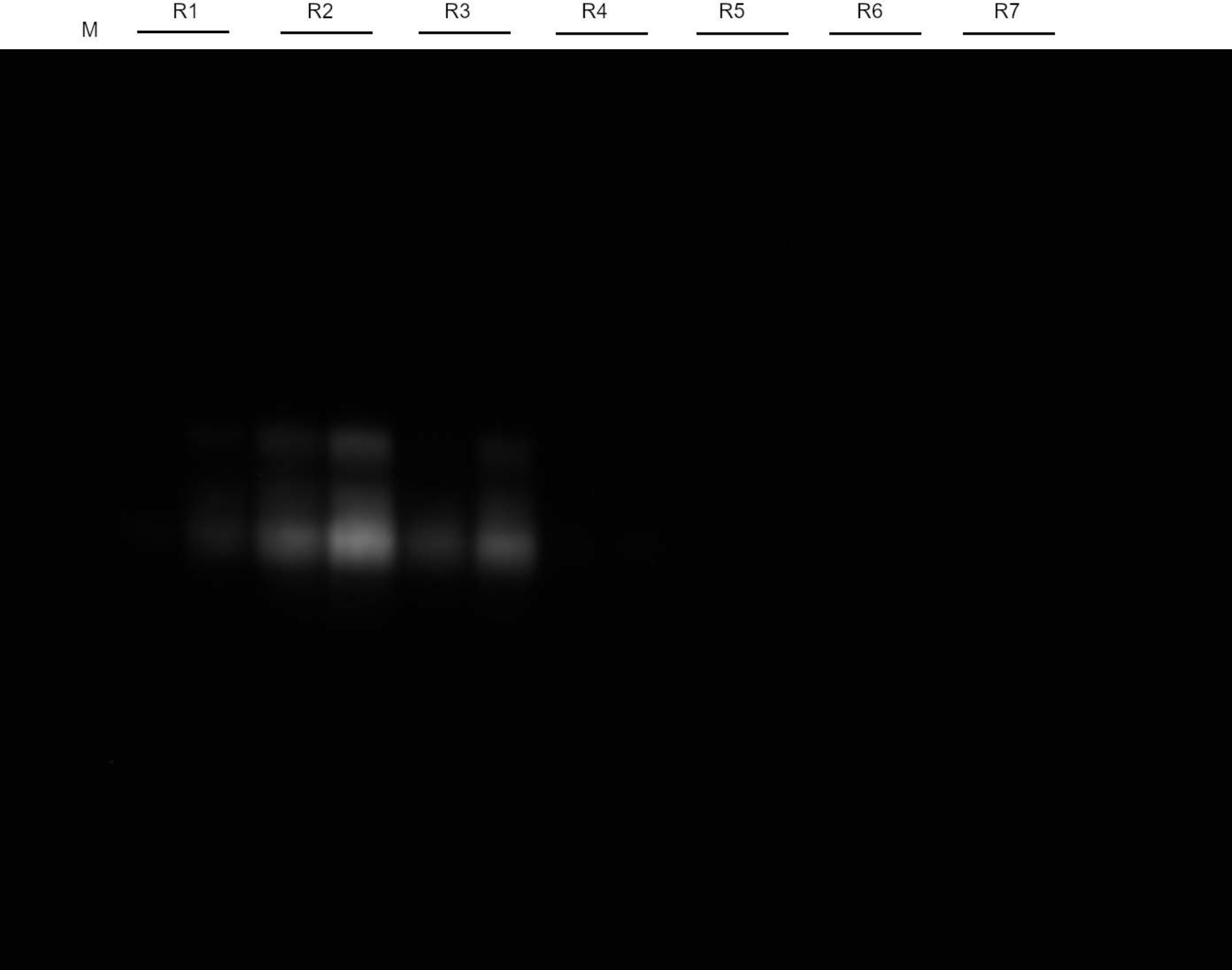

Fig. 3B, non-seeded DHA control. Contrast-enhanced image of the same membrane.
This contrast-enhanced image was generated only to visualize the molecular weight marker lane (M) and assign molecular-weight positions; it was not used for PrPres band interpretation. The strong band-like signals observed in the R1–R3 lanes are considered nonspecific signals and were not interpreted as PrPres amplification. The molecular weight marker lane (M) is indicated on the left.

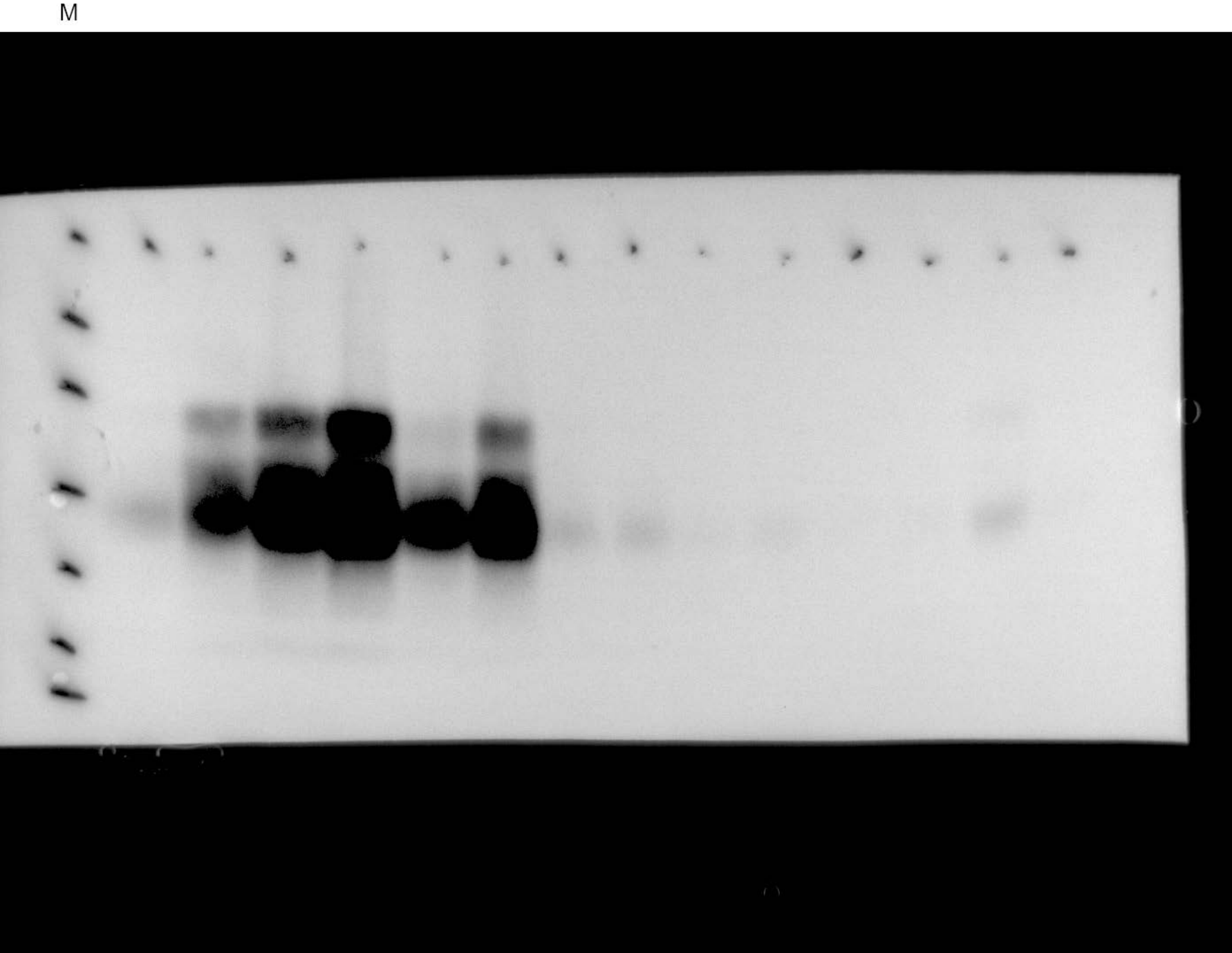

Fig. 3B, R1. Original uncropped chemiluminescence image.
This image shows the R1 result corresponding to Fig. 3B.
No molecular weight marker lane was included in this blot.

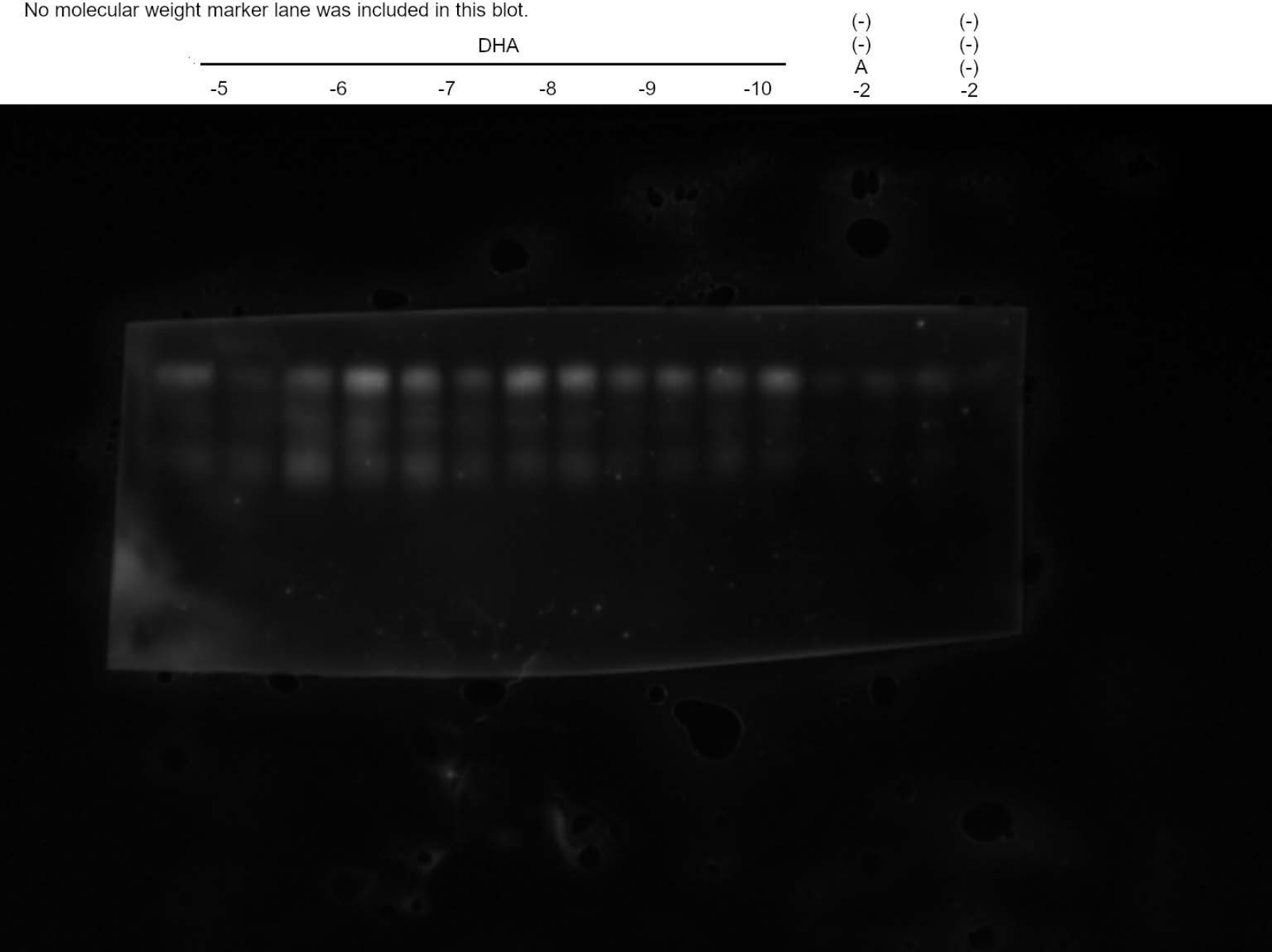

Fig. 3B, R2/R3. Original uncropped chemiluminescence image.
Three gels were transferred onto a single PVDF membrane and detected simultaneously.
This image shows the R2 and R3 results corresponding to Fig. 3B.
Of the three gel images transferred onto this membrane, only the middle and bottom gel images were used for Fig. 3B; the top gel image was not used in the final figure.
The molecular weight marker lane (M) is indicated; however, a separate molecular weight marker image was not captured for this blot.

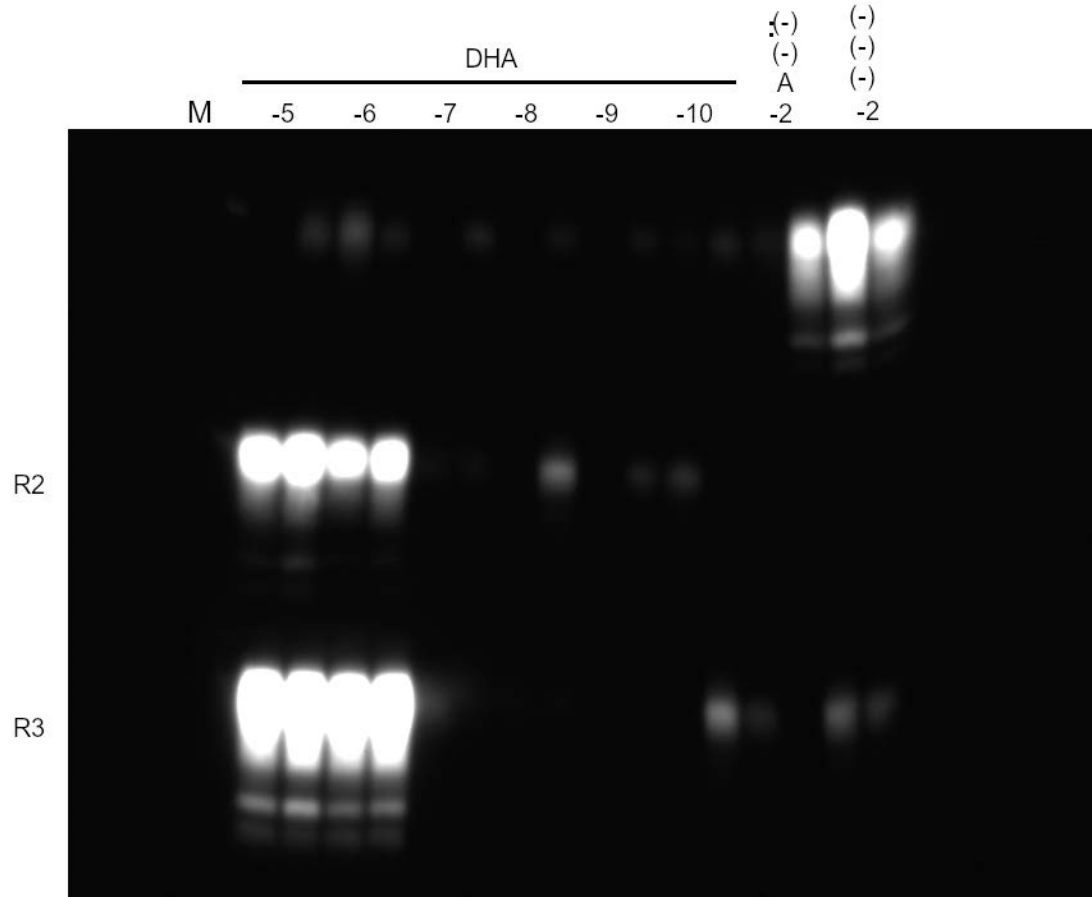

Fig. 3B, R4/R5. Original uncropped chemiluminescence image.
Two gels were transferred onto a single PVDF membrane and detected simultaneously.
This image shows the R4 and R5 results corresponding to Fig. 3B.
The molecular weight marker lane position (M) is indicated; however, n image of the molecular weight marker was not captured for this blot.

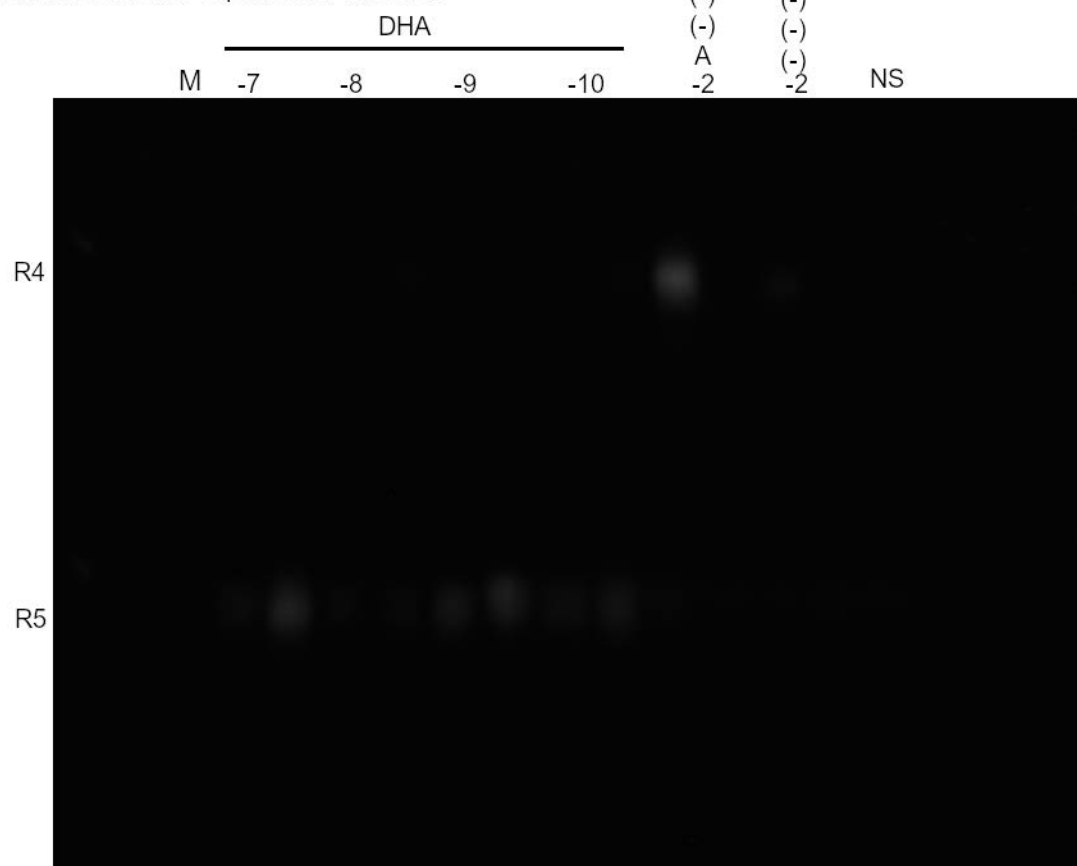

Fig. 3B, R6/R7 negative controls. Original uncropped chemiluminescence image.
Two gels were transferred onto a single PVDF membrane and detected simultaneously.
This image shows the R6 and R7 negative-control results corresponding to Fig. 3B.
The negative-control lanes used for Fig. 3B are indicated on the right.
The molecular weight marker lane (M) is indicated; however, a separate molecular weight marker image was not captured for this blot.

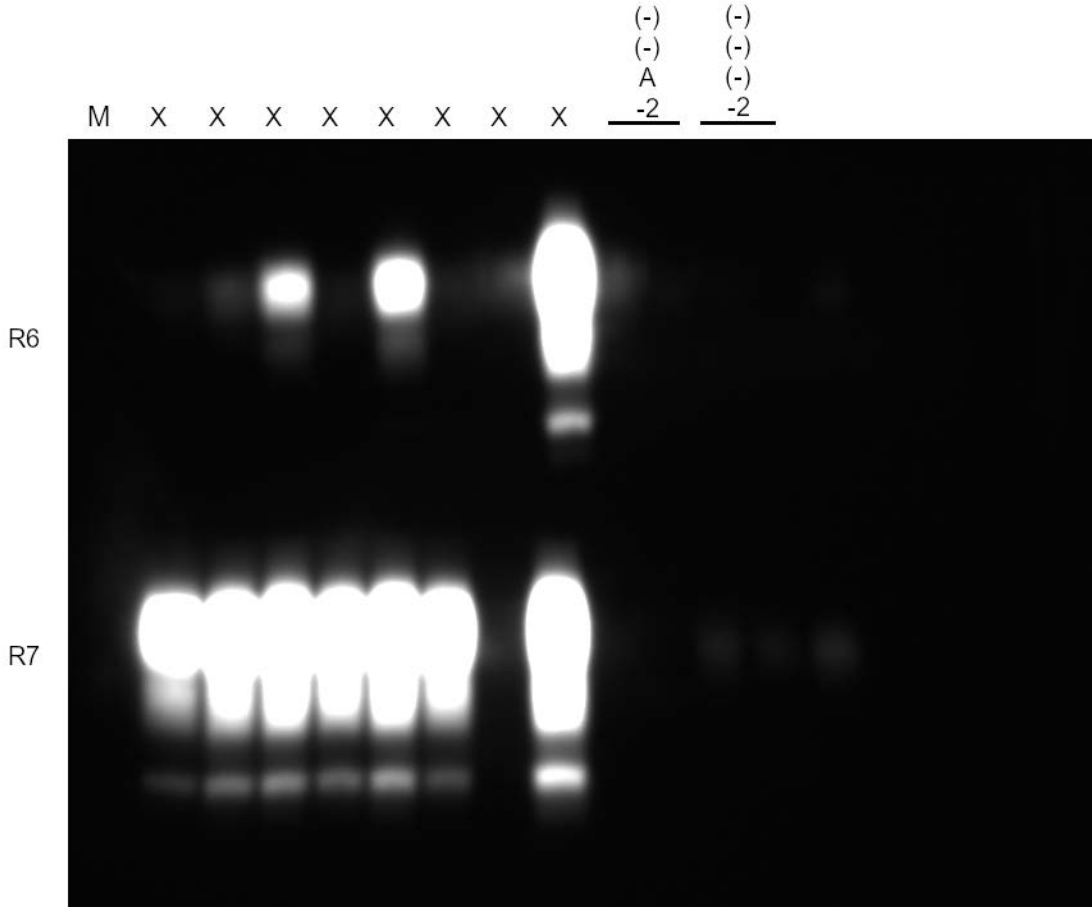

Fig. 3B, R6/R7. Original uncropped chemiluminescence image.
This image shows the R6 and R7 results corresponding to Fig. 3B.
The molecular weight marker lane position (M) is indicated; however, n image of the molecular weight marker was not captured for this blot.

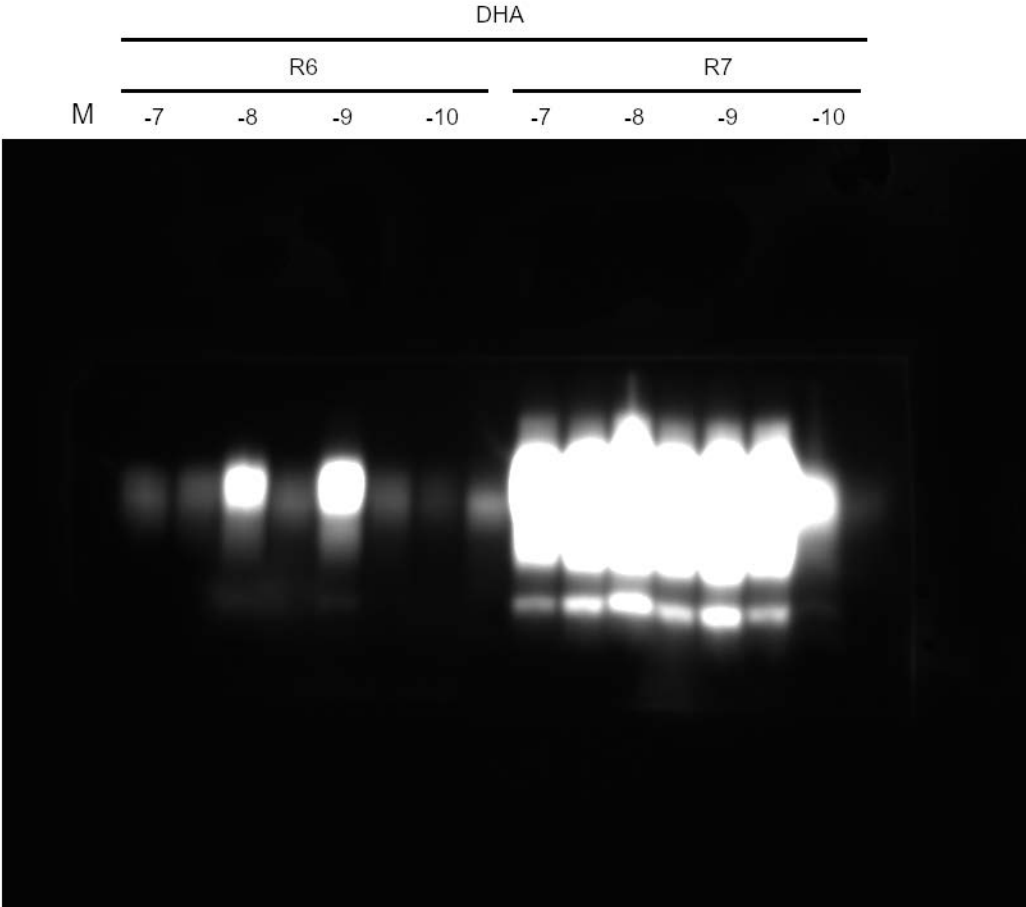

Fig. 4A, P4 panel. Original uncropped chemiluminescence image.
Lanes marked with X were not used in the final figure.
The lanes used for the P4-probed Western blot panel in Fig. 4A are indicated on the right.
The molecular weight marker lane (M) is indicated to the left of the P4 lanes.

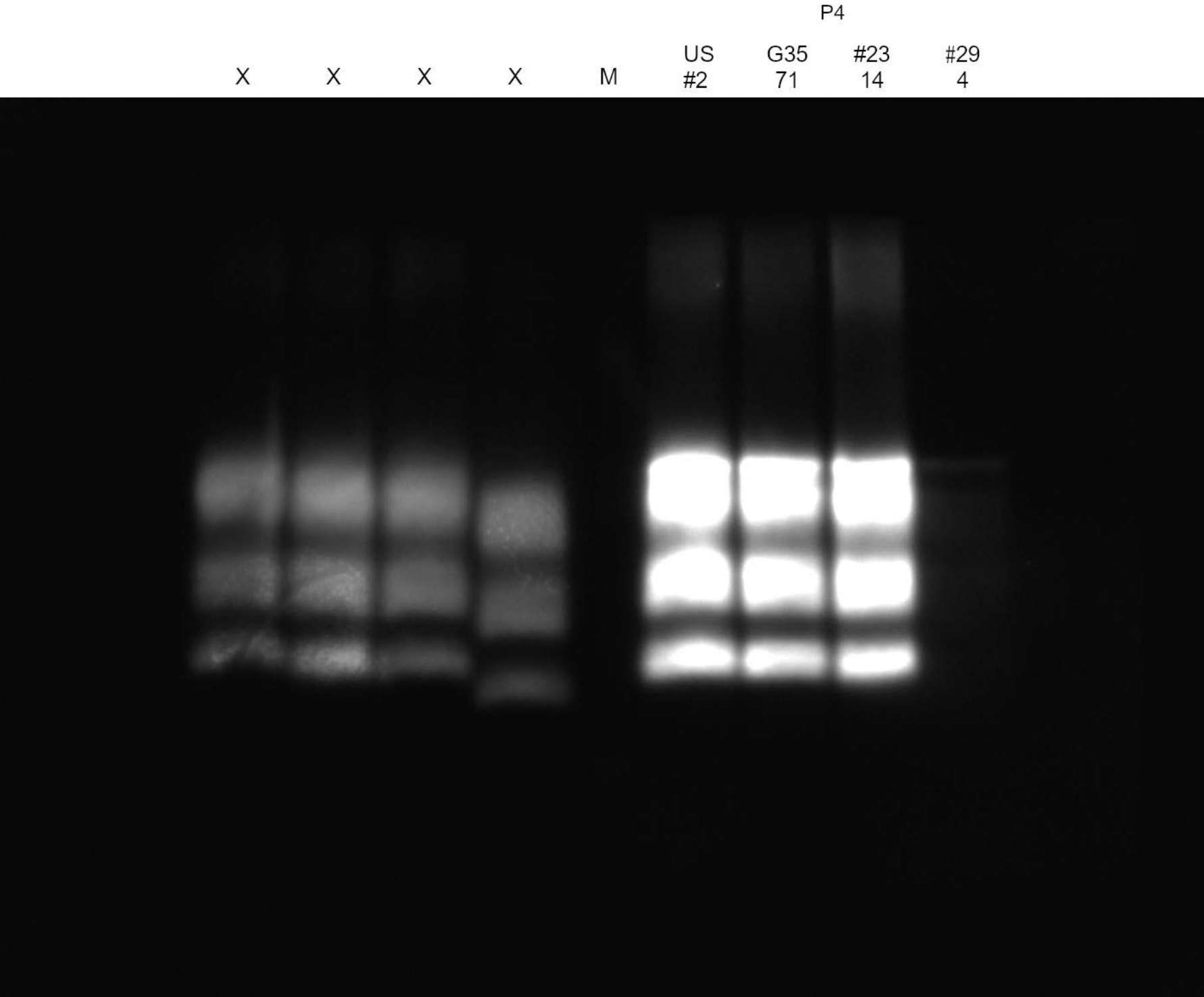

Fig. 4A, P4 panel. Contrast-enhanced image of the same membrane.
This image is shown only to visualize the molecular weight marker lane (M) and was not used for band interpretation.

M

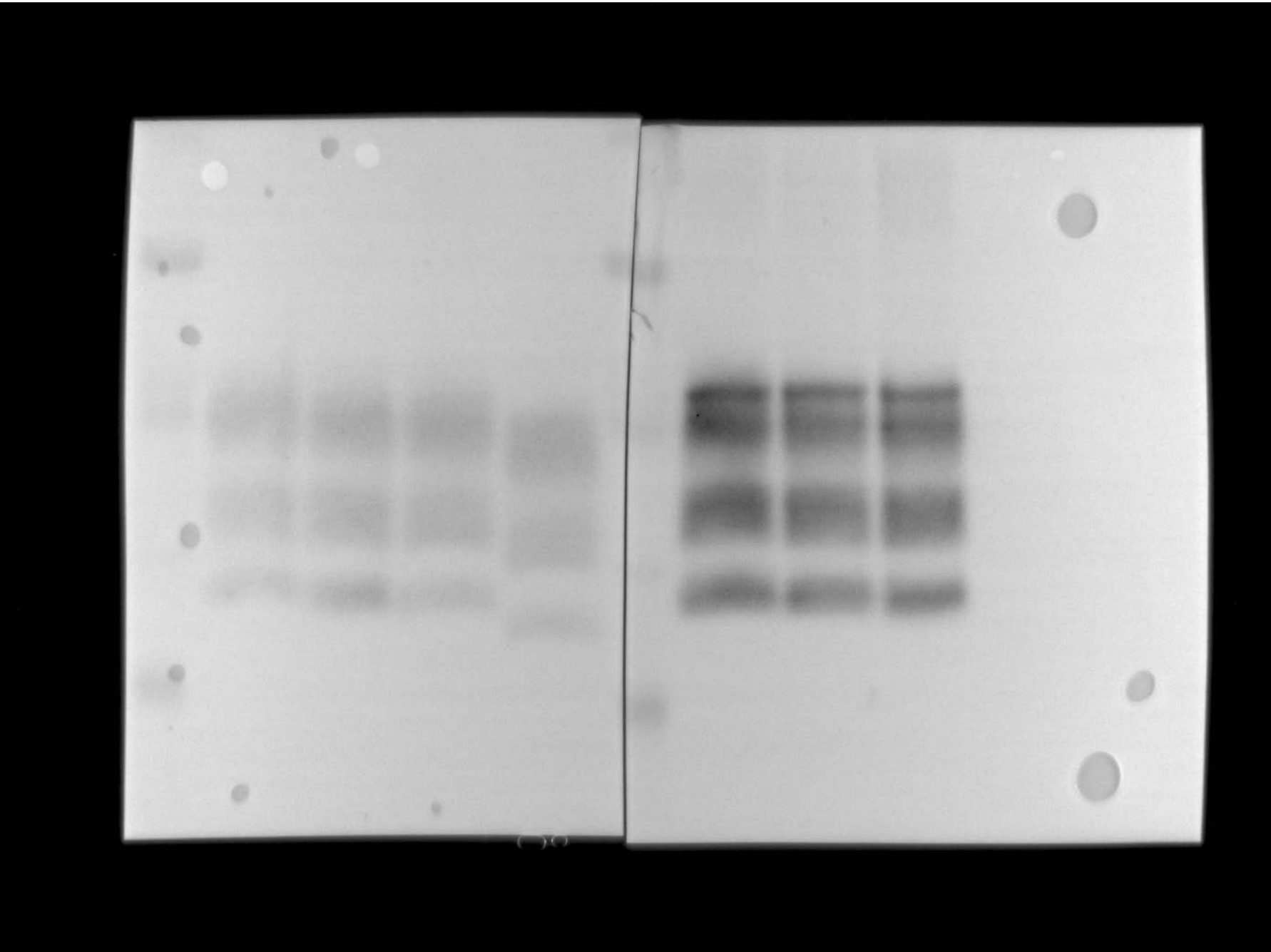

Fig. 4A, T2 panel. Original uncropped chemiluminescence image.
The molecular weight marker lane (M) is indicated to the left of the T2 lanes.

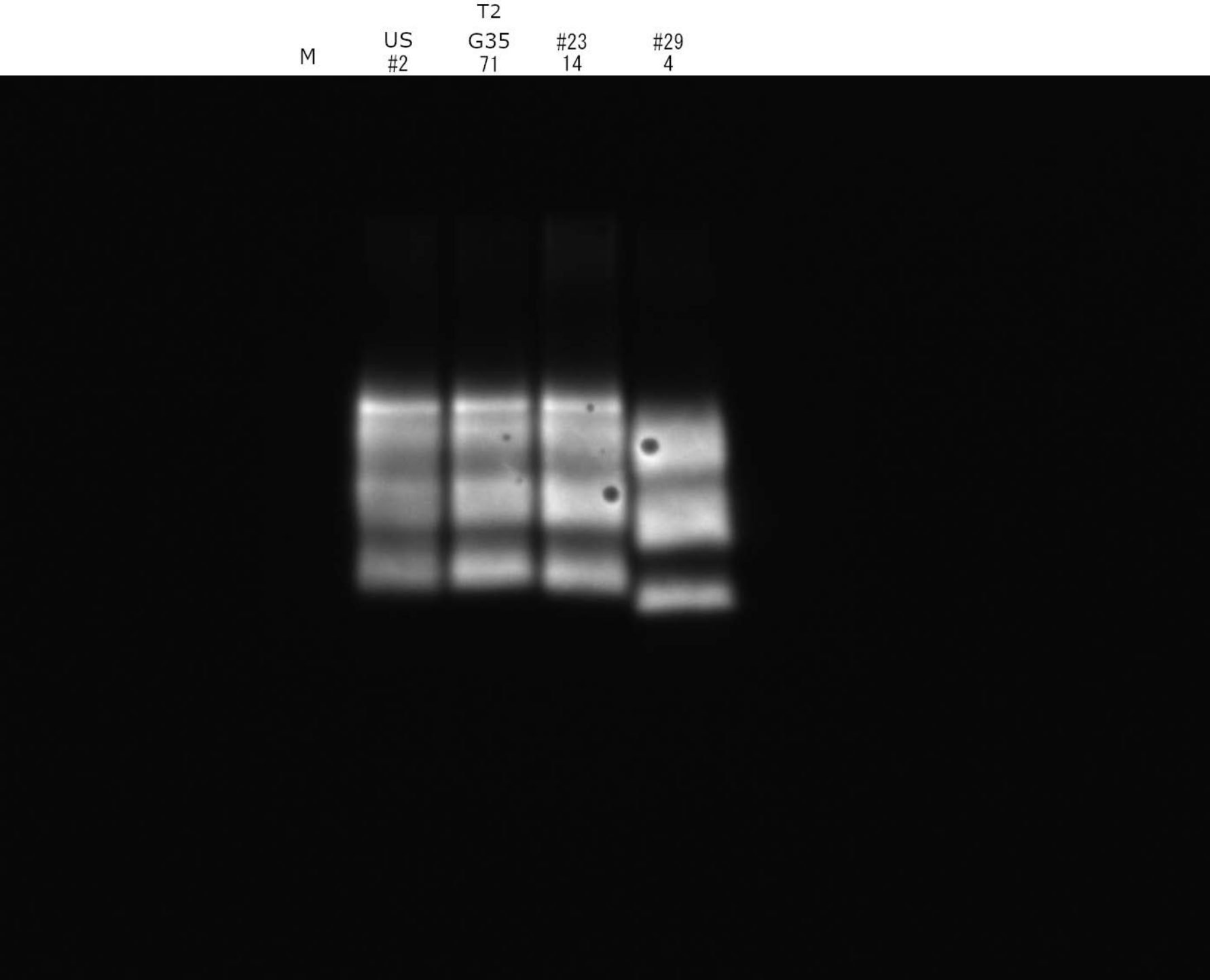

Fig. 4A, T2 panel. Contrast-enhanced image of the same membrane.
This image is shown only to visualize the molecular weight marker lane (M) and was not used for band interpretation.

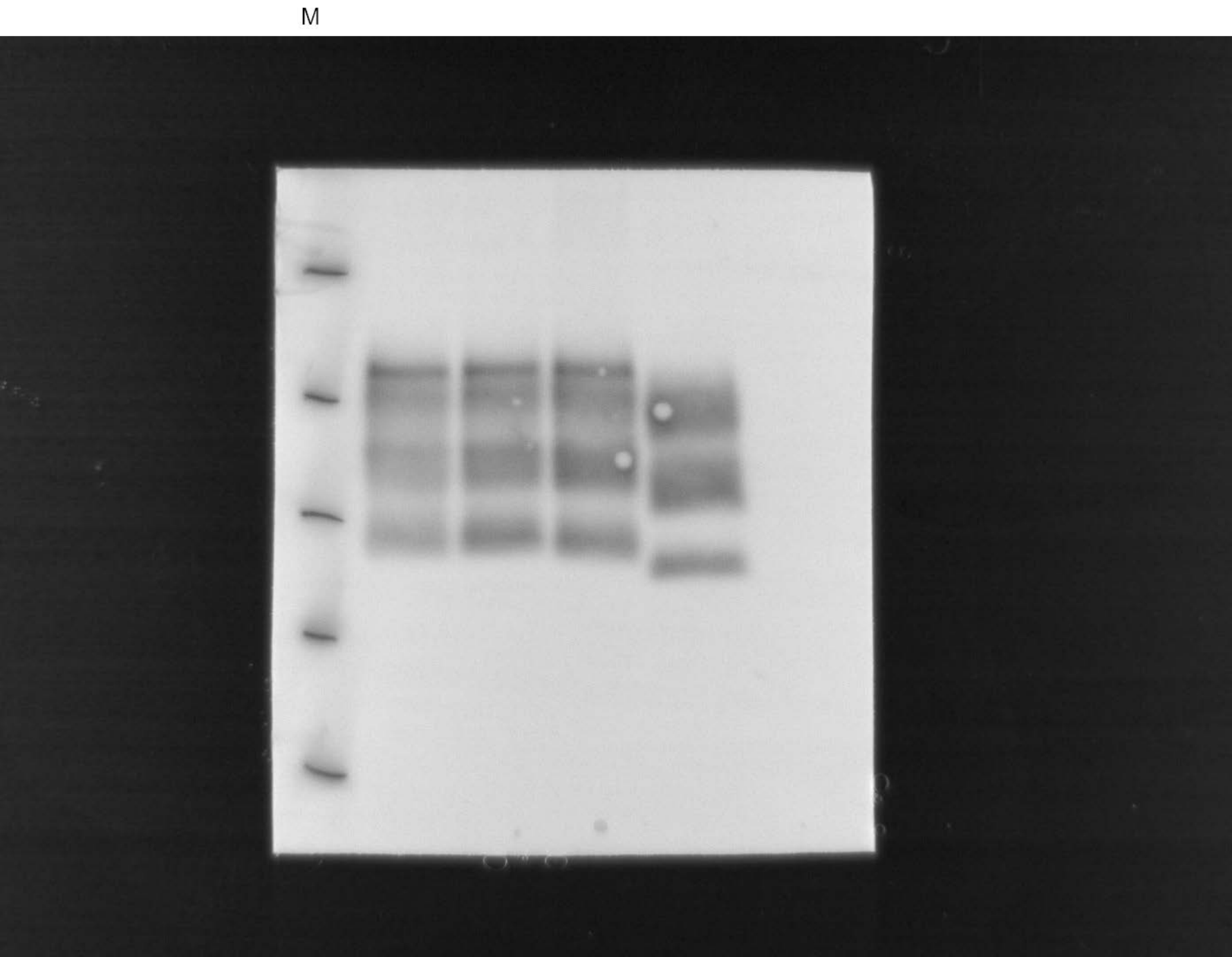

Fig. 4B Original uncropped chemiluminescence image.
Two gels were transferred onto a single PVDF membrane and detected simultaneously.
The upper and lower blot images correspond to -AE and +AE conditions, respectively.
The molecular weight marker lane (M) is indicated, although the marker bands are not visible in this chemiluminescence exposure because a chemiluminescent marker was not used.

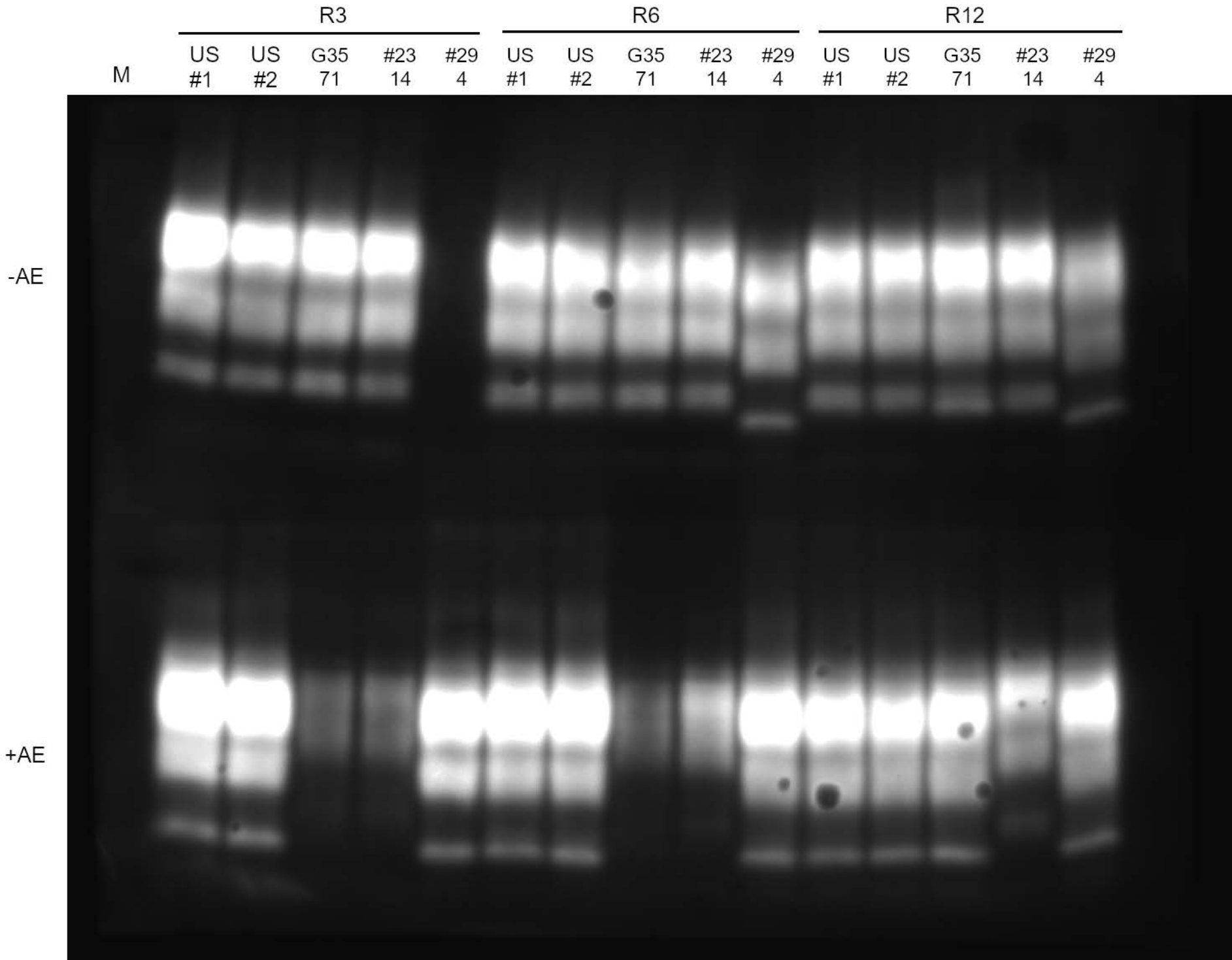

Fig. 4B. Contrast-enhanced image of the same membrane.
This image is shown only to visualize the molecular weight marker lane (M) and was not used for band interpretation.

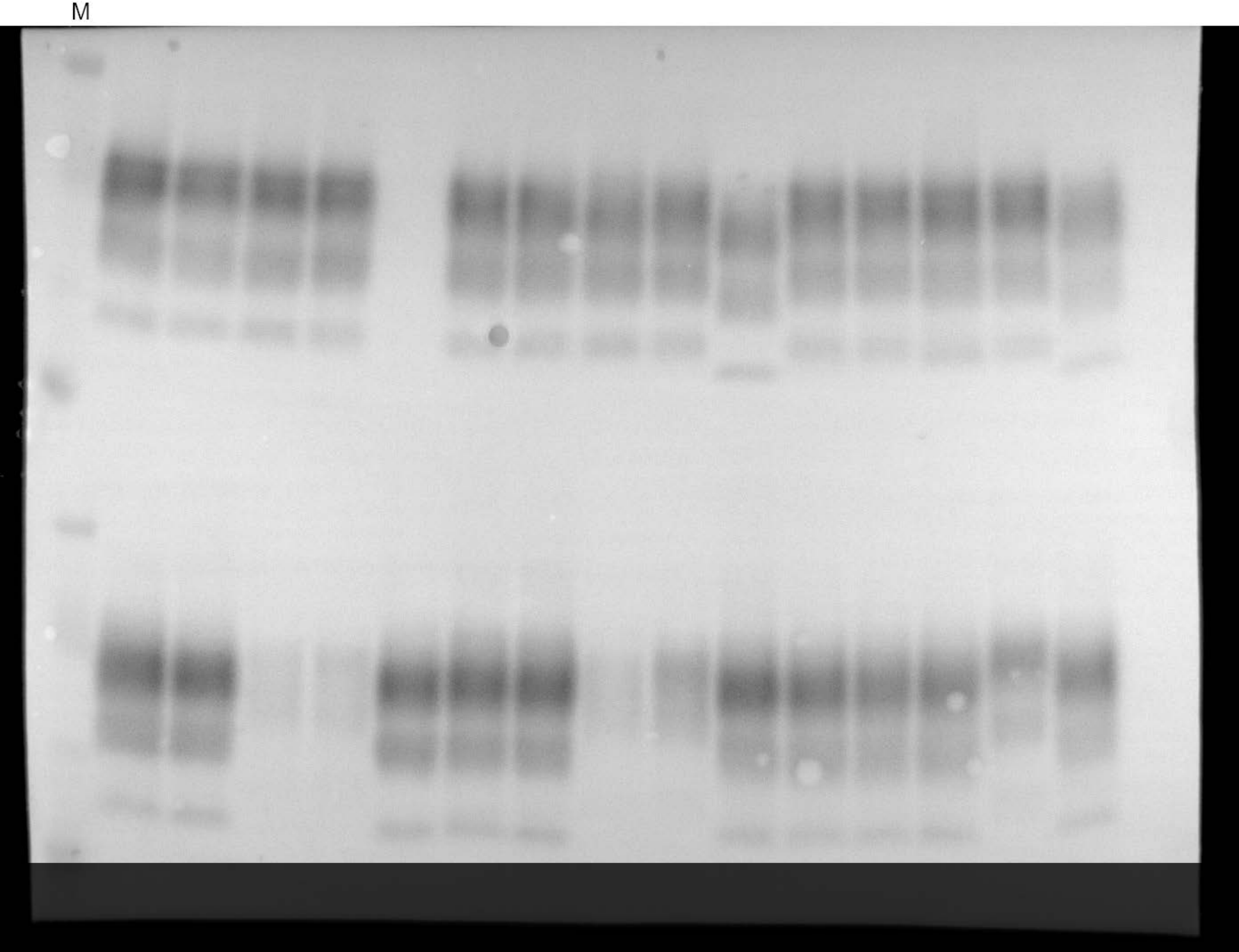

Fig. 4D. Original uncropped chemiluminescence image.
The molecular weight marker lane (M) is indicated, although the marker bands are not visible in this chemiluminescence exposure because a chemiluminescent molecular weight marker was not used.

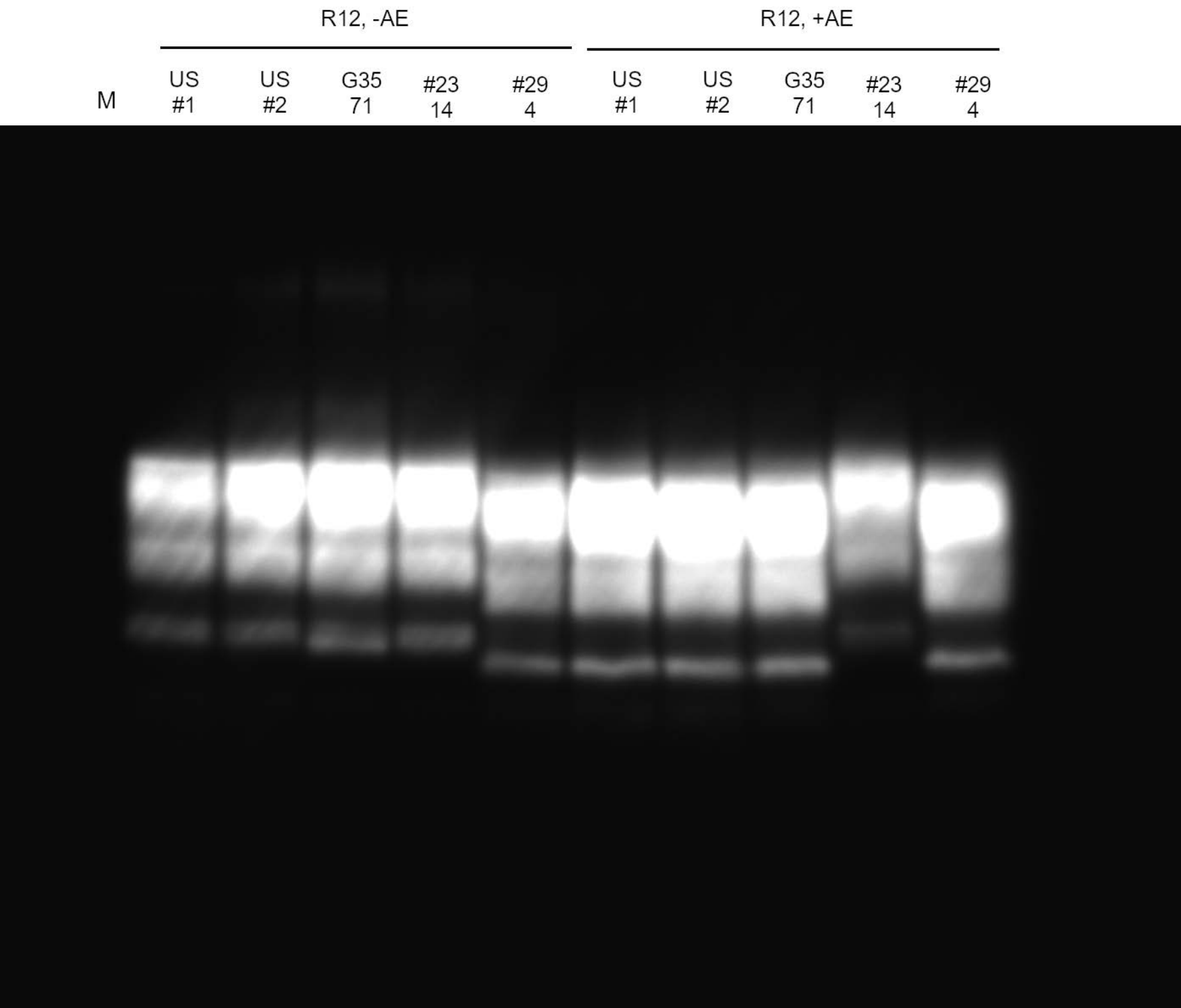

Fig. 4D. Contrast-enhanced image of the same membrane.
This image is shown only to visualize the molecular weight marker lane (M) and was not used for PrPres band interpretation.

M

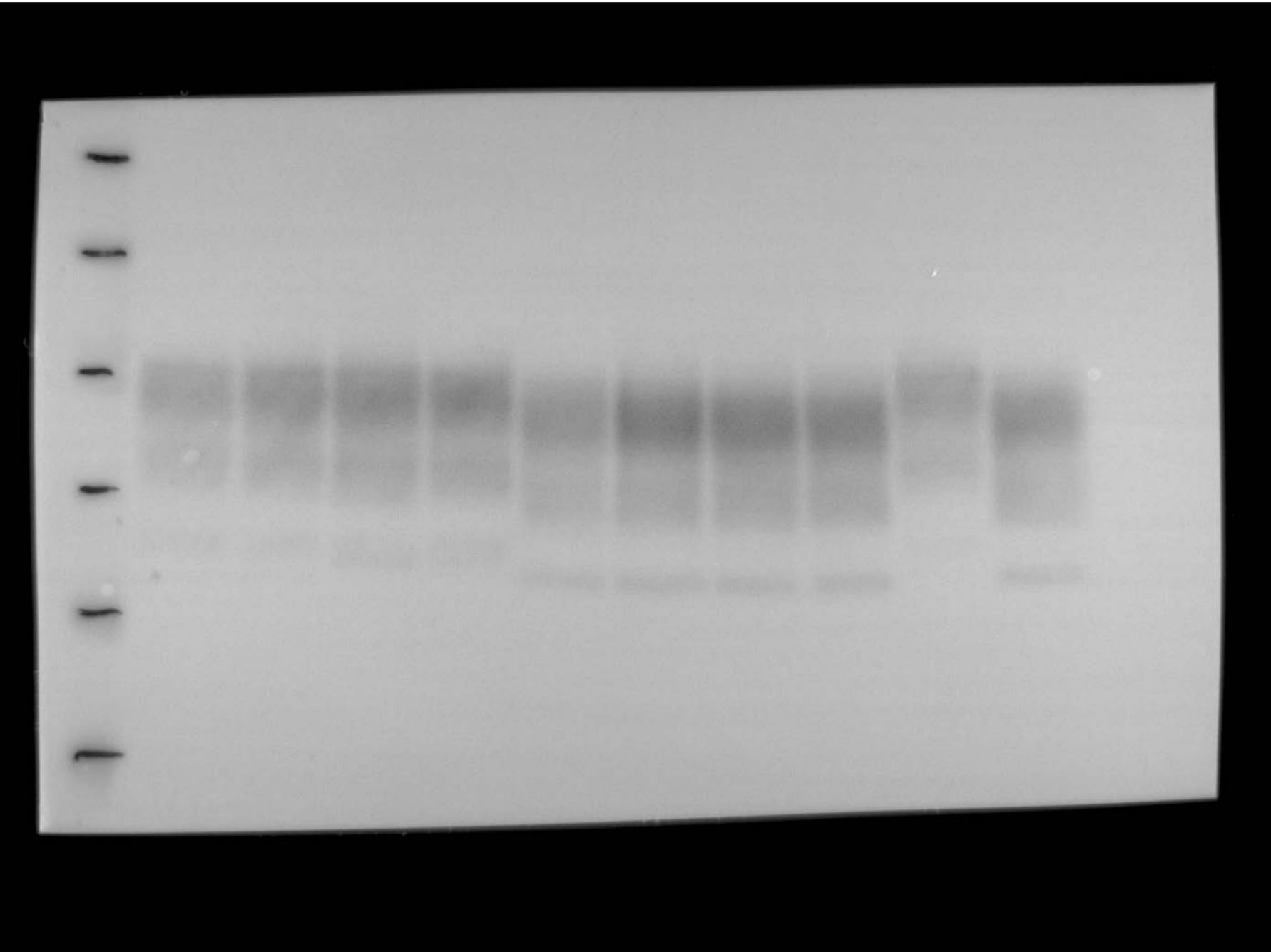

Fig. 5A. Original uncropped chemiluminescence image.
Two gels were transferred onto a single PVDF membrane and detected simultaneously.
The upper gel image shows the results corresponding to Fig. 5A.
The lower gel image was not used in the final figure.
The molecular weight marker lanes (M) are indicated on both sides of the upper gel image.

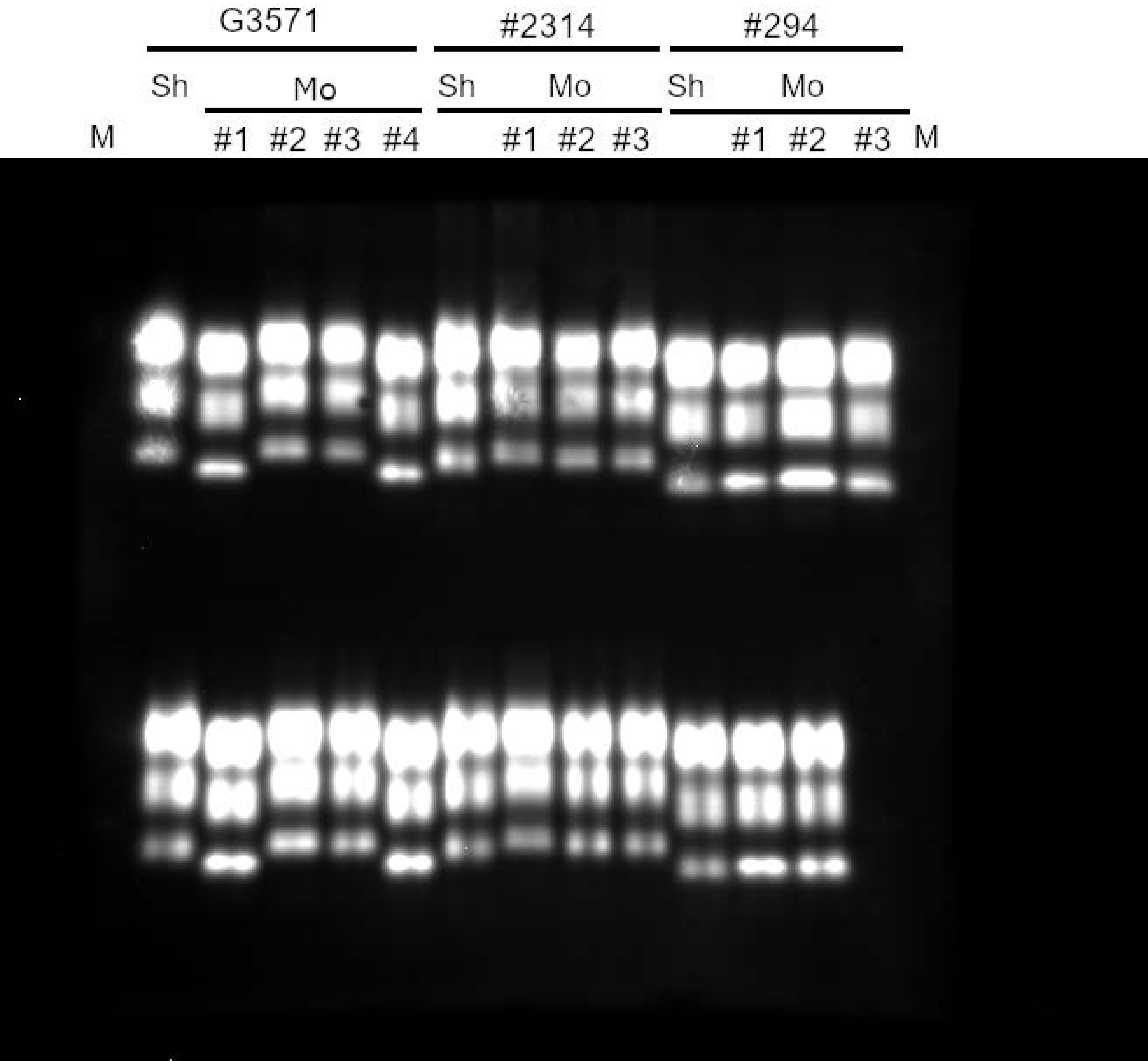

Fig. 5A. Contrast-enhanced image of the same membrane.
This image is shown only to visualize the molecular weight marker lanes (M) and was not used for PrPres band interpretation.

M M

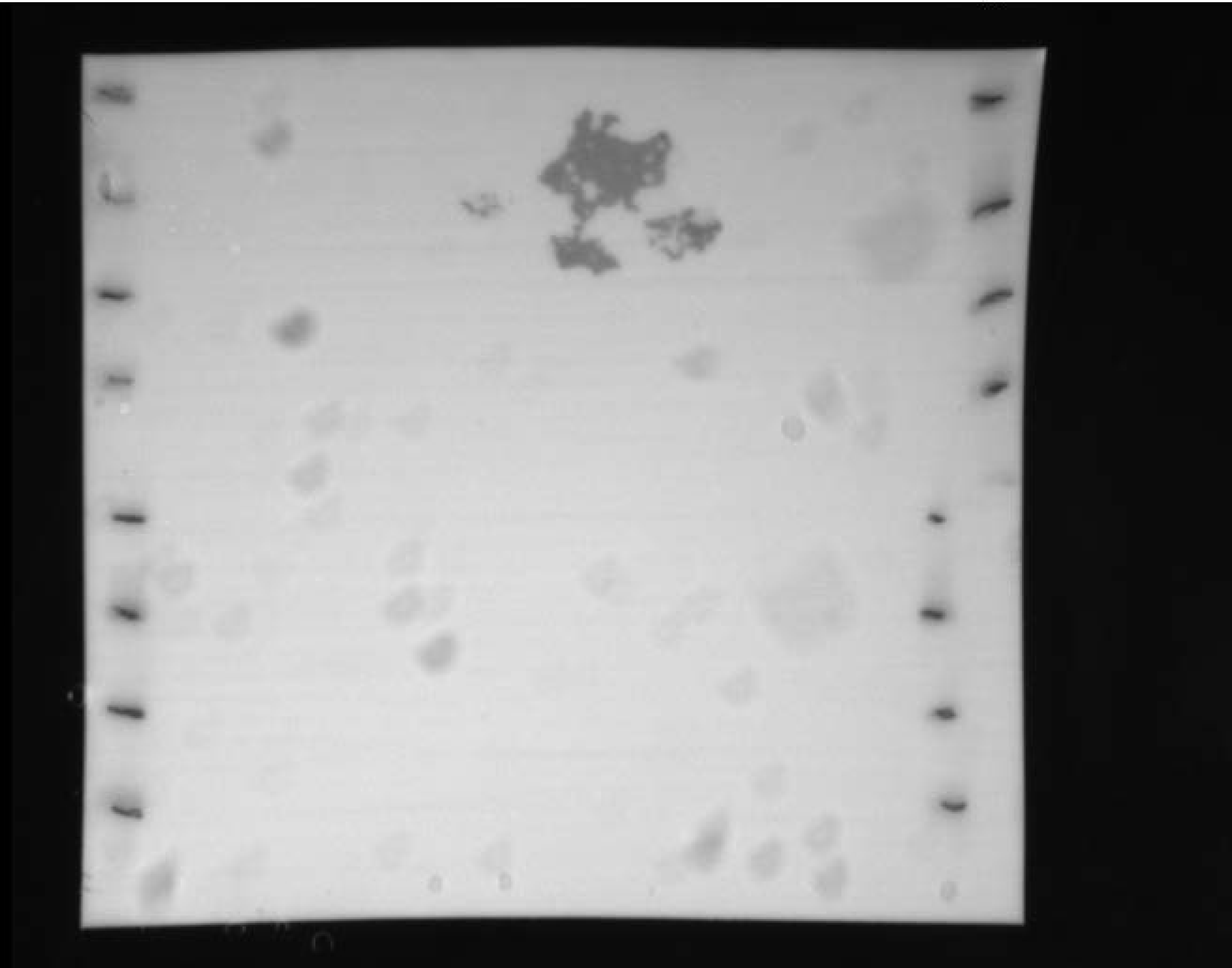

Fig. 5B and 5C. Original uncropped chemiluminescence image.
Two gels were transferred onto a single PVDF membrane and detected simultaneously.
The upper and lower gel images correspond to Fig. 5B and Fig. 5C, respectively.
The molecular weight marker lane (M) is indicated on the left.

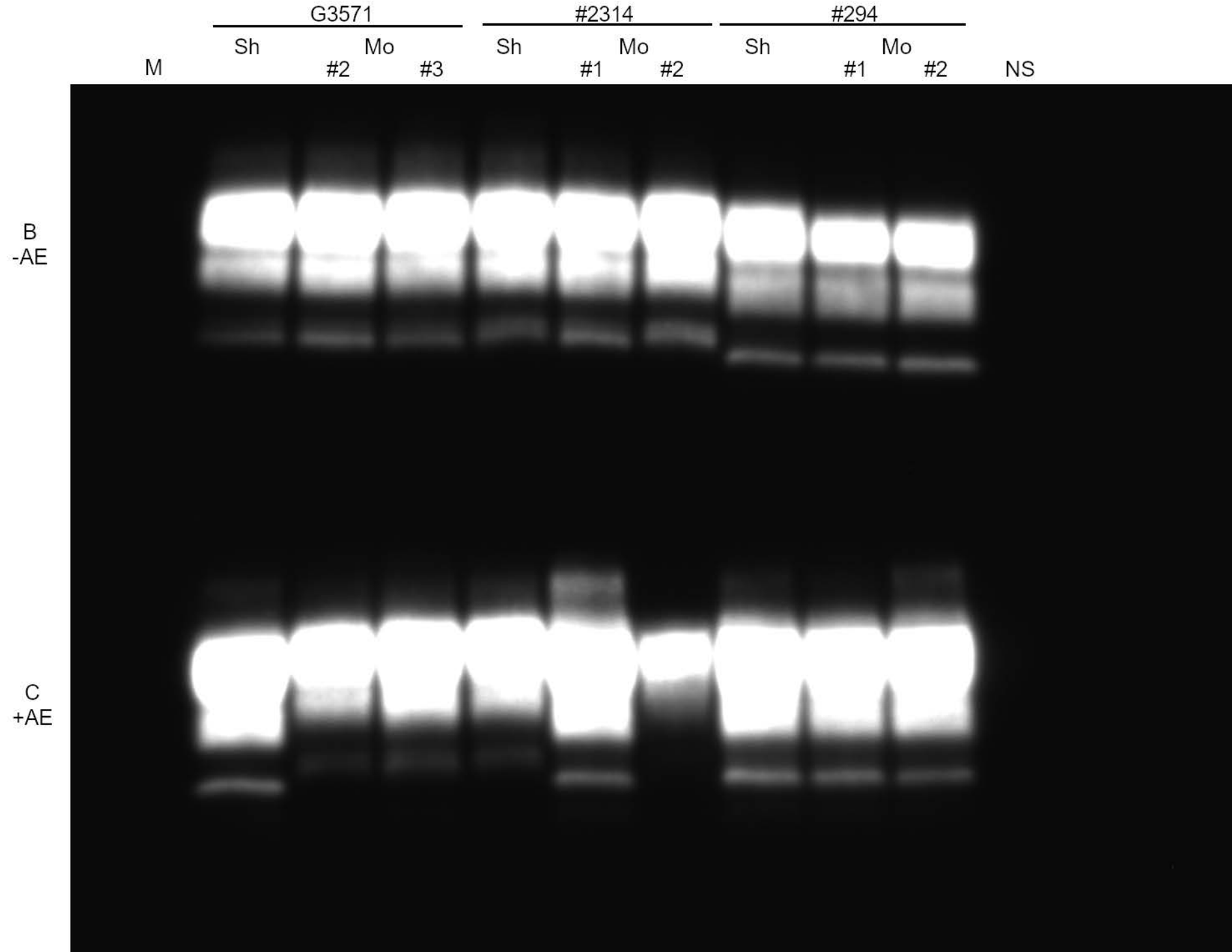

Fig. 5B and 5C. Contrast-enhanced image of the same membrane.
This image is shown only to visualize the molecular weight marker lane (M) and was not used for PrPres band interpretation.

M

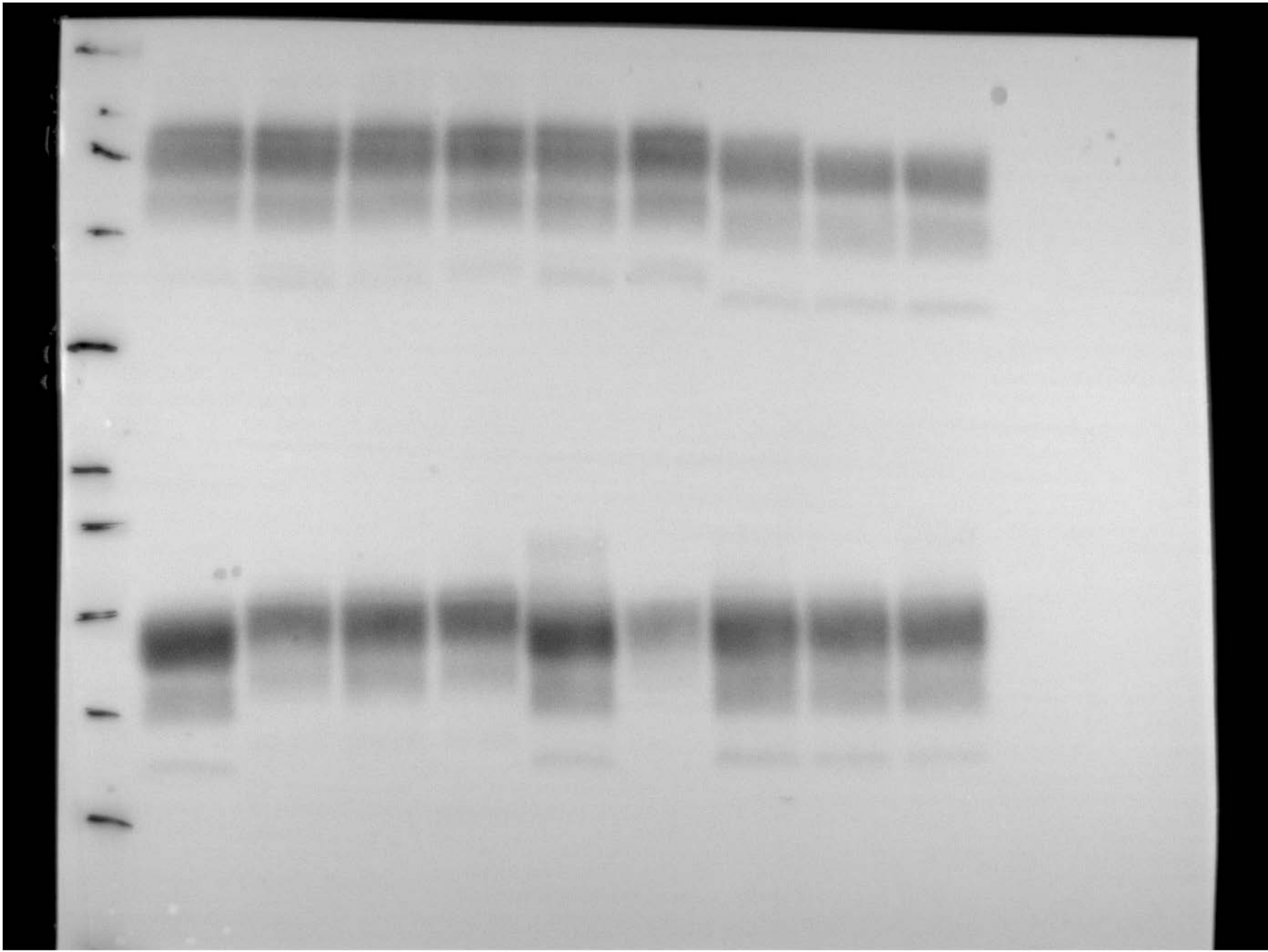

Fig. 5D, lower panel. Original uncropped chemiluminescence image.
The molecular weight marker lane (M) is indicated on the left.

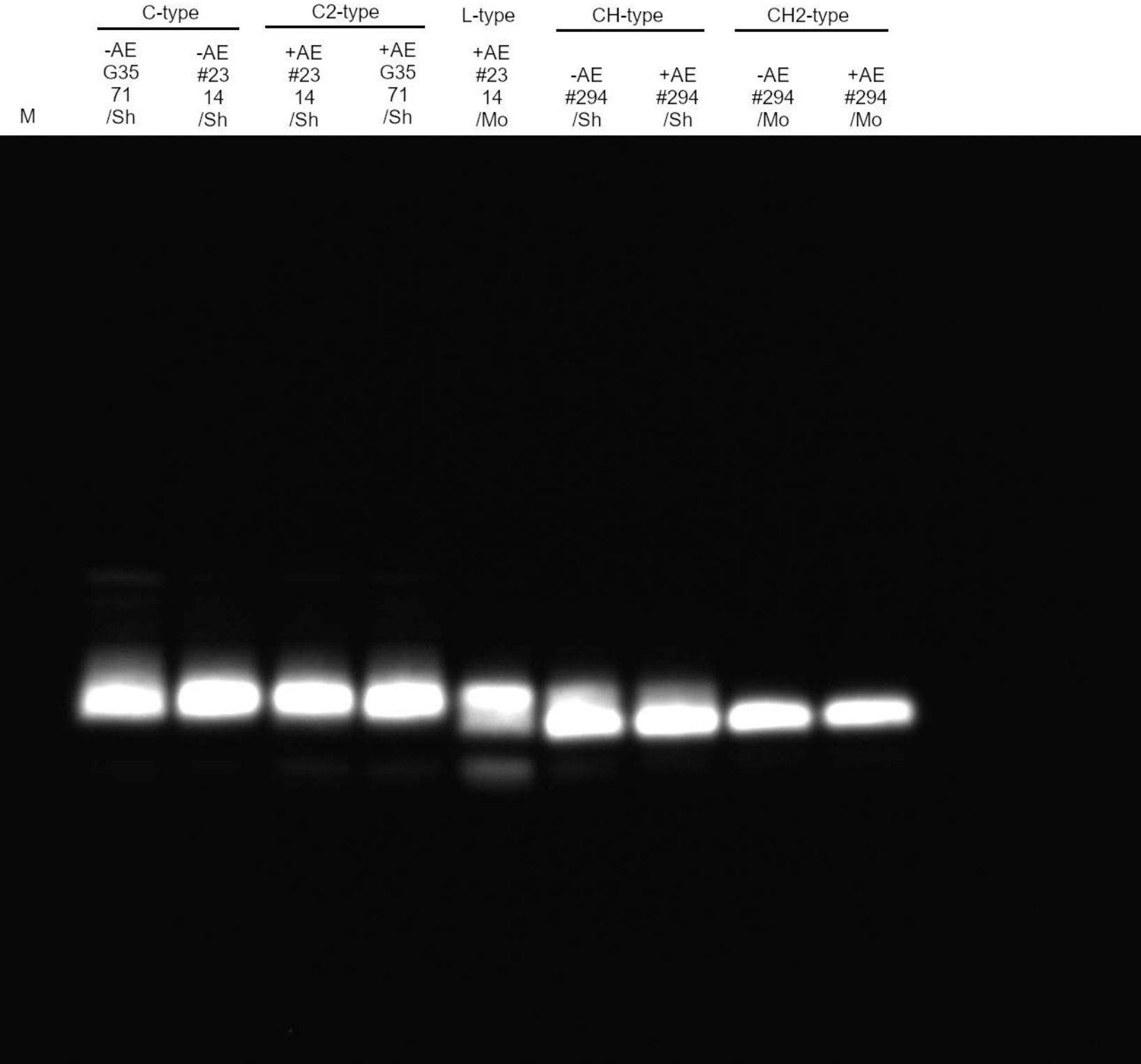

Fig. 5D, lower panel. Contrast-enhanced image of the same membrane.
This image is shown only to visualize the molecular weight marker lane (M) and was not used for PrPres band interpretation.

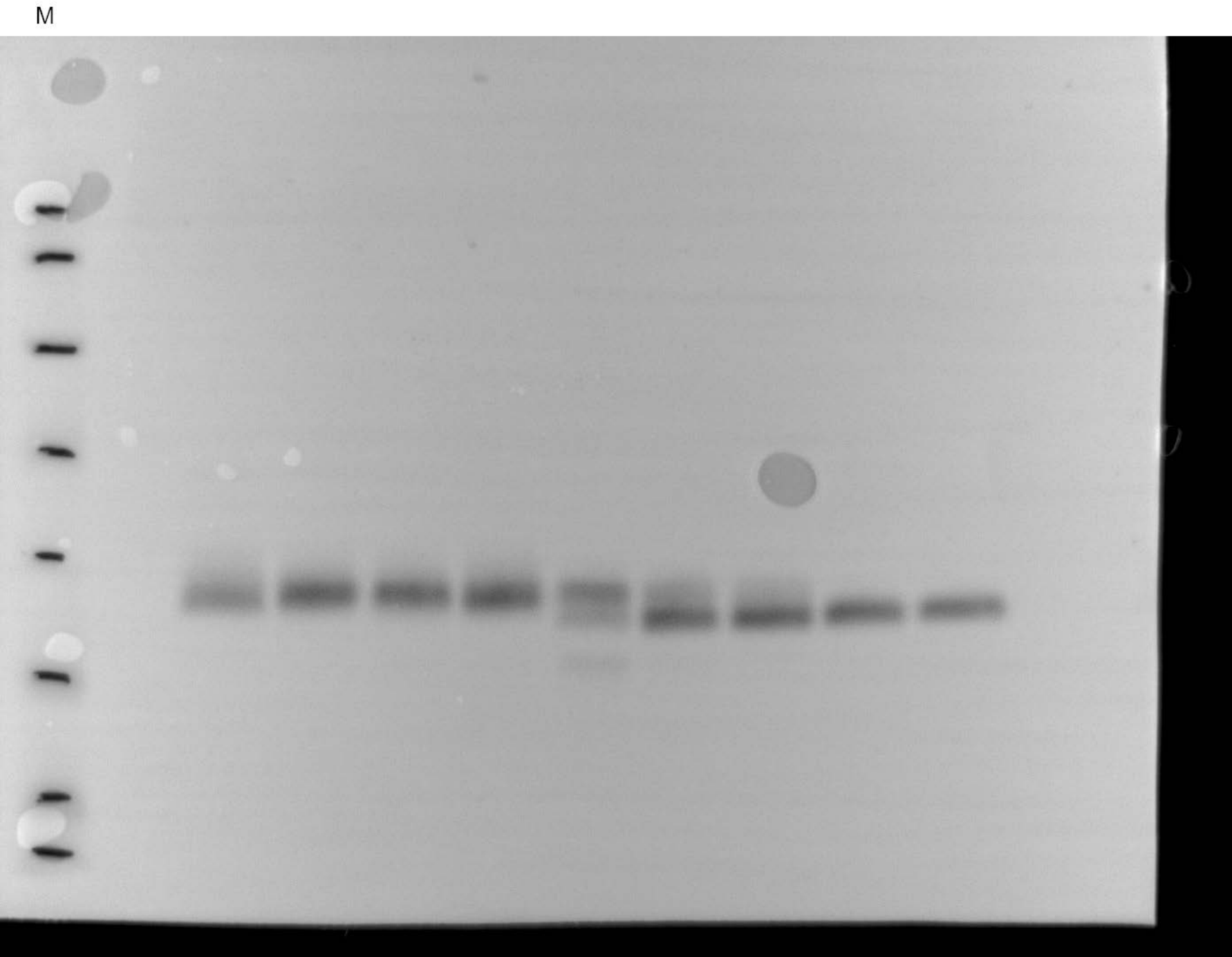

Fig. 5D, upper panel. Original uncropped chemiluminescence image.
The molecular weight marker lane (M) is indicated on the left.

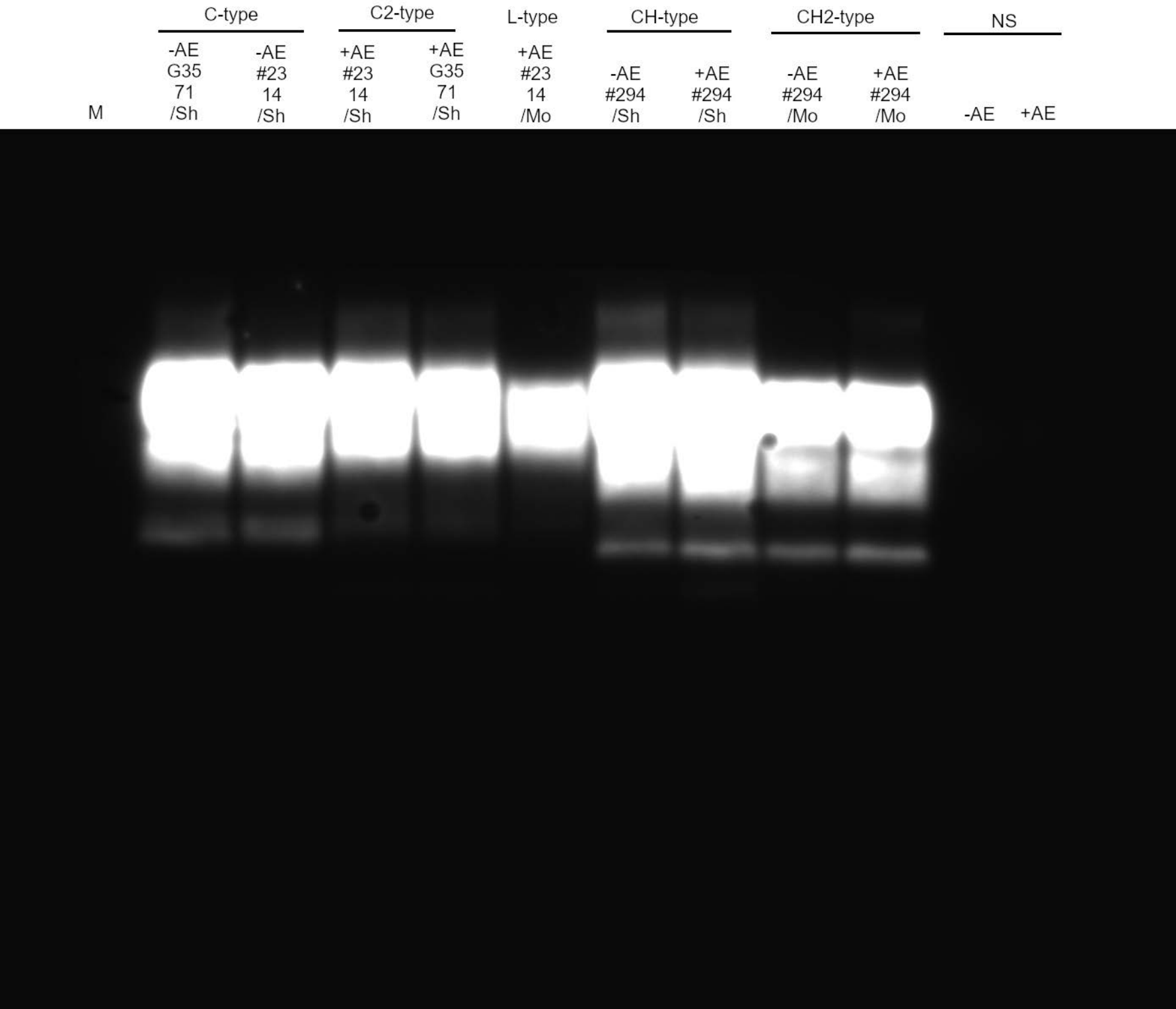

Fig. 5D, upper panel. Contrast-enhanced image of the same membrane.
This image is shown only to visualize the molecular weight marker lane (M) and was not used for PrPres band interpretation.

M

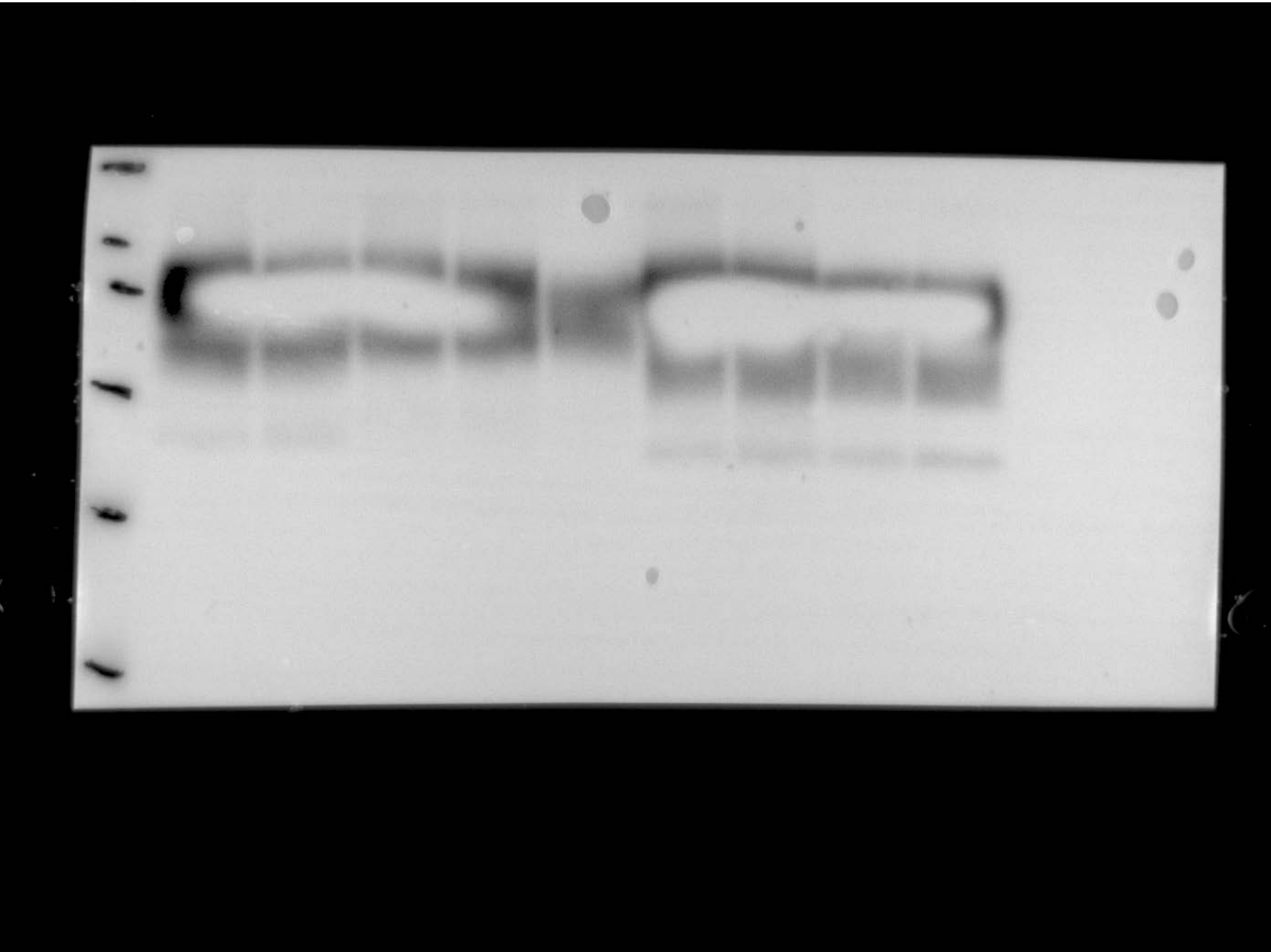

S1 Fig. Original uncropped chemiluminescence image.
Two gels were transferred onto a single PVDF membrane and detected simultaneously.
Only the lower gel image was used for the result shown in S1 Fig.; the upper gel image was not used in the final figure.
The molecular weight marker lane (M) is indicated on the left.

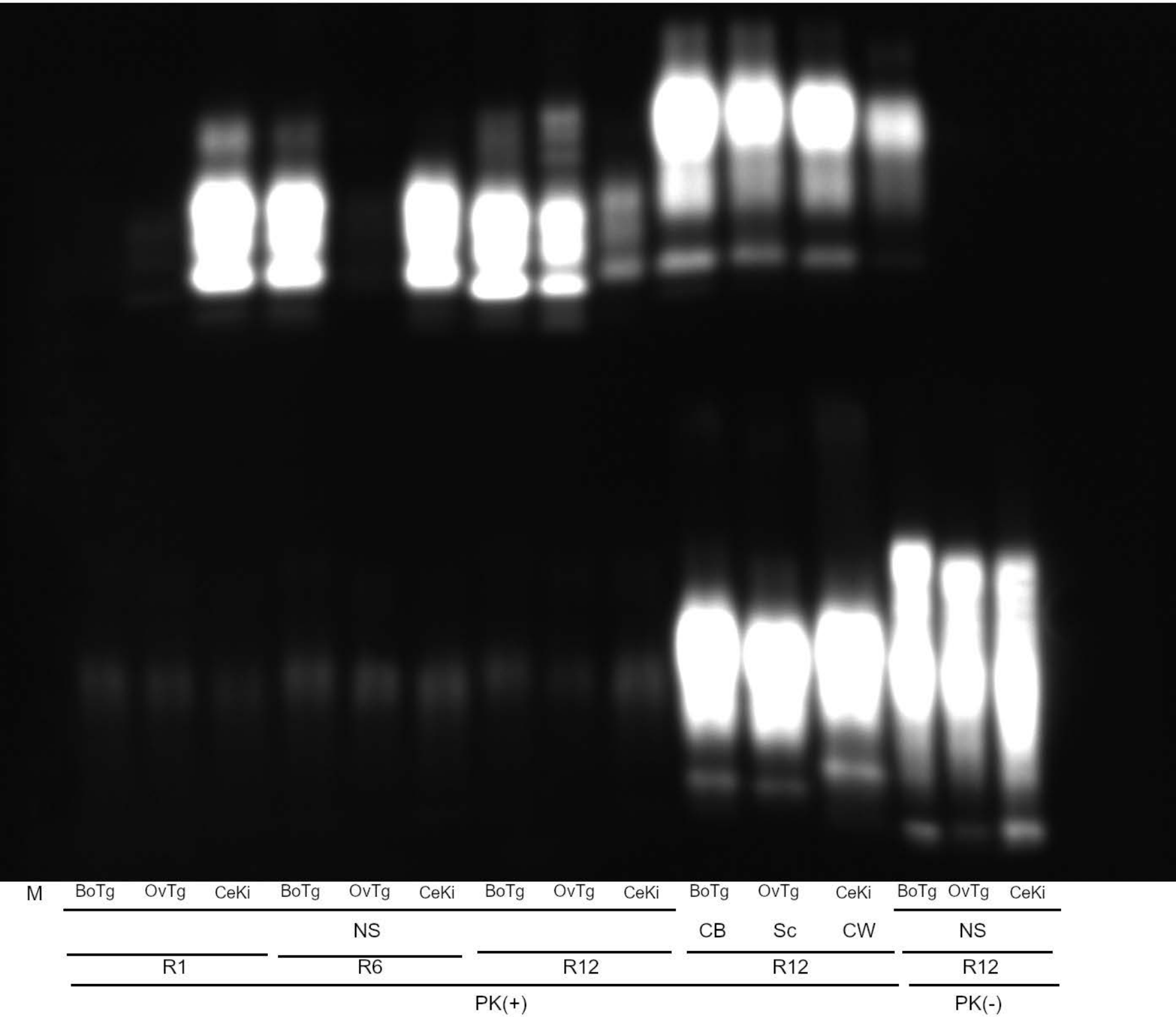

S1 Fig. Contrast-enhanced image of the same membrane.
Only the lower gel image was used for the result shown in S1 Fig. The upper gel image was not used in the final figure. This image is shown only to visualize the molecular weight marker lane (M) and was not used for PrPres band interpretation.

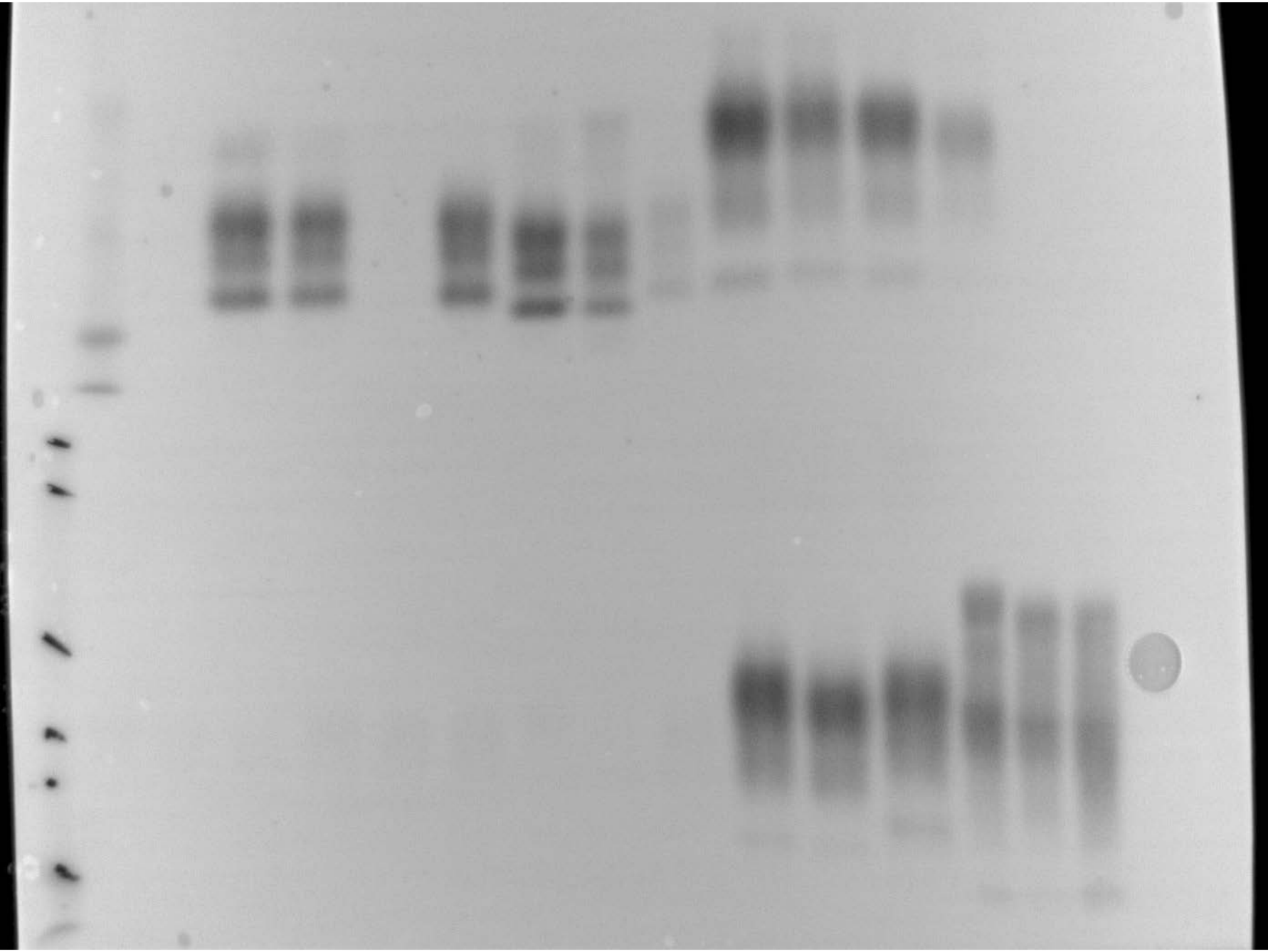

M

S2 Fig. Original uncropped chemiluminescence image.
Two gels were transferred onto a single PVDF membrane and detected simultaneously.
Only the lower gel image was used for the result shown in S2 Fig. The upper gel image was not used in the final figure.
The molecular weight marker lane (M) is indicated on the left.

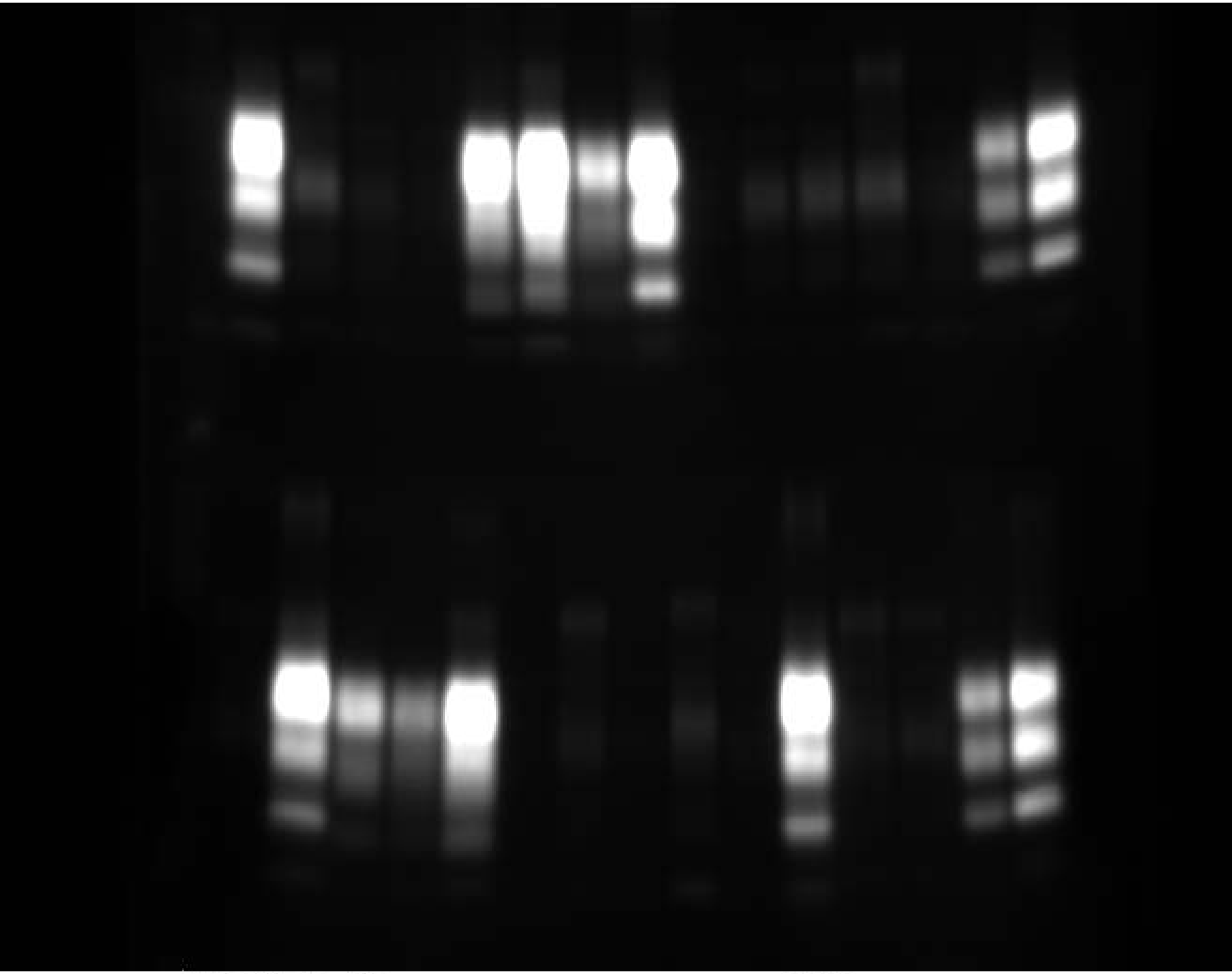

S2 Fig. White-light image of the same membrane.
This image was used only to visualize the molecular weight marker lane and to assign molecular-weight positions; it was not used to evaluate PrPres chemiluminescent signals.

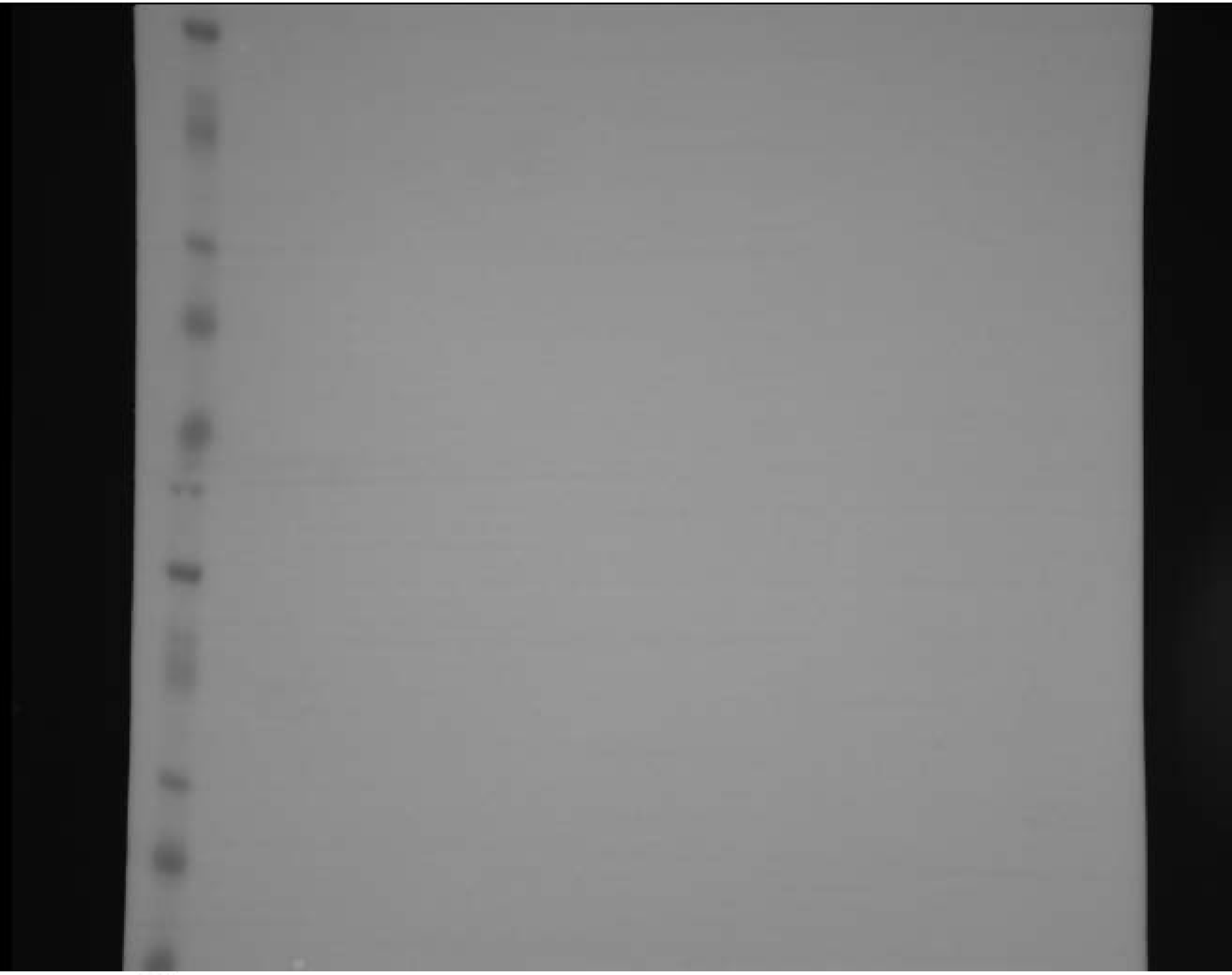

S4 Fig, panel A. Original uncropped chemiluminescence image.
Two gels were transferred onto a single PVDF membrane and detected simultaneously. This image shows the R12 products of twelve independent non-seeded PMCA reactions performed using OvTg BH under DH(-A) and DHA conditions. Numbers above the lanes indicate independent reaction numbers. Lanes marked with X were not used in the final figure.

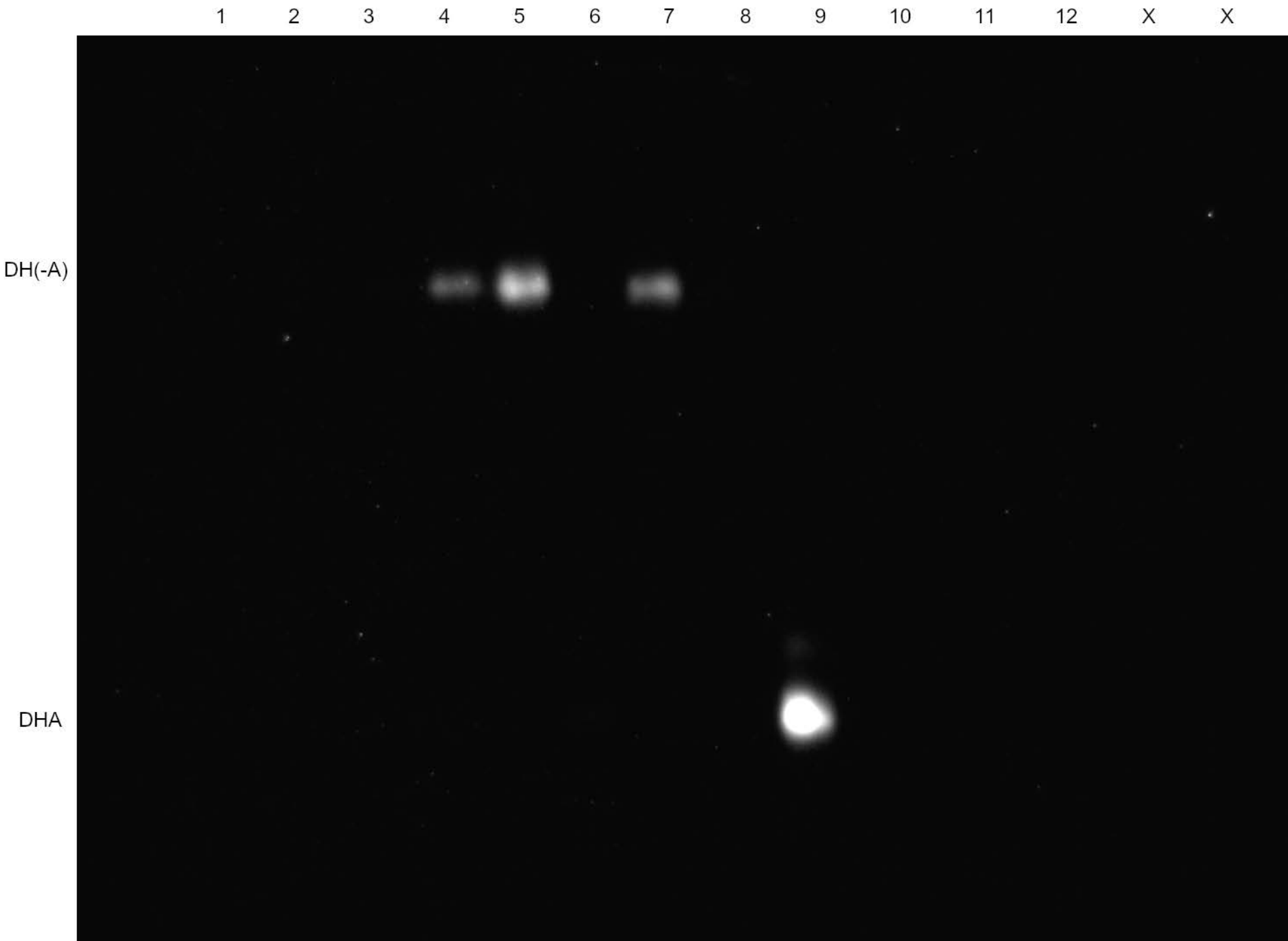

S4 Fig panel A. Contrast-enhanced image of the same membrane.
This image is shown only to visualize the molecular weight marker lane (M) and was not used for PrPres band interpretation.

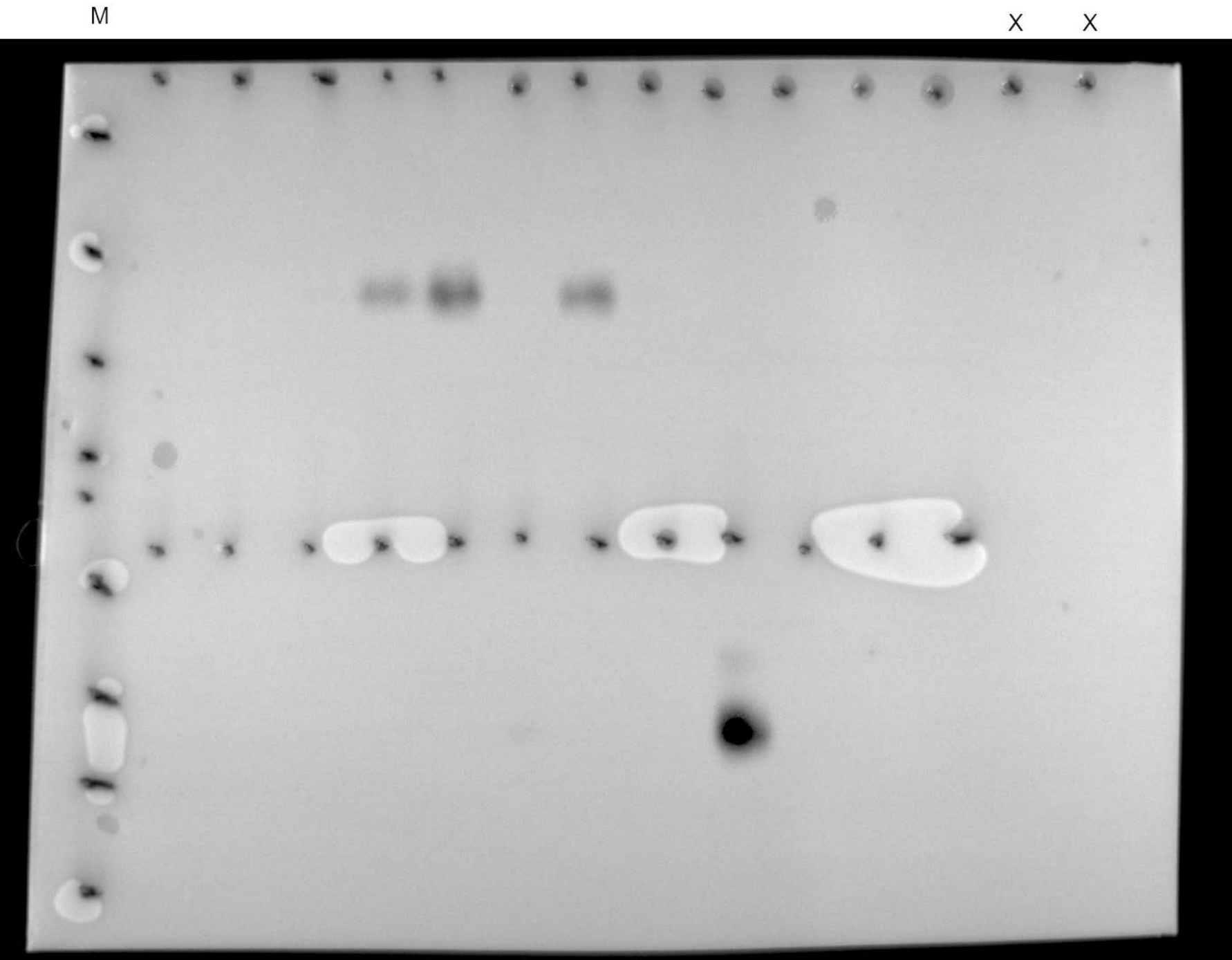

S4 Fig, panel B. Original uncropped chemiluminescence image.

Two gels were transferred onto a single PVDF membrane and detected simultaneously. This image shows the R13 follow-up PMCA products generated from the R12 products that showed faint band-like signals in S4 Fig, panel A. The lanes used for S4 Fig, panel B correspond to DH(-A) lanes 4, 5, and 7 and DHA lane 9 in panel A. Only the indicated lanes in the upper gel image were used for S4 Fig, panel B; the lower gel image was not used in the final figure. Lanes marked with X were not used in the final figure.

The molecular weight marker lane (M) is indicated on the left.

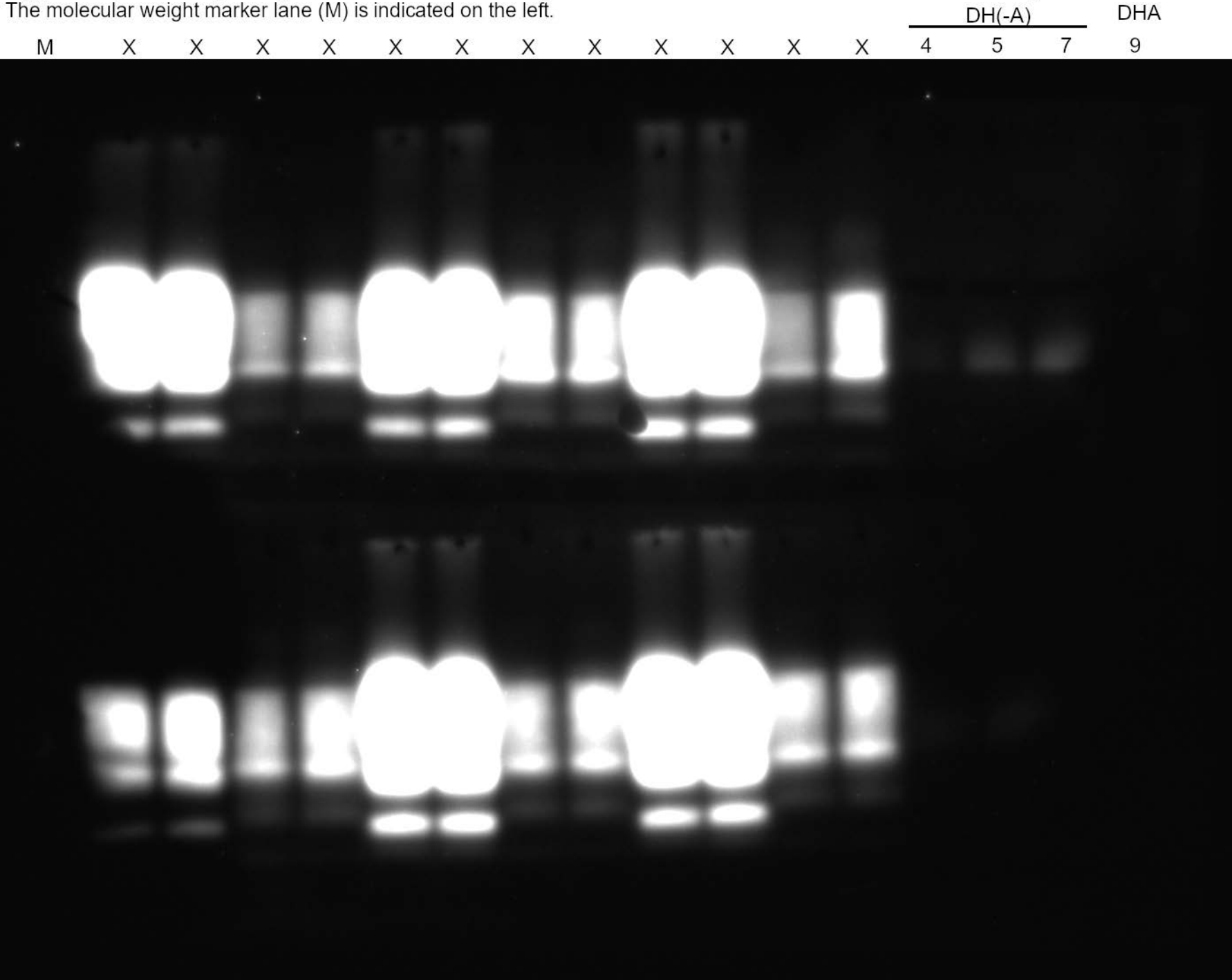

S4 Fig, panel B. Contrast-enhanced image of the same membrane.
This contrast-enhanced image was generated only to visualize the molecular weight marker lane (M) and assign molecular-weight positions; it was not used to evaluate PrPres chemiluminescent signals. The molecular weight marker lane (M) is indicated on the left.

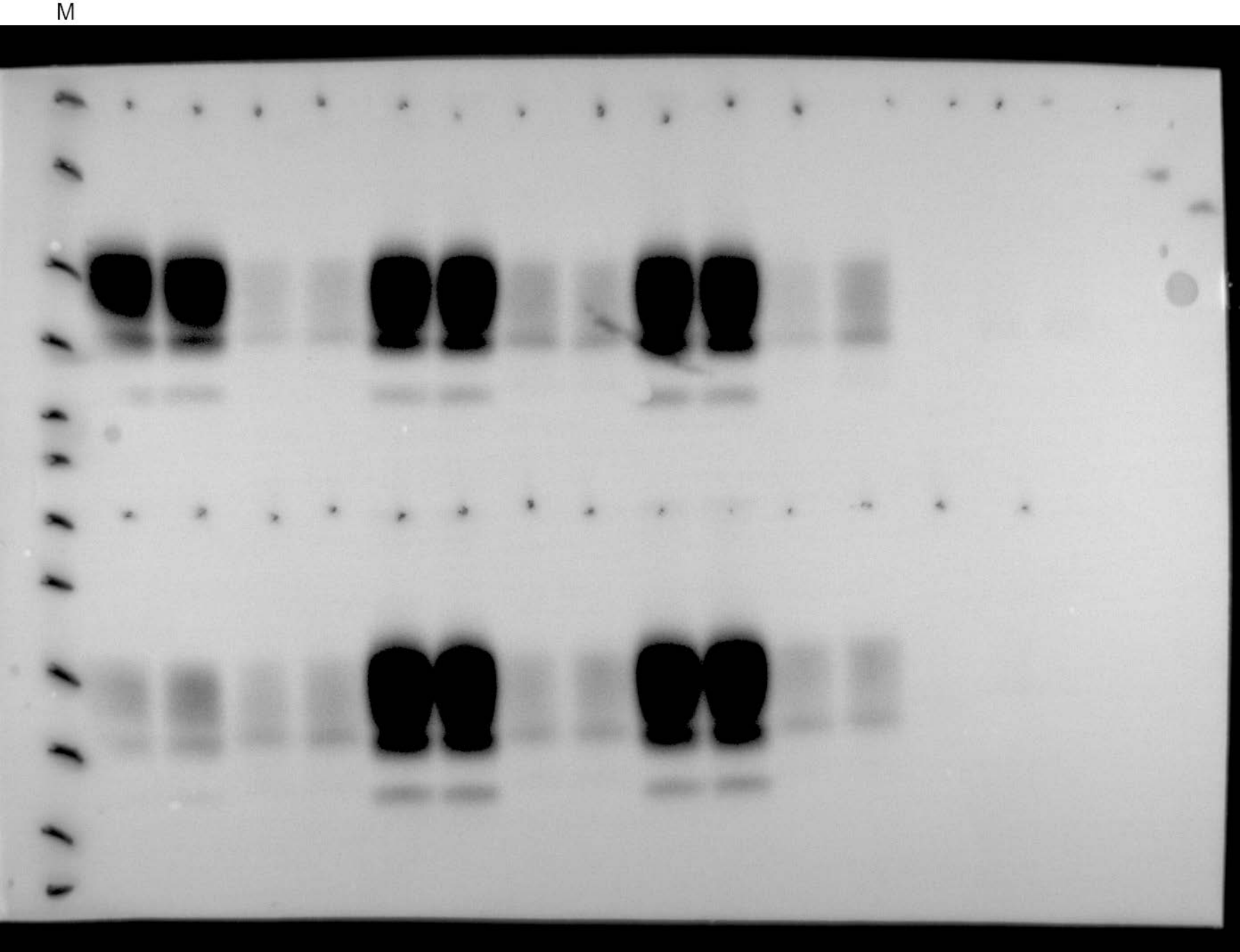

S5 Fig. Original uncropped chemiluminescence image.
Two gels were transferred onto a single PVDF membrane and detected simultaneously.
This image shows the R3 and R6 results corresponding to S4 Fig.
The molecular weight marker lane (M) is indicated on the left.

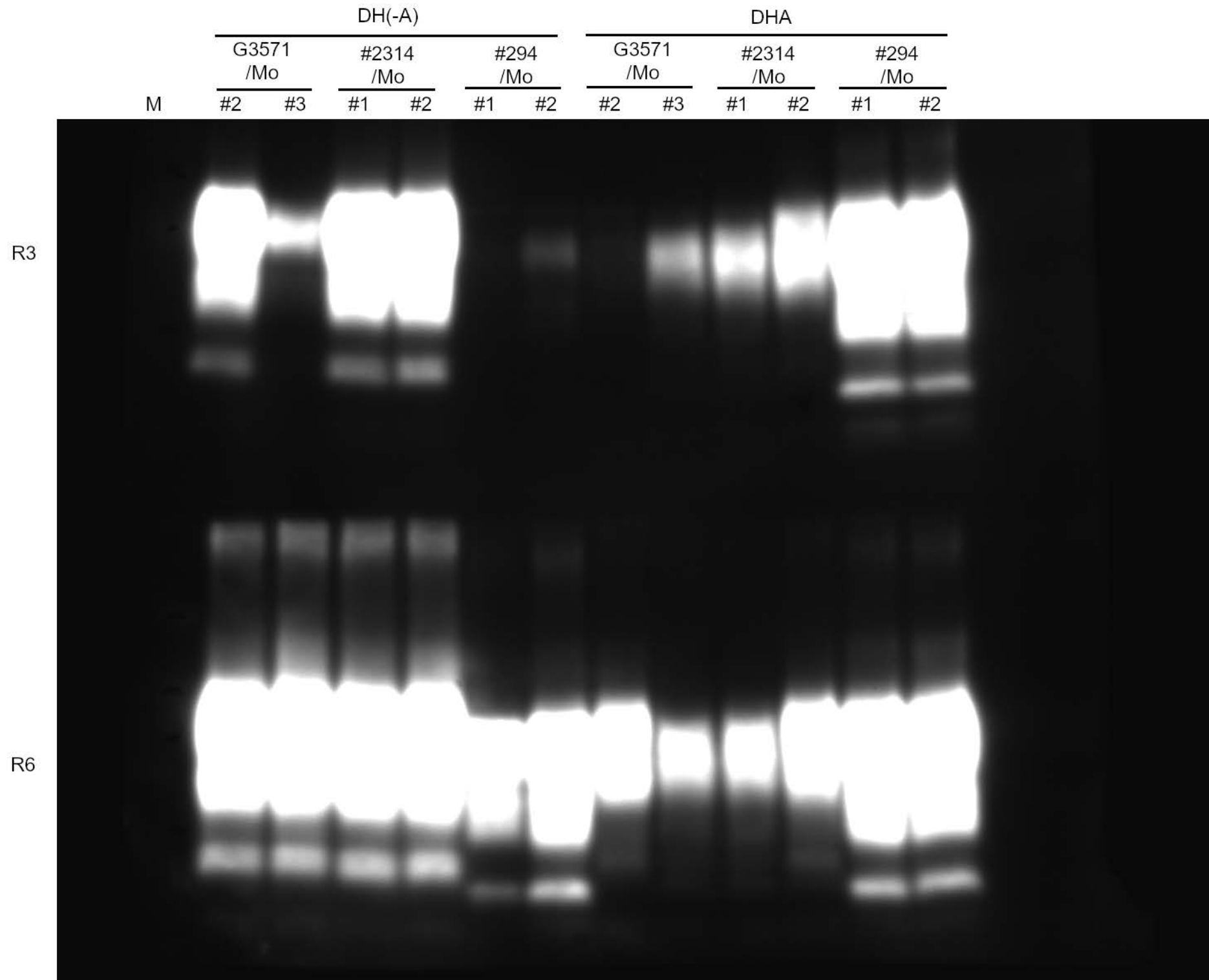

S5 Fig. Contrast-enhanced image of the same membrane.
This image shows the R3 and R6 results corresponding to S4 Fig.
This image was used only to visualize the molecular weight marker lane (M) and assign molecular-weight positions; it was not used to evaluate PrPres chemiluminescent signals.

M

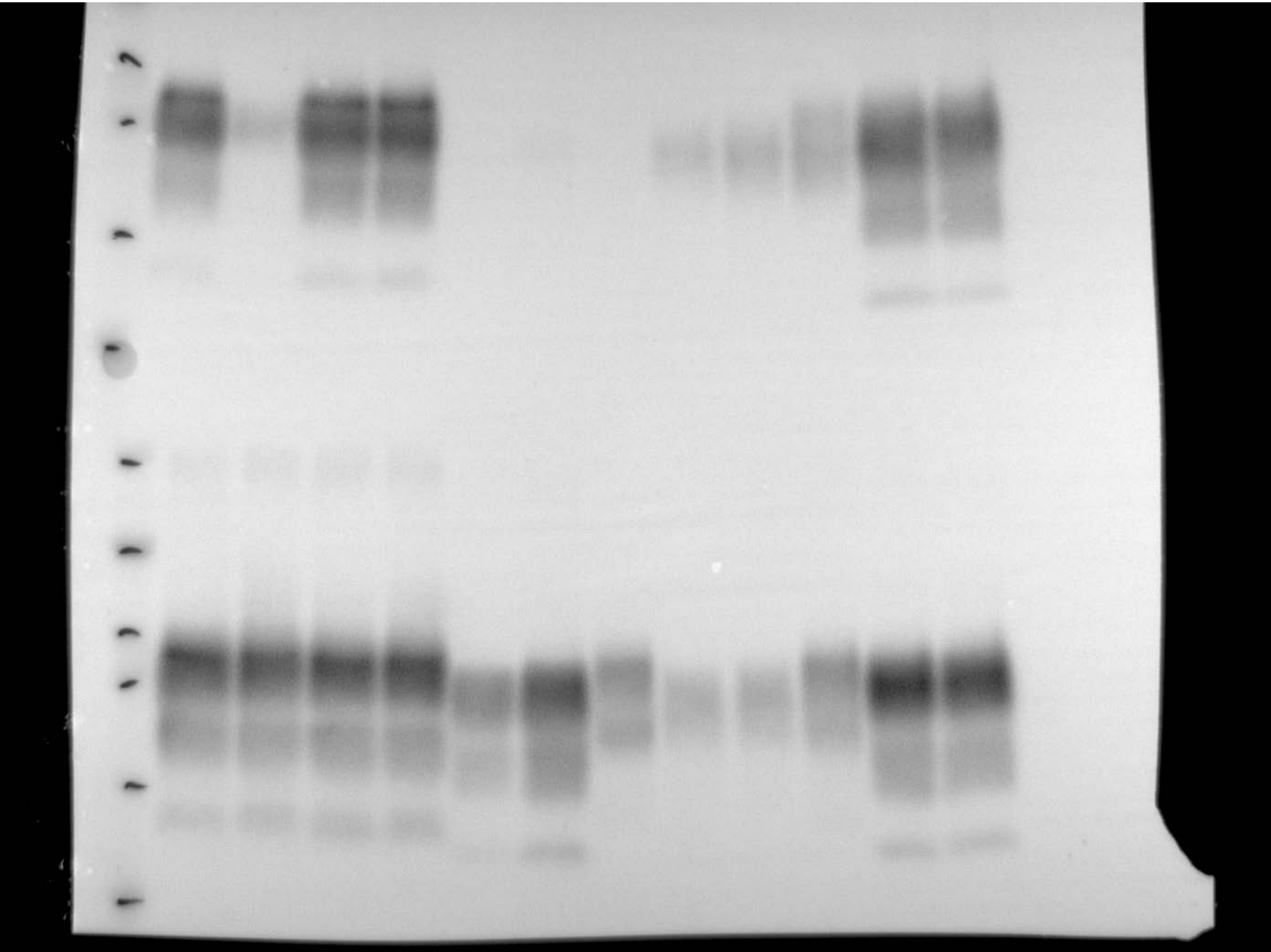

S5 Fig. Original uncropped chemiluminescence image.
This image contains the R12 blot used in S4 Fig.
The molecular weight marker lane (M) is indicated on the left.

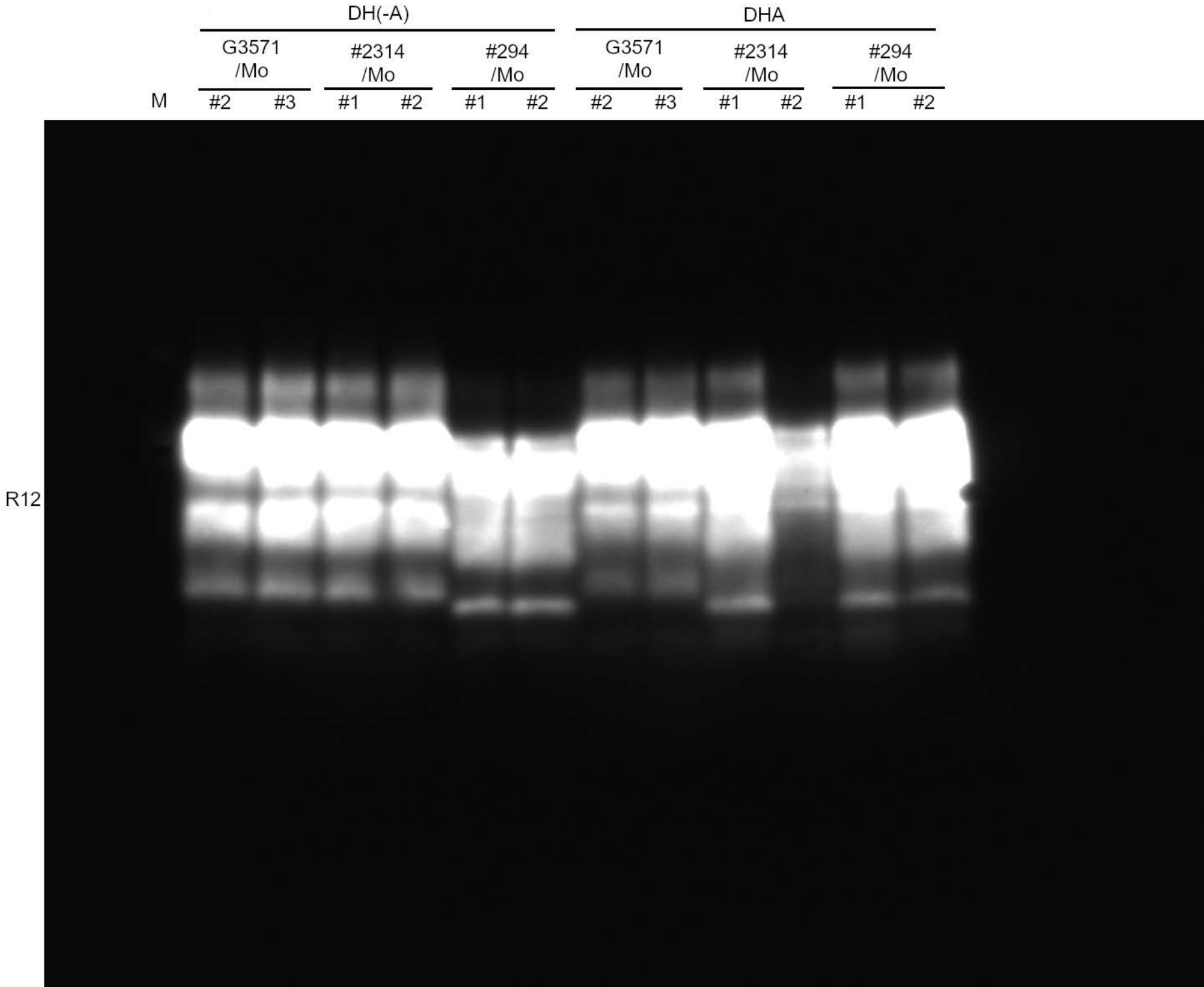

S5 Fig. Contrast-enhanced image of the same membrane.
This image shows the R12 results used in S4 Fig.
This image was used only to visualize the molecular weight marker lane (M) and assign molecular-weight positions;
it was not used to evaluate PrPres chemiluminescent signals.

M

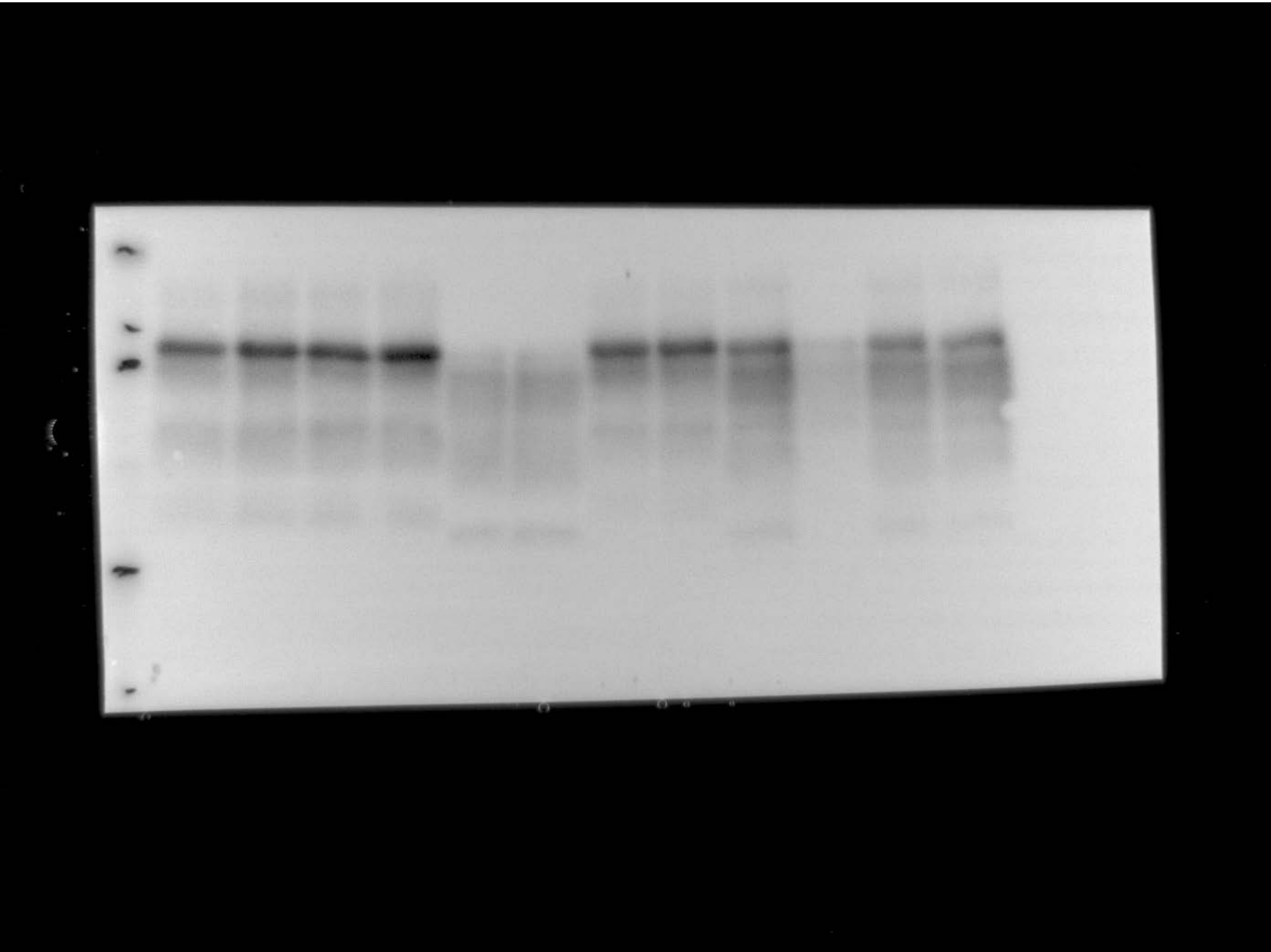

Supplement: Supplementary file 6 [file ppat.1014283.s007.pdf]
